# Supplementary figures and images for: Heterozygosity for neurodevelopmental disorder-associated TRIO variants yields distinct deficits in behavior, neuronal development, and synaptic transmission in mice
Source: eLife. 2025 Jun 9;13:RP103620. doi: 10.7554/eLife.103620 (PMC12148328; doi:10.7554/eLife.103620)

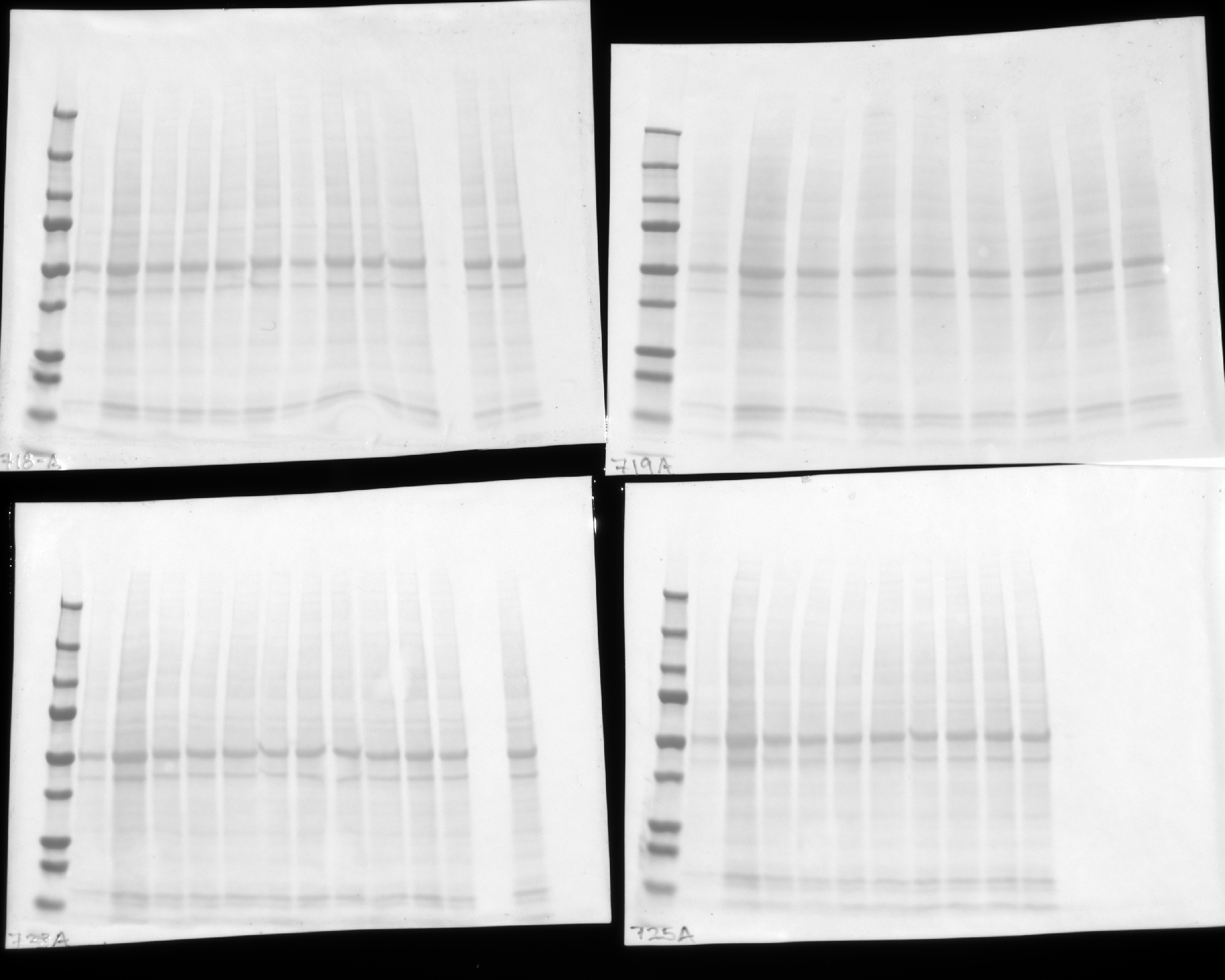

Supplement: Figure 1—source data 1. [file elife-103620-fig1-data1.zip › Figure 1-source data 1/P0 brain Ponceau.tif]

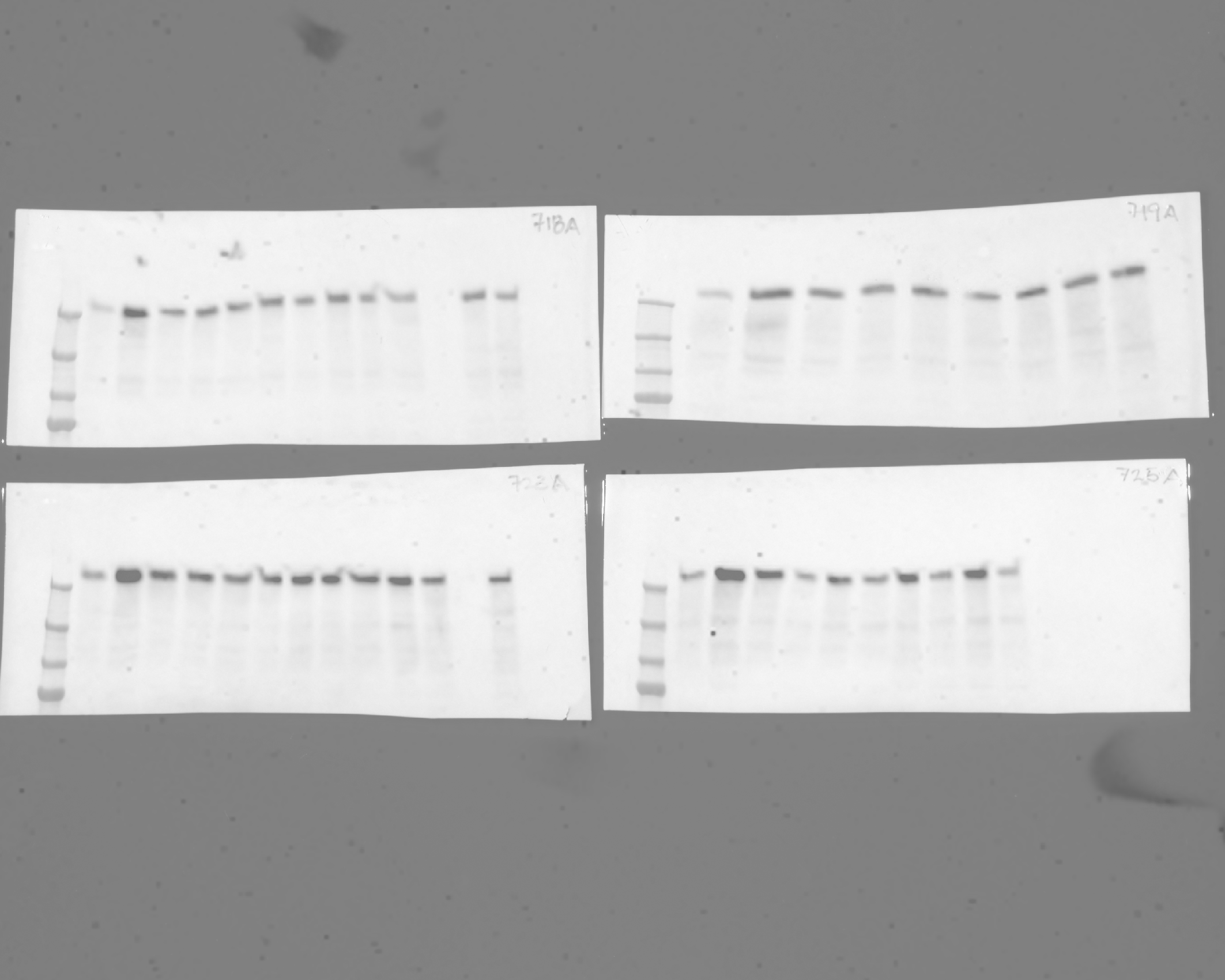

Supplement: Figure 1—source data 1. [file elife-103620-fig1-data1.zip › Figure 1-source data 1/P0 brain WB TrioSR56.tif]

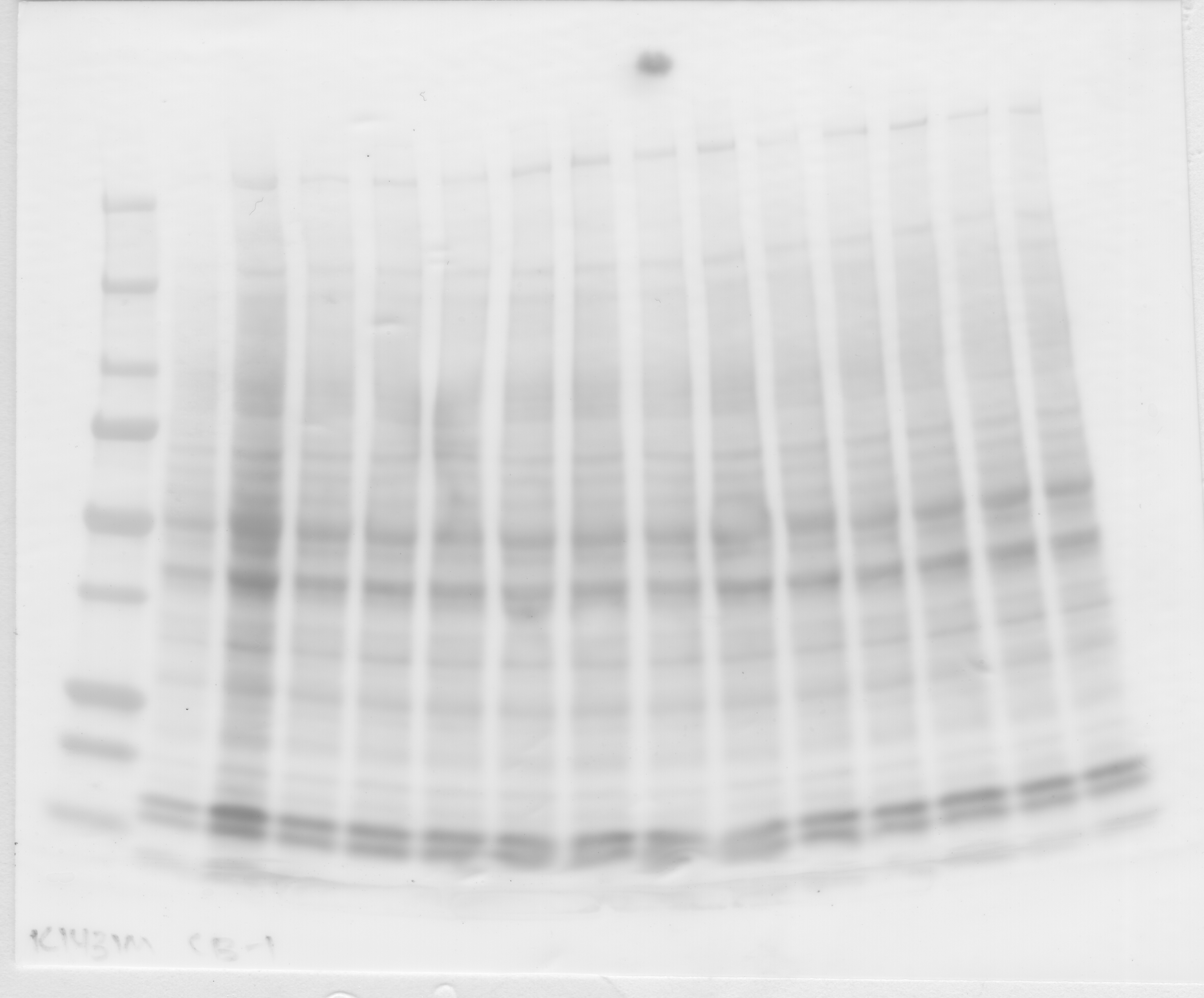

Supplement: Figure 1—figure supplement 1—source data 1. [file elife-103620-fig1-figsupp1-data1.zip › Figure 1-figure supplement 1-source data 1/K1431M CB1 Ponceau.tif]

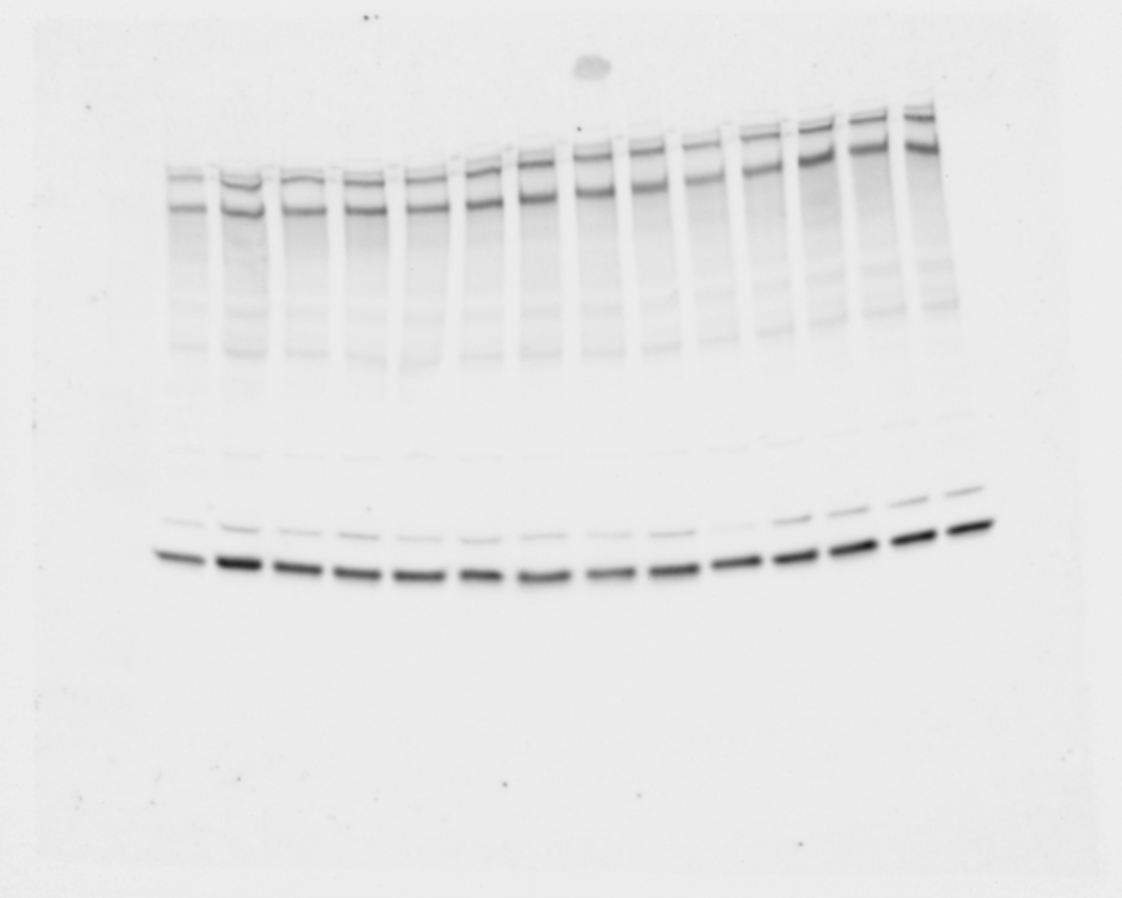

Supplement: Figure 1—figure supplement 1—source data 1. [file elife-103620-fig1-figsupp1-data1.zip › Figure 1-figure supplement 1-source data 1/K1431M CB1 WB TrioSR56.tif]

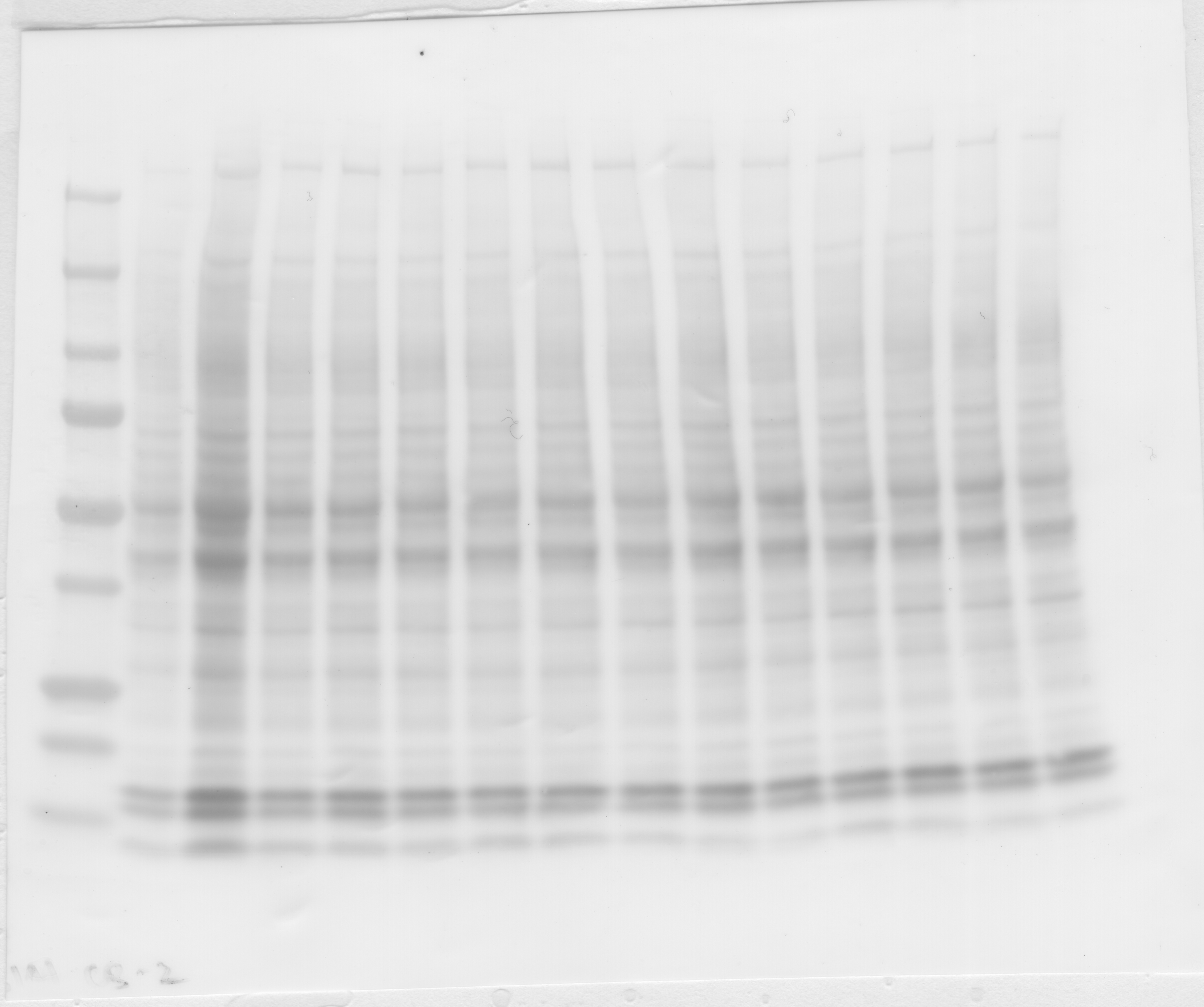

Supplement: Figure 1—figure supplement 1—source data 1. [file elife-103620-fig1-figsupp1-data1.zip › Figure 1-figure supplement 1-source data 1/K1431M CB2 Ponceau.tif]

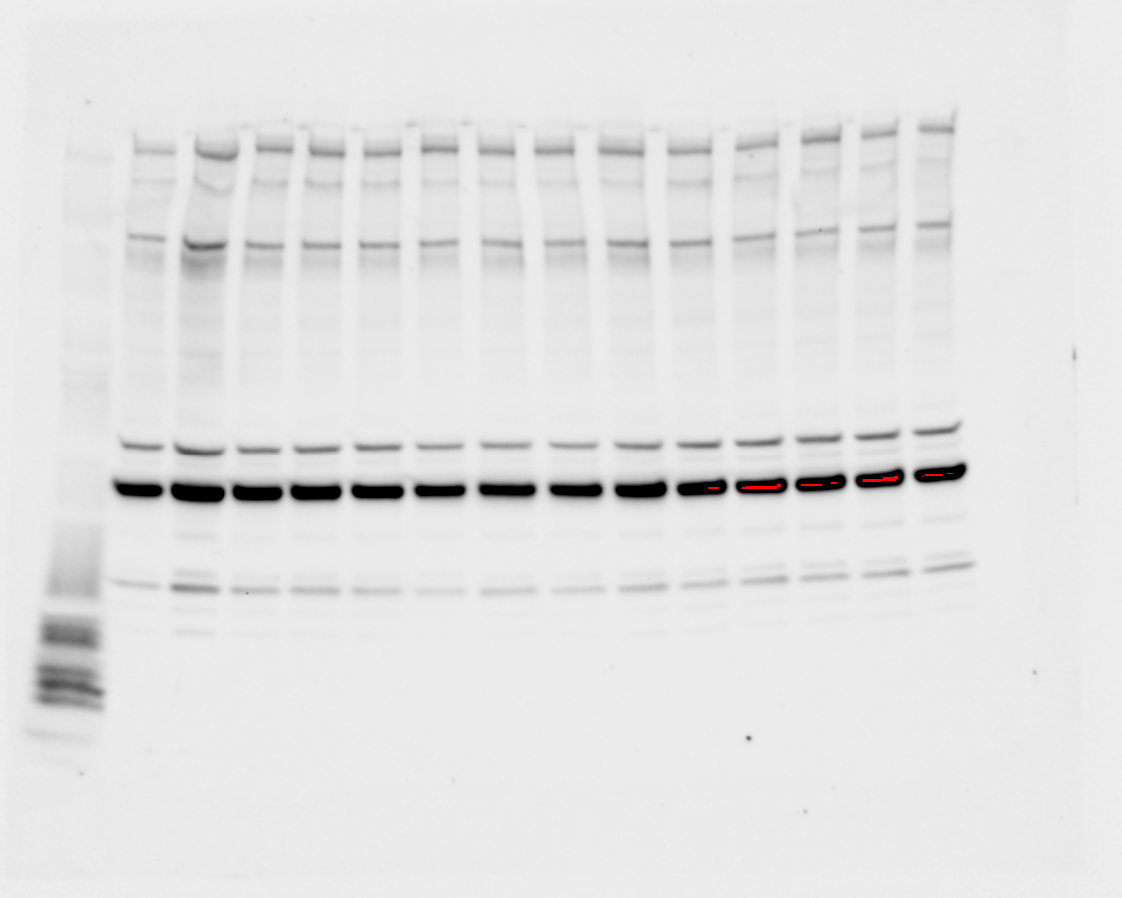

Supplement: Figure 1—figure supplement 1—source data 1. [file elife-103620-fig1-figsupp1-data1.zip › Figure 1-figure supplement 1-source data 1/K1431M CB2 WB TrioDH2.tif]

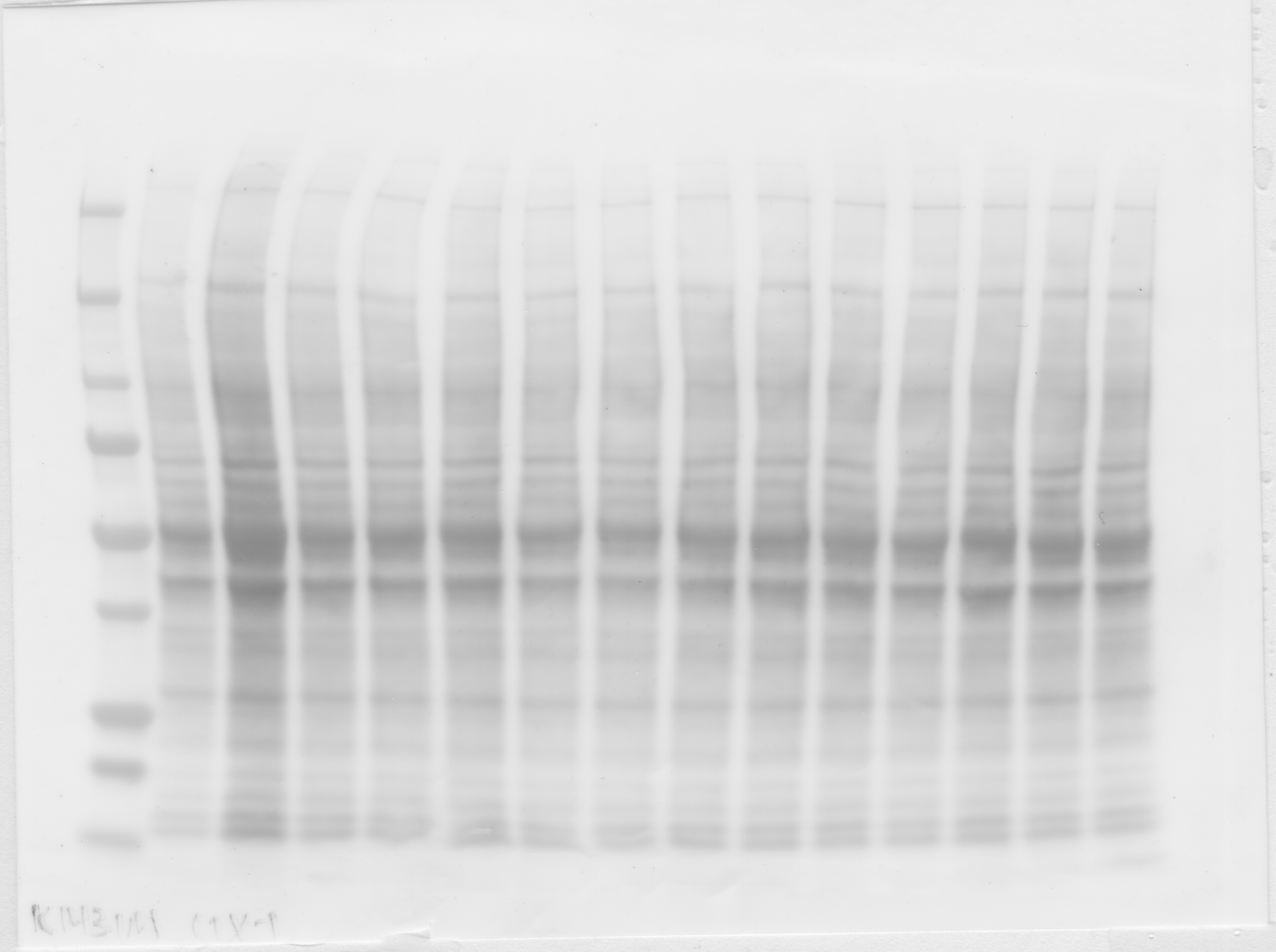

Supplement: Figure 1—figure supplement 1—source data 1. [file elife-103620-fig1-figsupp1-data1.zip › Figure 1-figure supplement 1-source data 1/K1431M CTX1 Ponceau.tif]

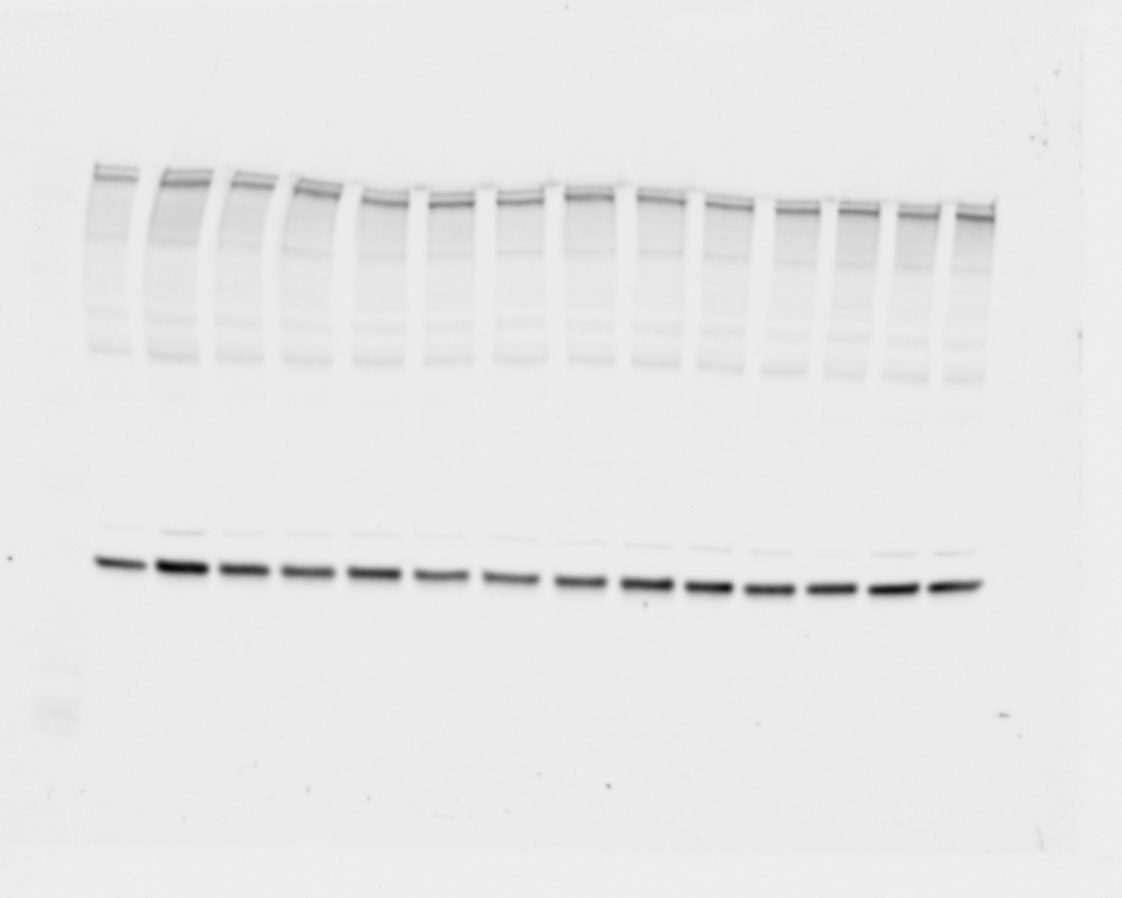

Supplement: Figure 1—figure supplement 1—source data 1. [file elife-103620-fig1-figsupp1-data1.zip › Figure 1-figure supplement 1-source data 1/K1431M CTX1 WB TrioSR56.tif]

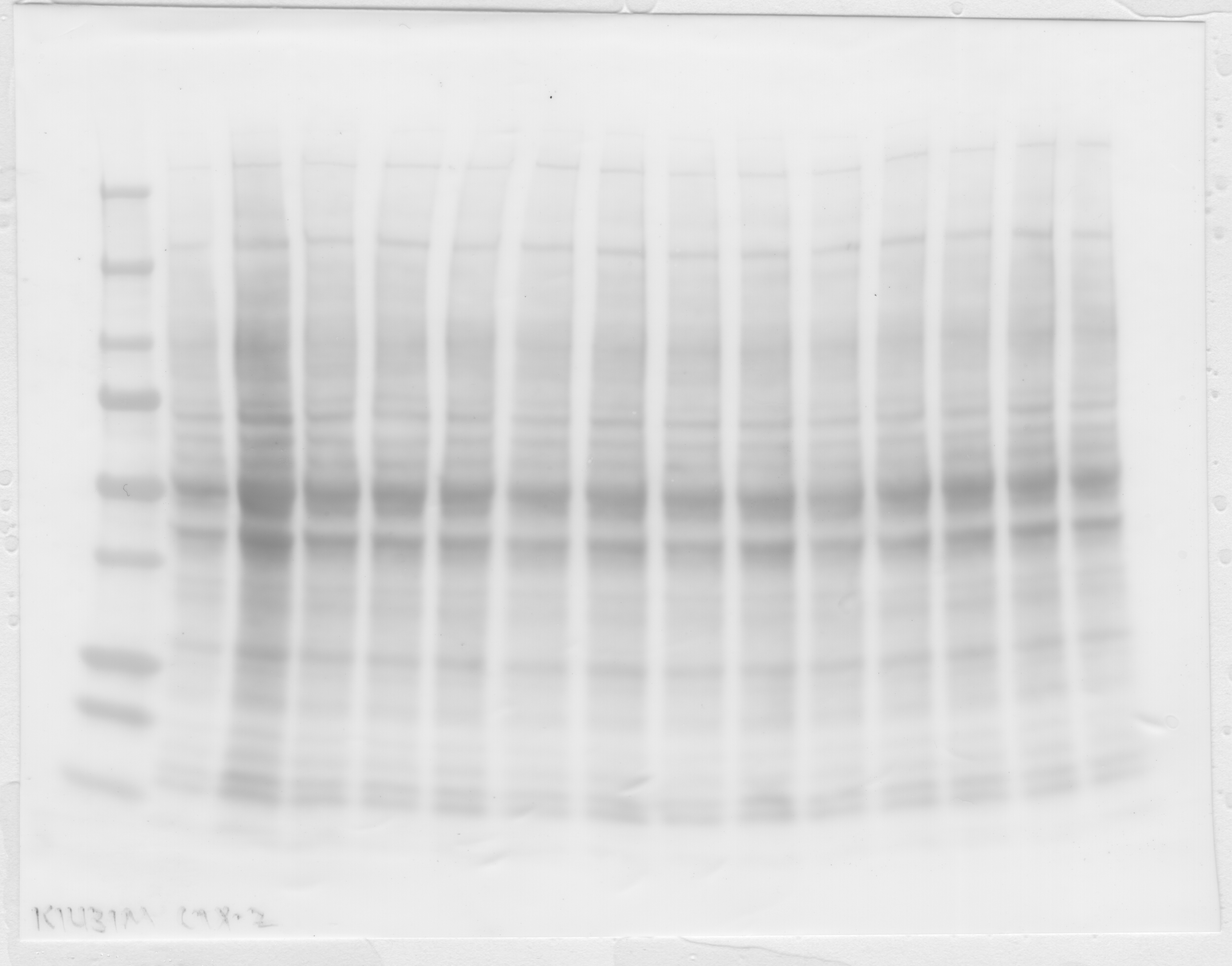

Supplement: Figure 1—figure supplement 1—source data 1. [file elife-103620-fig1-figsupp1-data1.zip › Figure 1-figure supplement 1-source data 1/K1431M CTX2 Ponceau.tif]

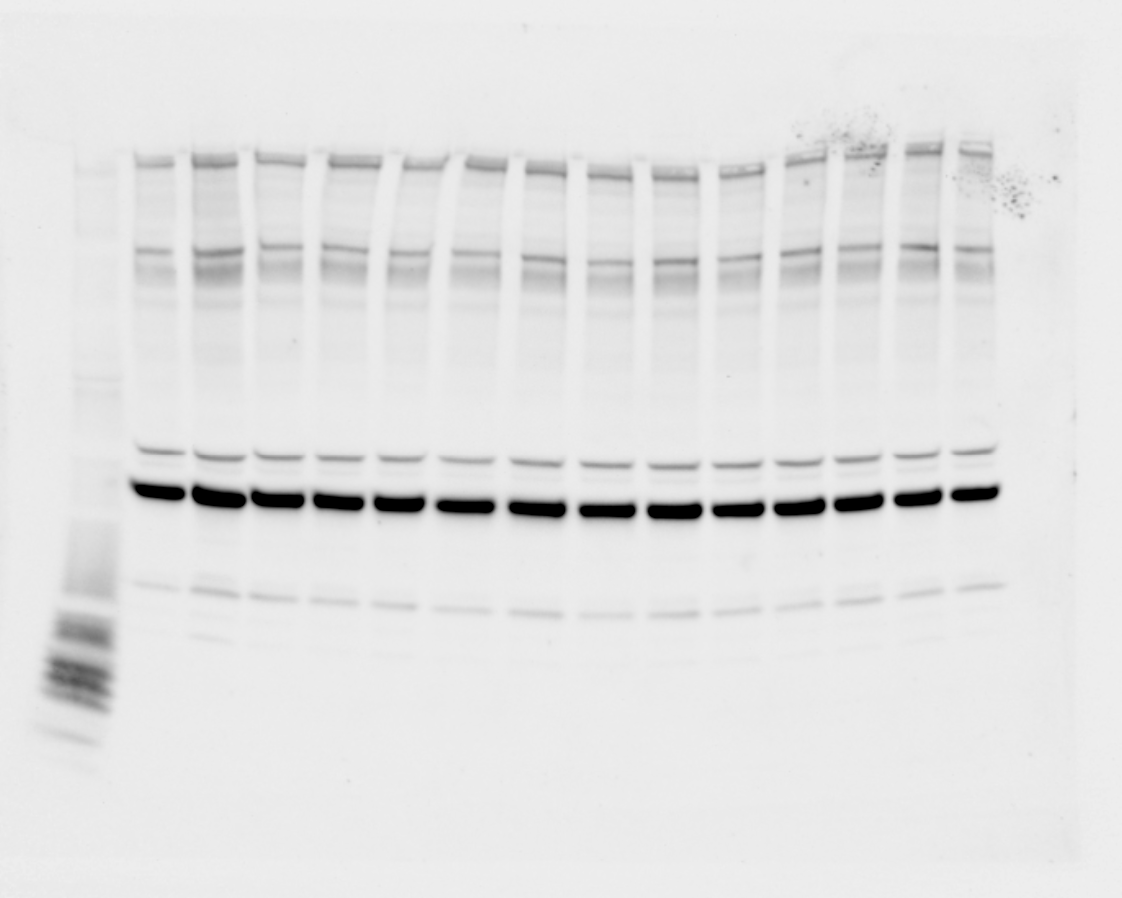

Supplement: Figure 1—figure supplement 1—source data 1. [file elife-103620-fig1-figsupp1-data1.zip › Figure 1-figure supplement 1-source data 1/K1431M CTX2 WB TrioDH2.tif]

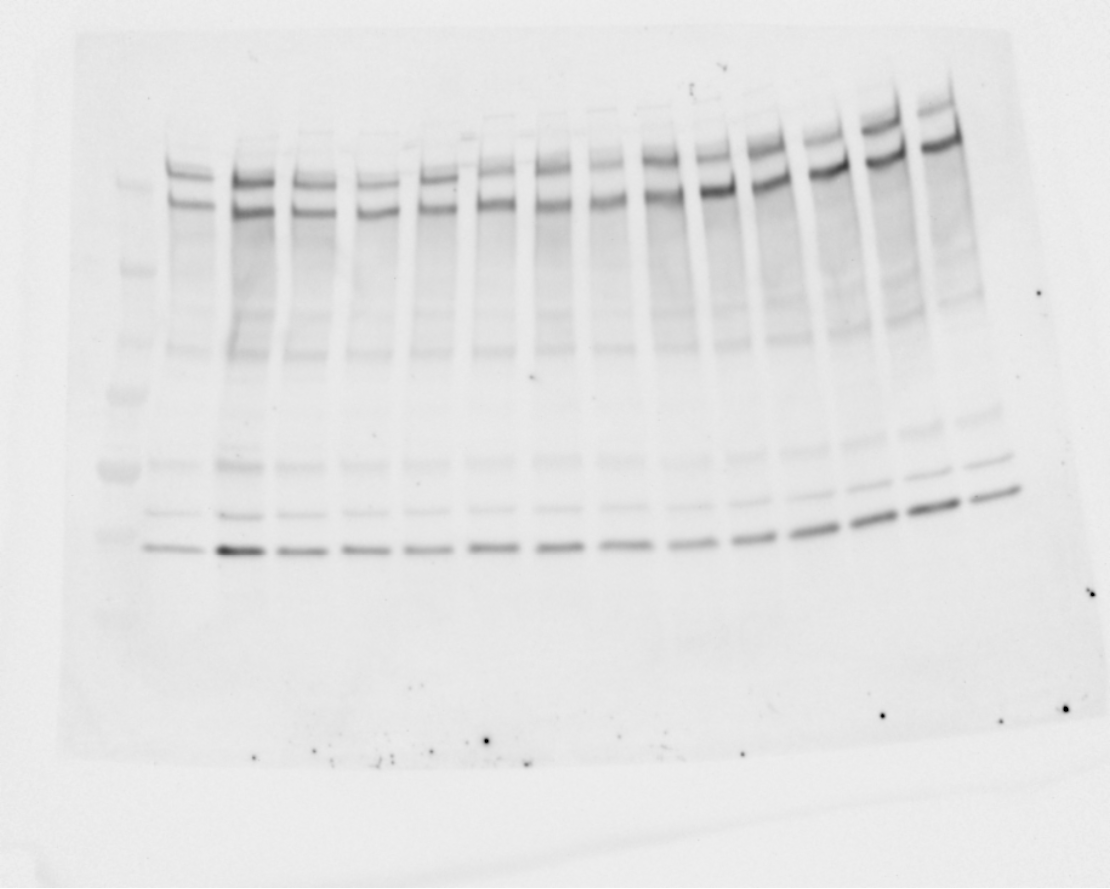

Supplement: Figure 1—figure supplement 1—source data 1. [file elife-103620-fig1-figsupp1-data1.zip › Figure 1-figure supplement 1-source data 1/K1918X CB1 WB TrioSR56.tif]

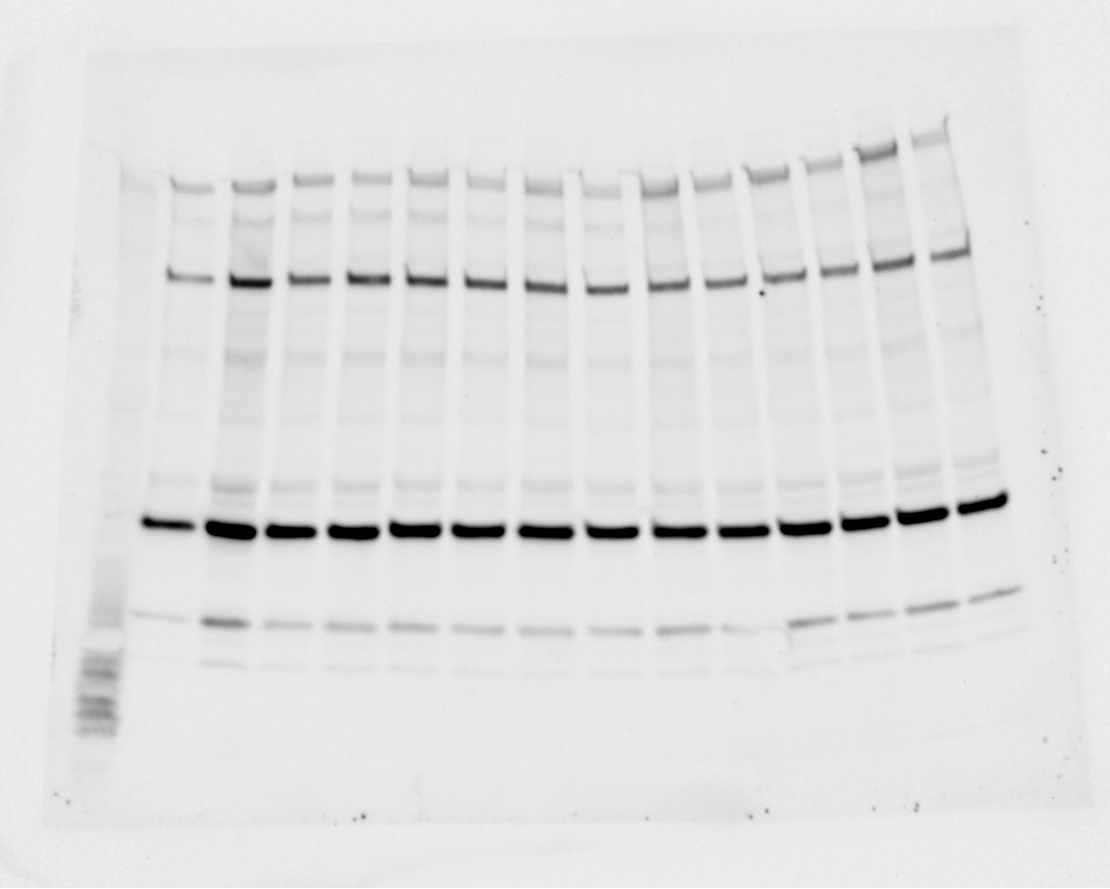

Supplement: Figure 1—figure supplement 1—source data 1. [file elife-103620-fig1-figsupp1-data1.zip › Figure 1-figure supplement 1-source data 1/K1918X CB2 WB TrioDH2.tif]

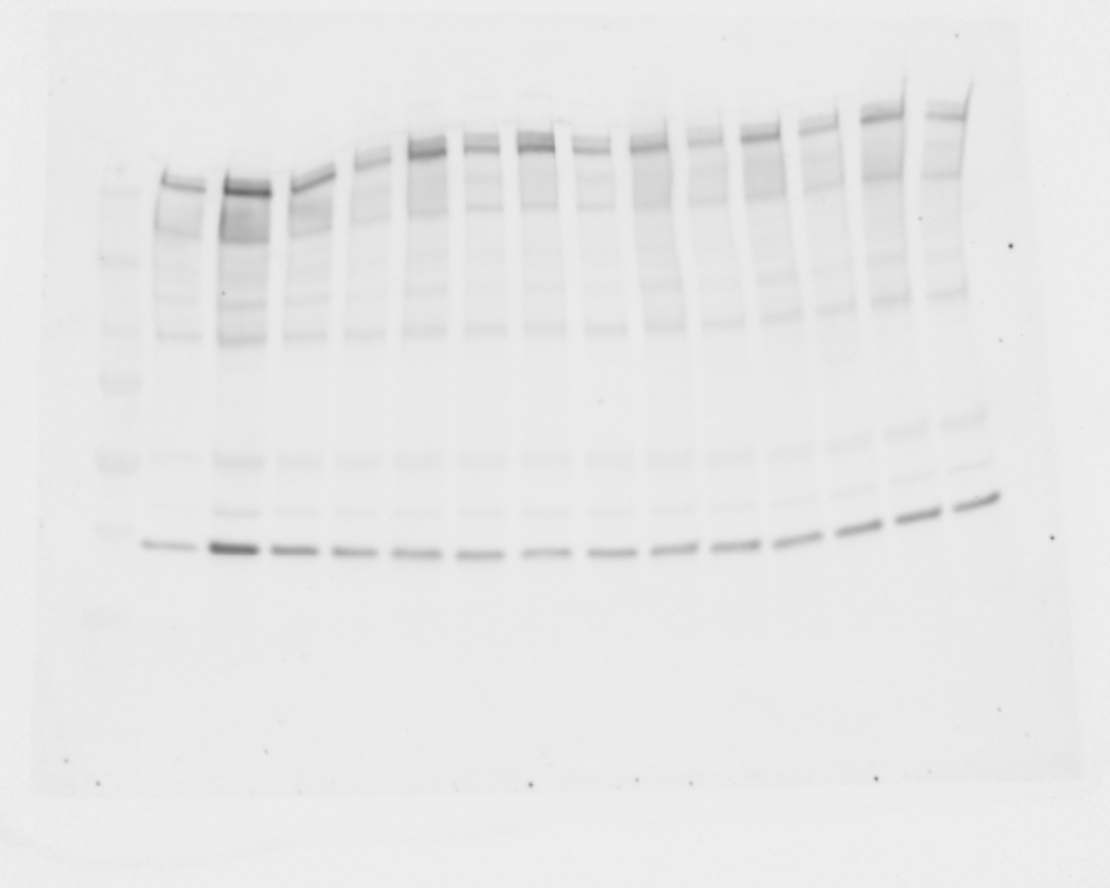

Supplement: Figure 1—figure supplement 1—source data 1. [file elife-103620-fig1-figsupp1-data1.zip › Figure 1-figure supplement 1-source data 1/K1918X CTX1 WB TrioSR56.tif]

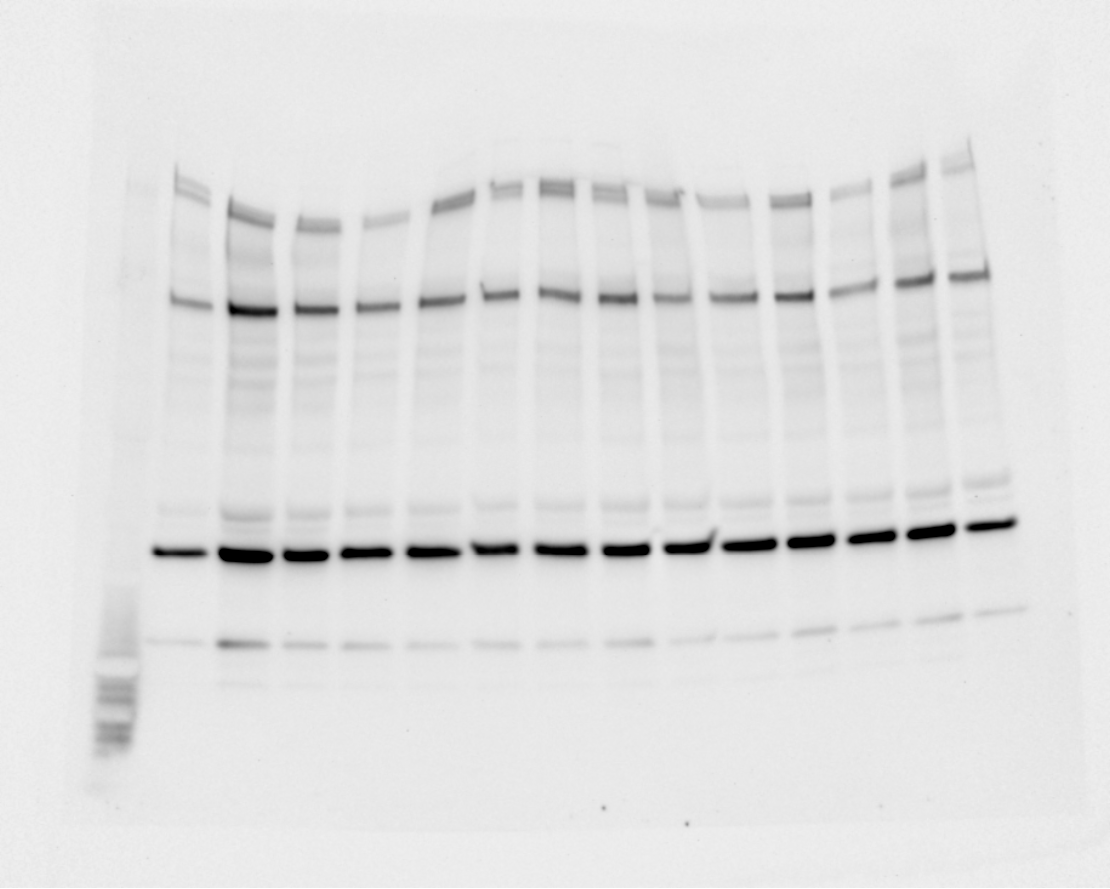

Supplement: Figure 1—figure supplement 1—source data 1. [file elife-103620-fig1-figsupp1-data1.zip › Figure 1-figure supplement 1-source data 1/K1918X CTX2 WB TrioDH2.tif]

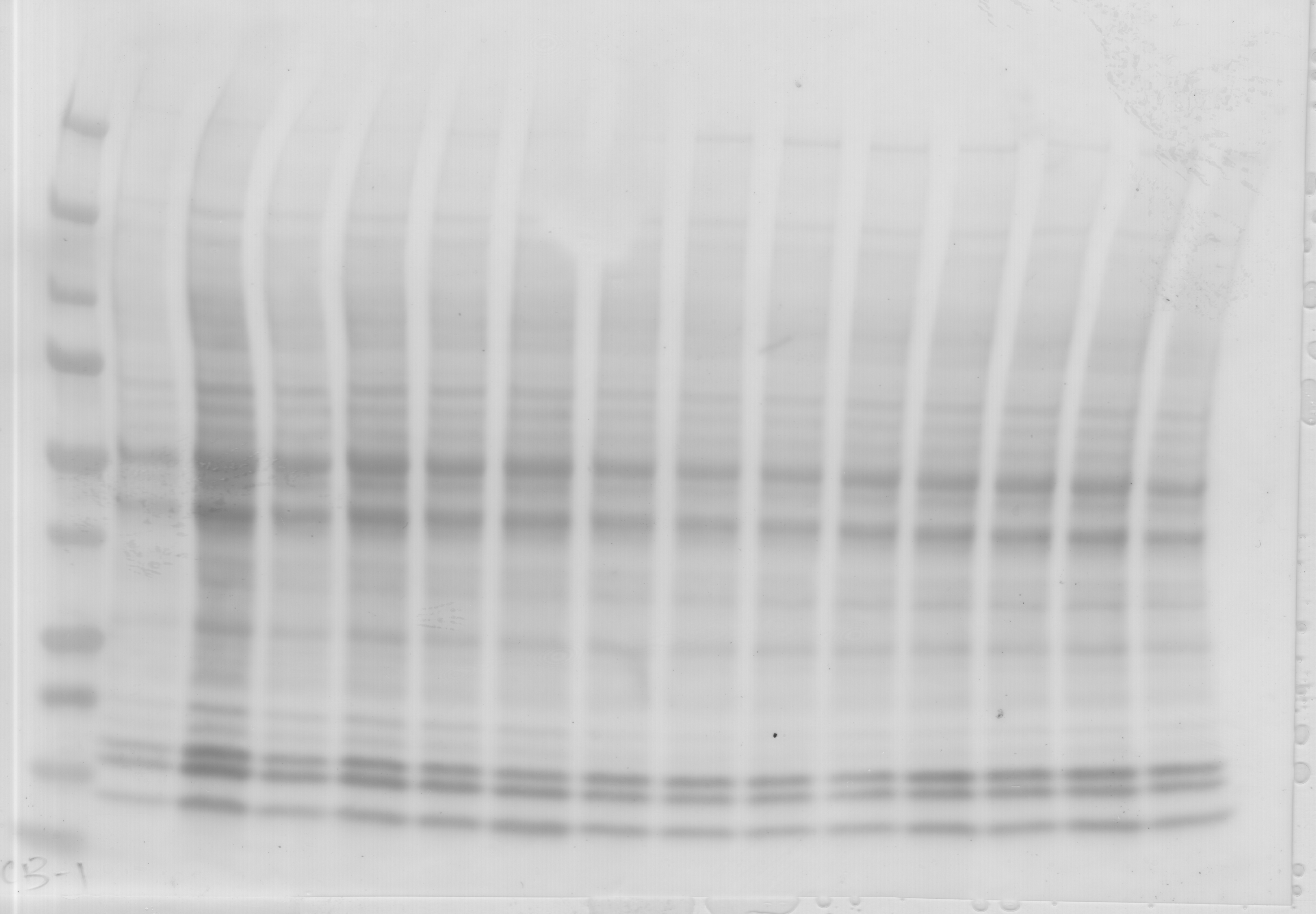

Supplement: Figure 1—figure supplement 1—source data 1. [file elife-103620-fig1-figsupp1-data1.zip › Figure 1-figure supplement 1-source data 1/M2145T CB1 Ponceau.tif]

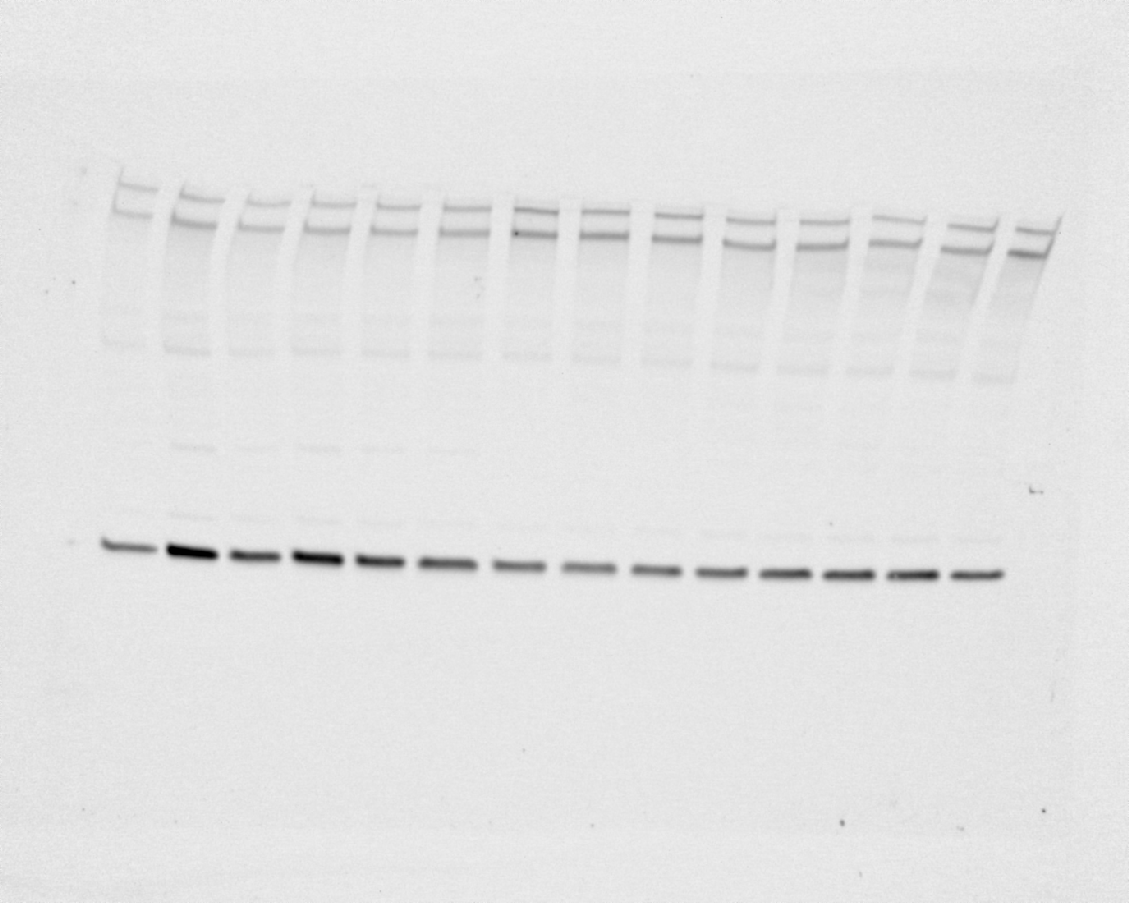

Supplement: Figure 1—figure supplement 1—source data 1. [file elife-103620-fig1-figsupp1-data1.zip › Figure 1-figure supplement 1-source data 1/M2145T CB1 WB TrioSR56.tif]

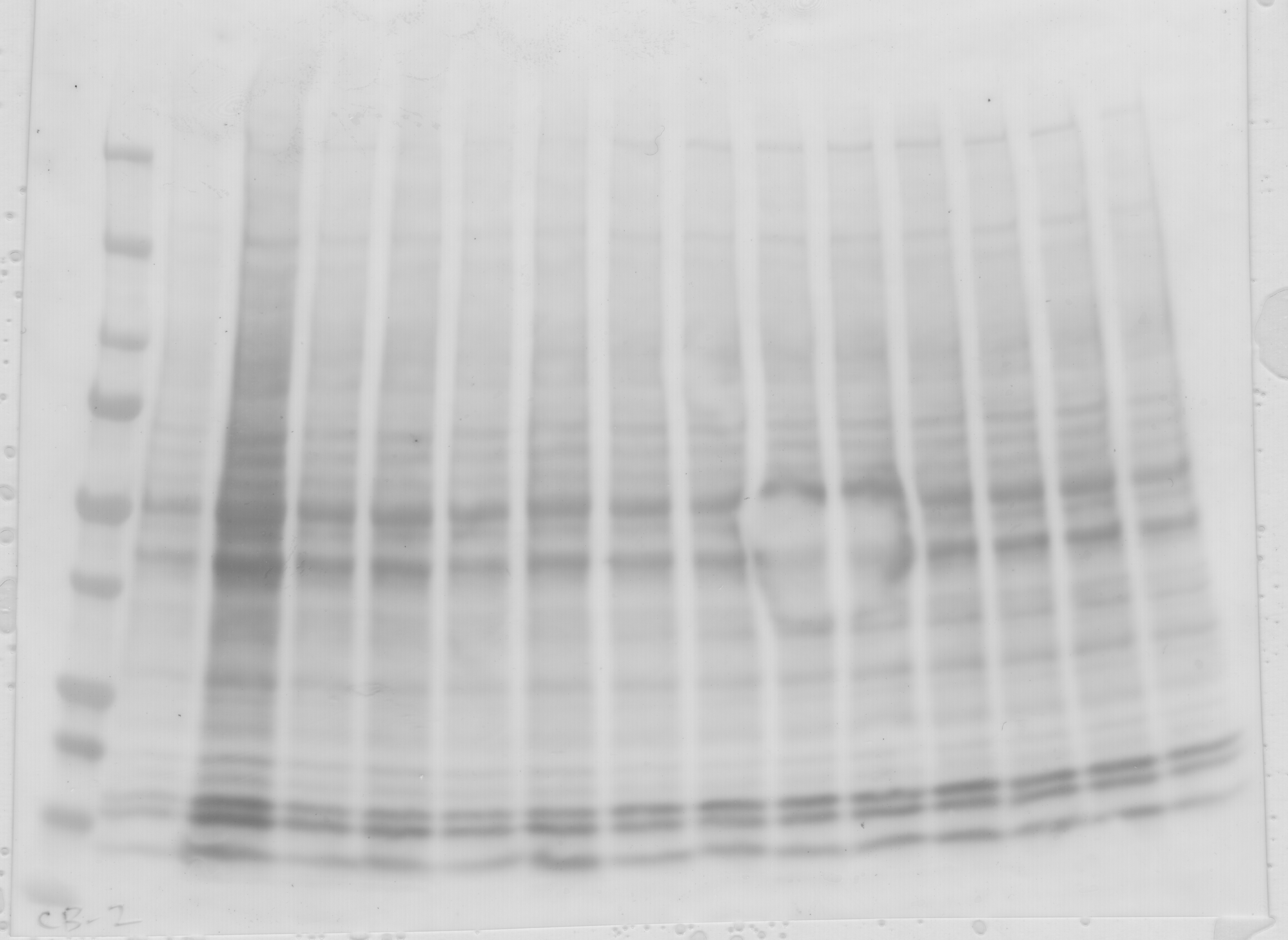

Supplement: Figure 1—figure supplement 1—source data 1. [file elife-103620-fig1-figsupp1-data1.zip › Figure 1-figure supplement 1-source data 1/M2145T CB2 Ponceau.tif]

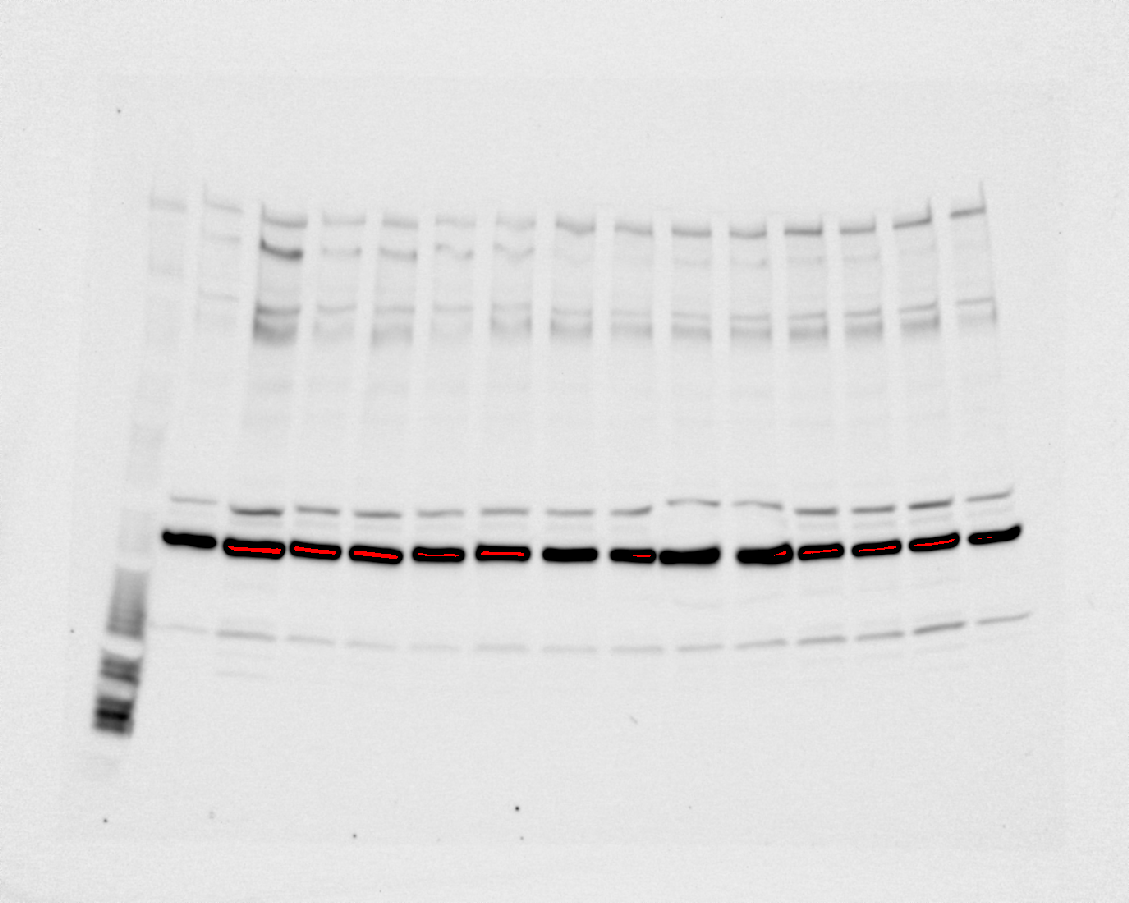

Supplement: Figure 1—figure supplement 1—source data 1. [file elife-103620-fig1-figsupp1-data1.zip › Figure 1-figure supplement 1-source data 1/M2145T CB2 WB TrioDH2.tif]

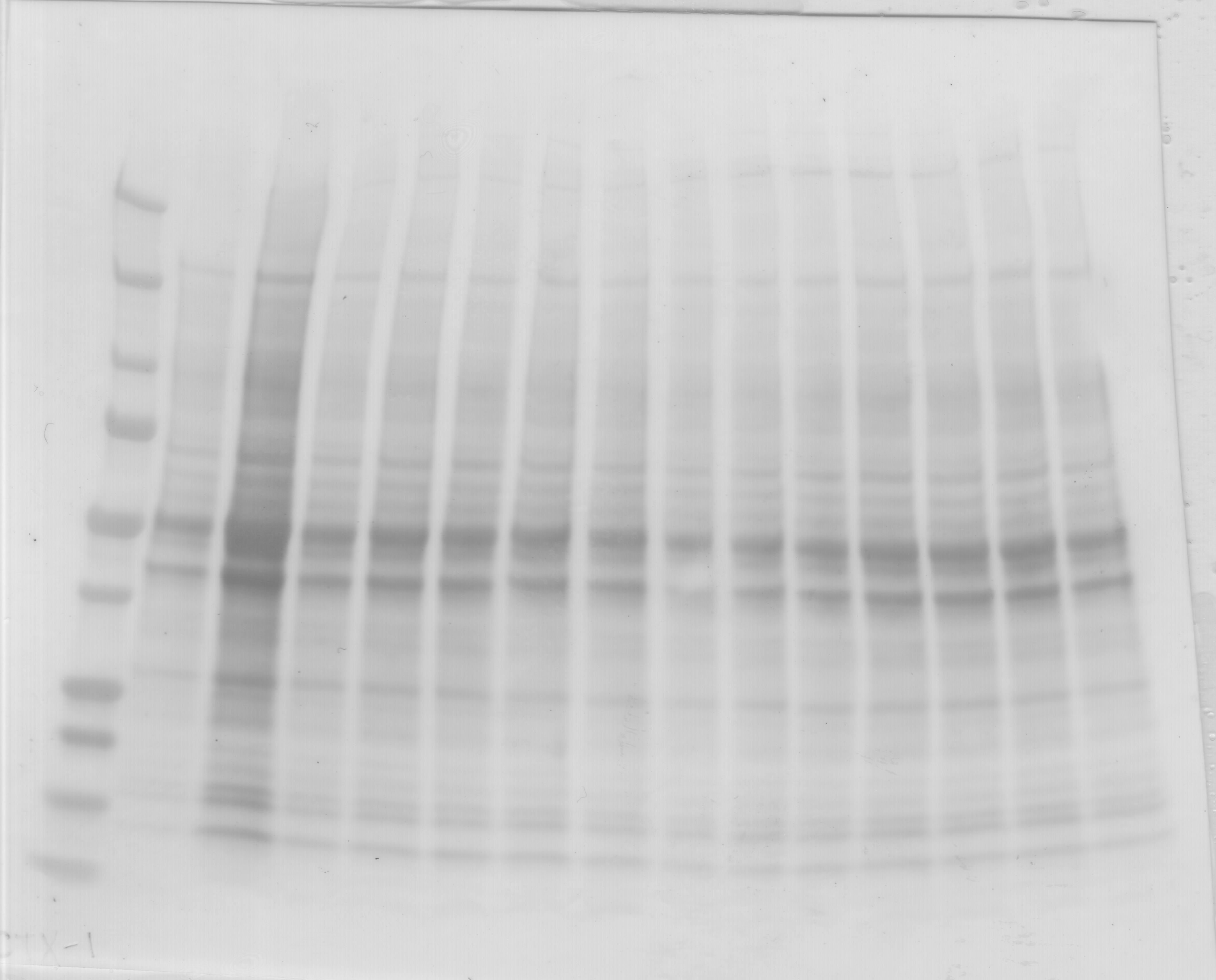

Supplement: Figure 1—figure supplement 1—source data 1. [file elife-103620-fig1-figsupp1-data1.zip › Figure 1-figure supplement 1-source data 1/M2145T CTX1 Ponceau.tif]

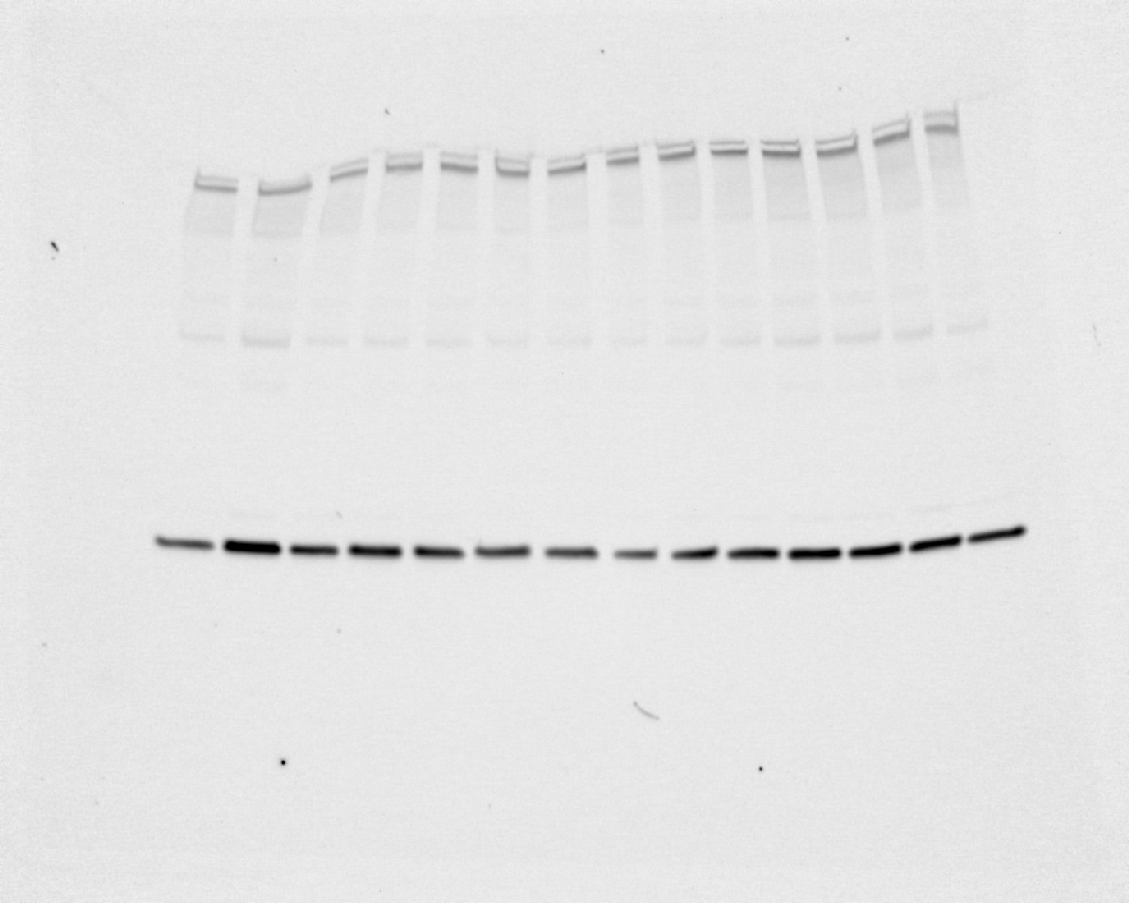

Supplement: Figure 1—figure supplement 1—source data 1. [file elife-103620-fig1-figsupp1-data1.zip › Figure 1-figure supplement 1-source data 1/M2145T CTX1 WB TrioSR56.tif]

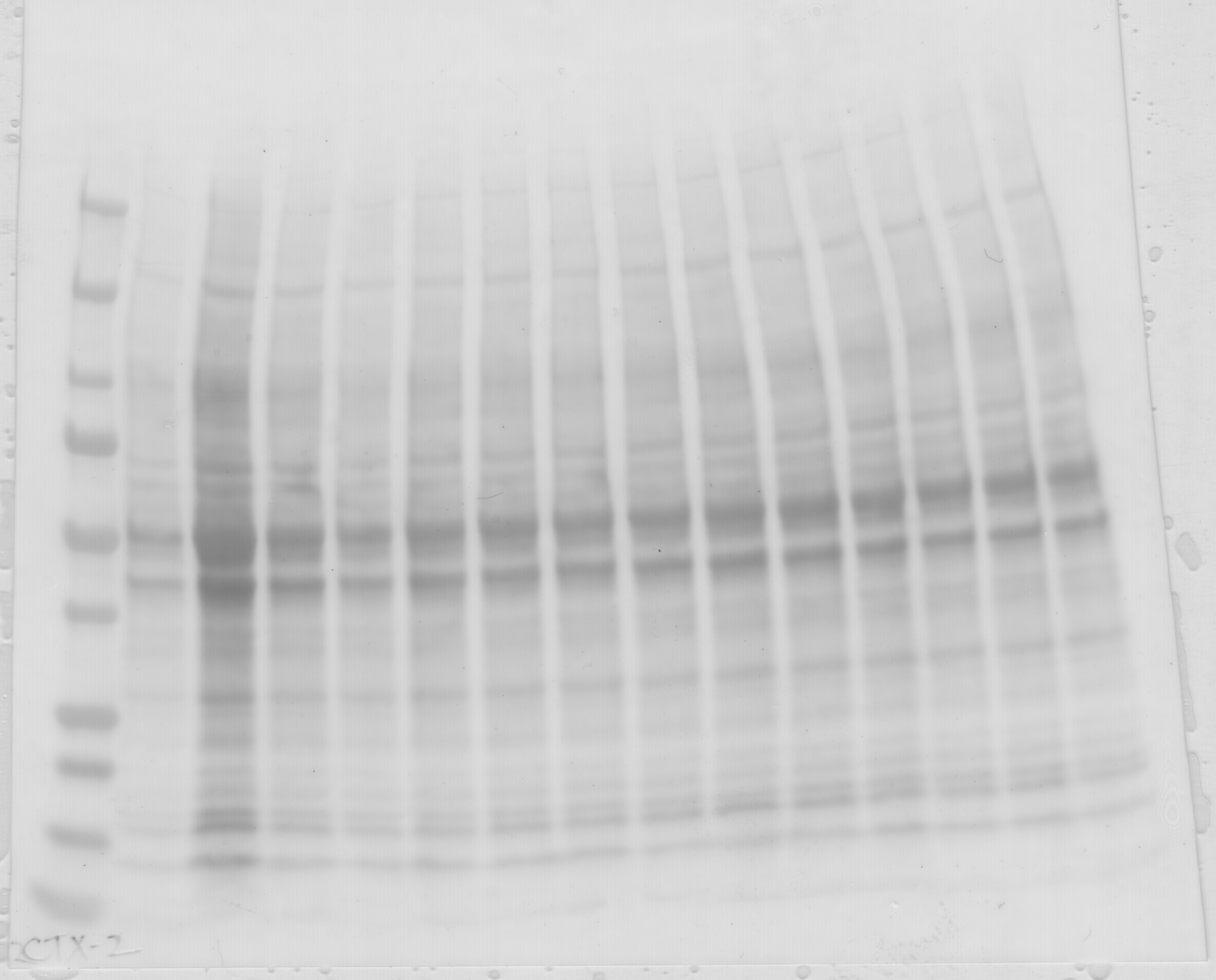

Supplement: Figure 1—figure supplement 1—source data 1. [file elife-103620-fig1-figsupp1-data1.zip › Figure 1-figure supplement 1-source data 1/M2145T CTX2 Ponceau.tif]

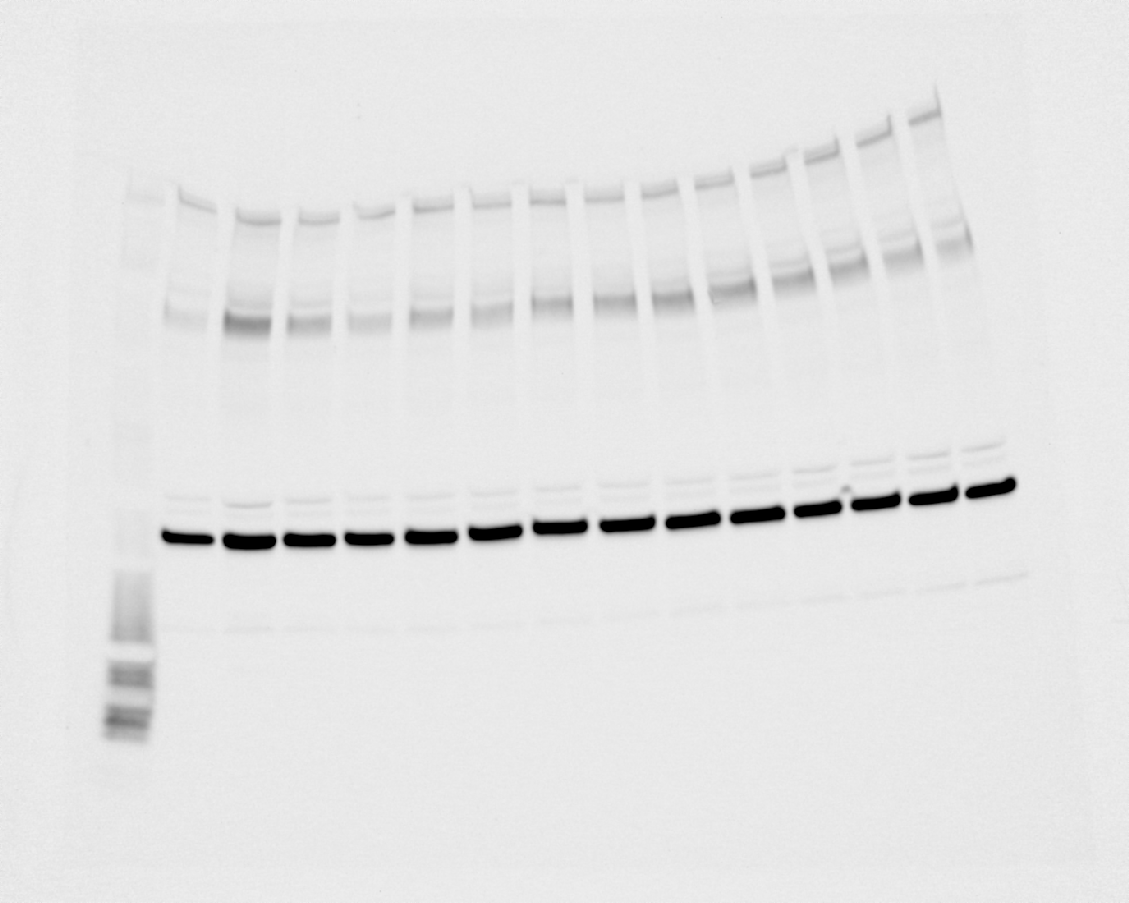

Supplement: Figure 1—figure supplement 1—source data 1. [file elife-103620-fig1-figsupp1-data1.zip › Figure 1-figure supplement 1-source data 1/M2145T CTX2 WB TrioDH2.tif]

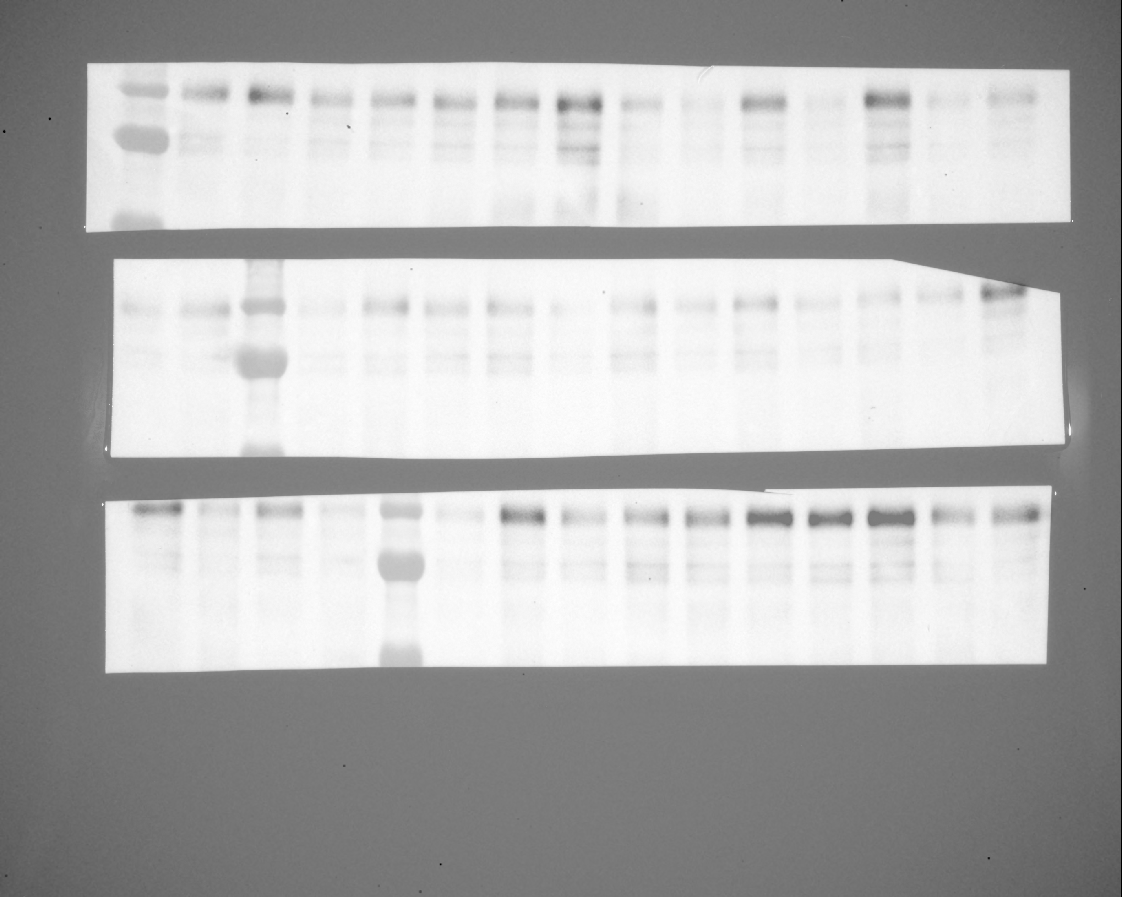

Supplement: Figure 1—figure supplement 1—source data 3. [file elife-103620-fig1-figsupp1-data3.zip › Figure 1-figure supplement 1-source data 3/CTXsyn WB PSD95.tif]

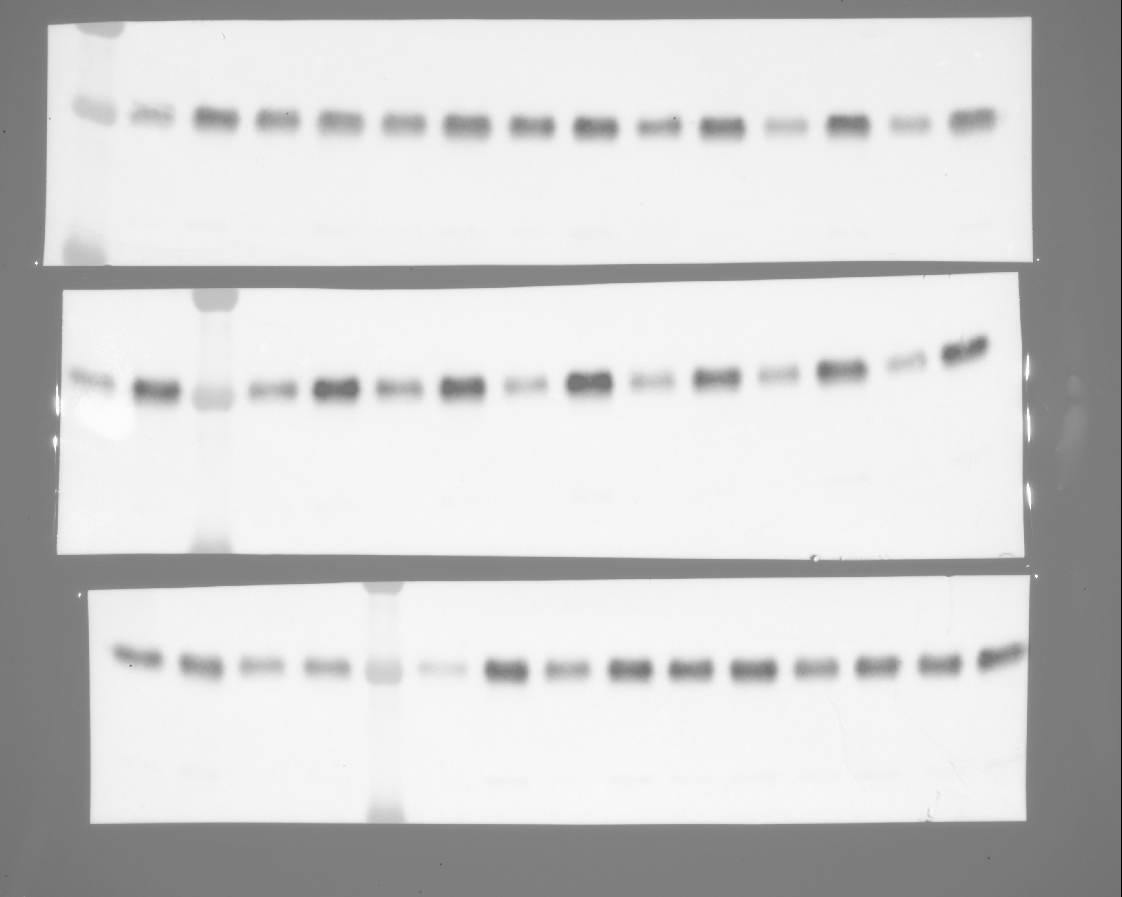

Supplement: Figure 1—figure supplement 1—source data 3. [file elife-103620-fig1-figsupp1-data3.zip › Figure 1-figure supplement 1-source data 3/CTXsyn WB Syn.tif]

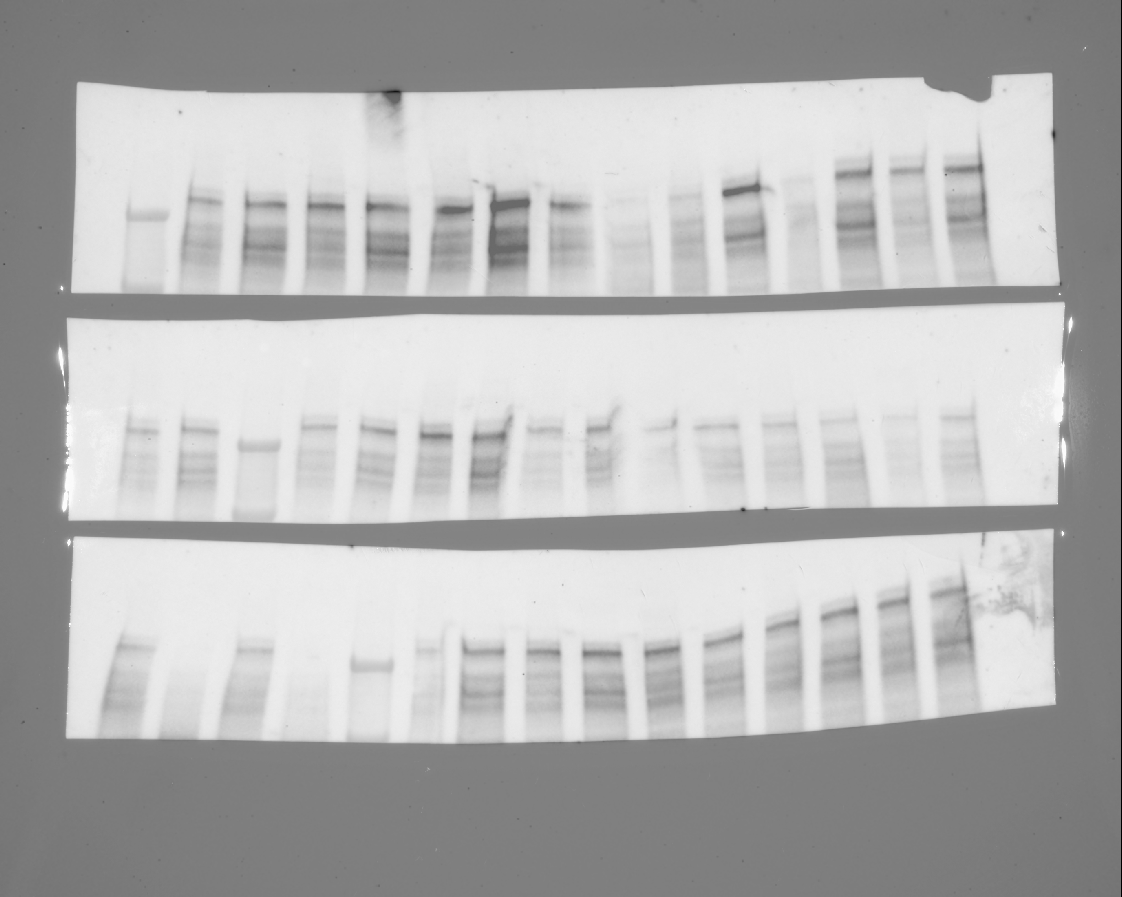

Supplement: Figure 1—figure supplement 1—source data 3. [file elife-103620-fig1-figsupp1-data3.zip › Figure 1-figure supplement 1-source data 3/CTXsyn WB TrioSR56.tif]

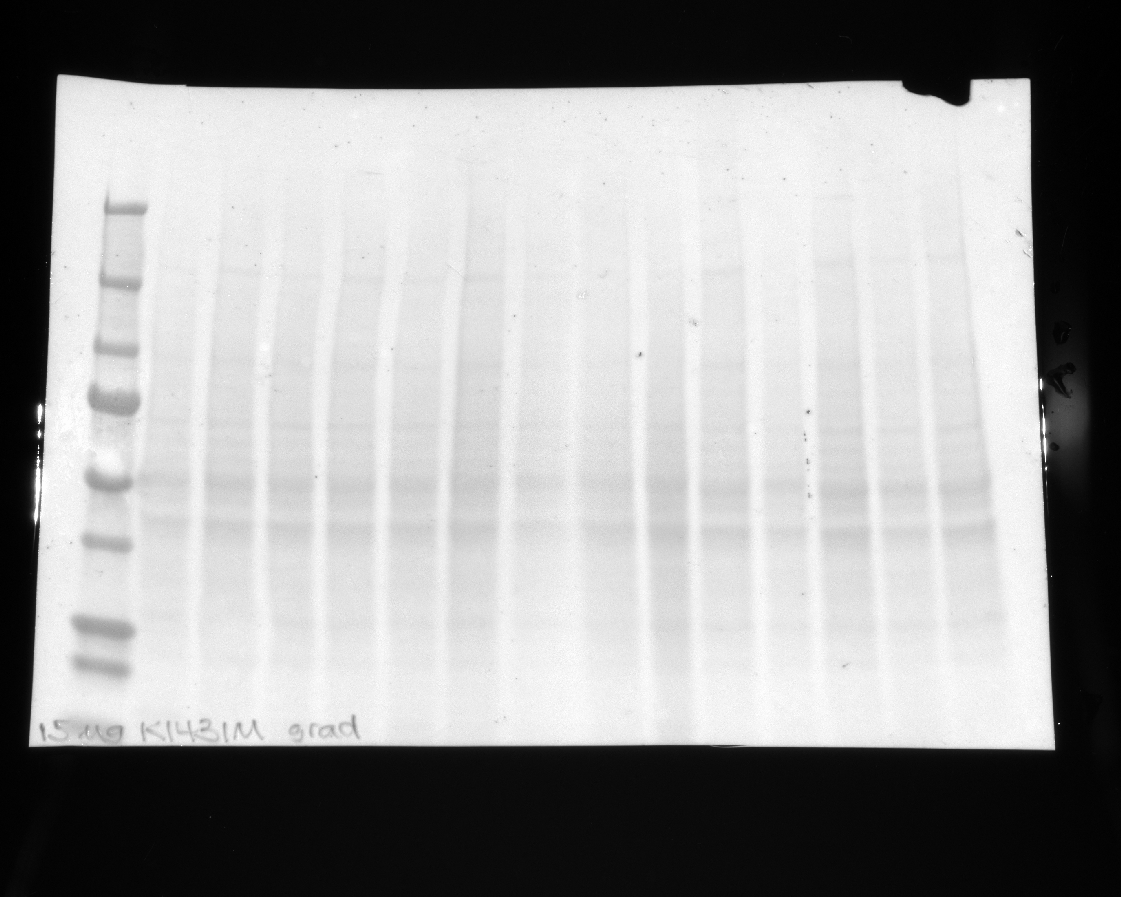

Supplement: Figure 1—figure supplement 1—source data 3. [file elife-103620-fig1-figsupp1-data3.zip › Figure 1-figure supplement 1-source data 3/K1431M CTXsyn Ponceau.tif]

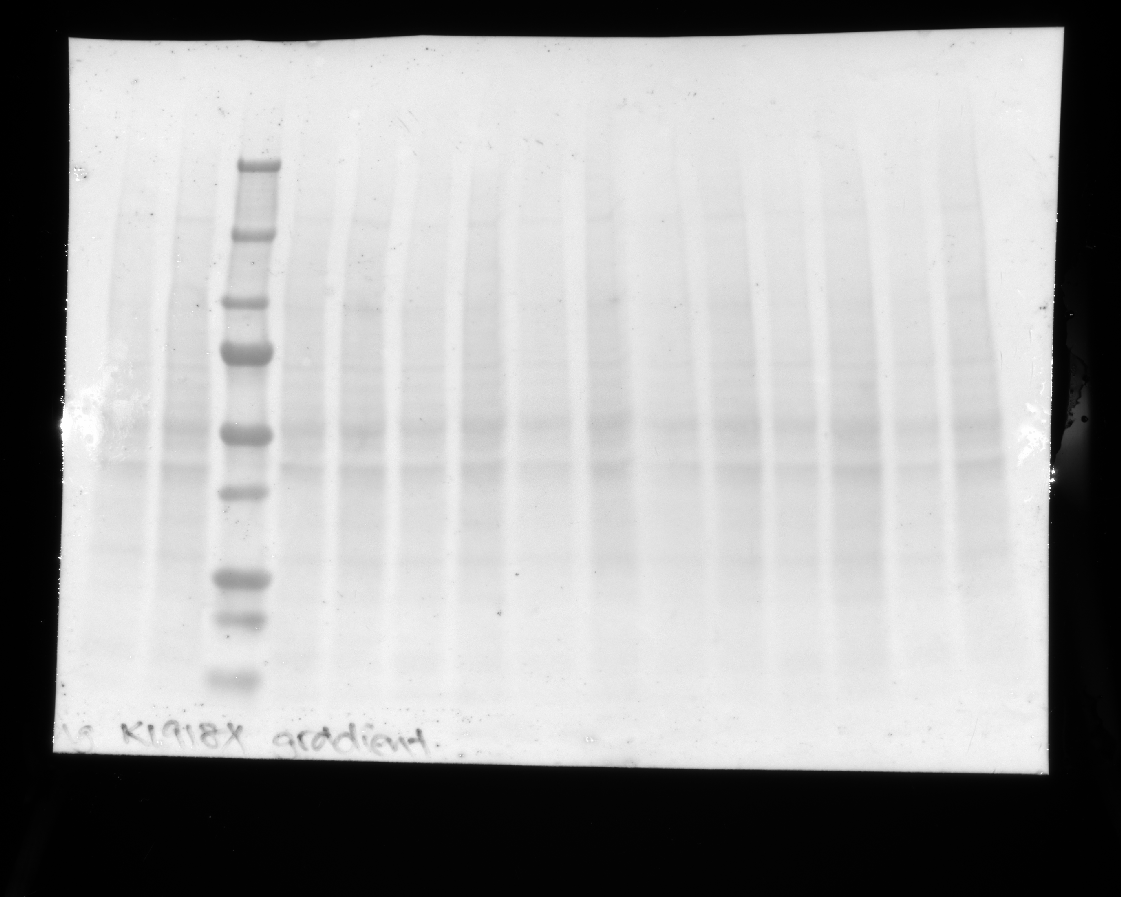

Supplement: Figure 1—figure supplement 1—source data 3. [file elife-103620-fig1-figsupp1-data3.zip › Figure 1-figure supplement 1-source data 3/K1918X CTXsyn Ponceau.tif]

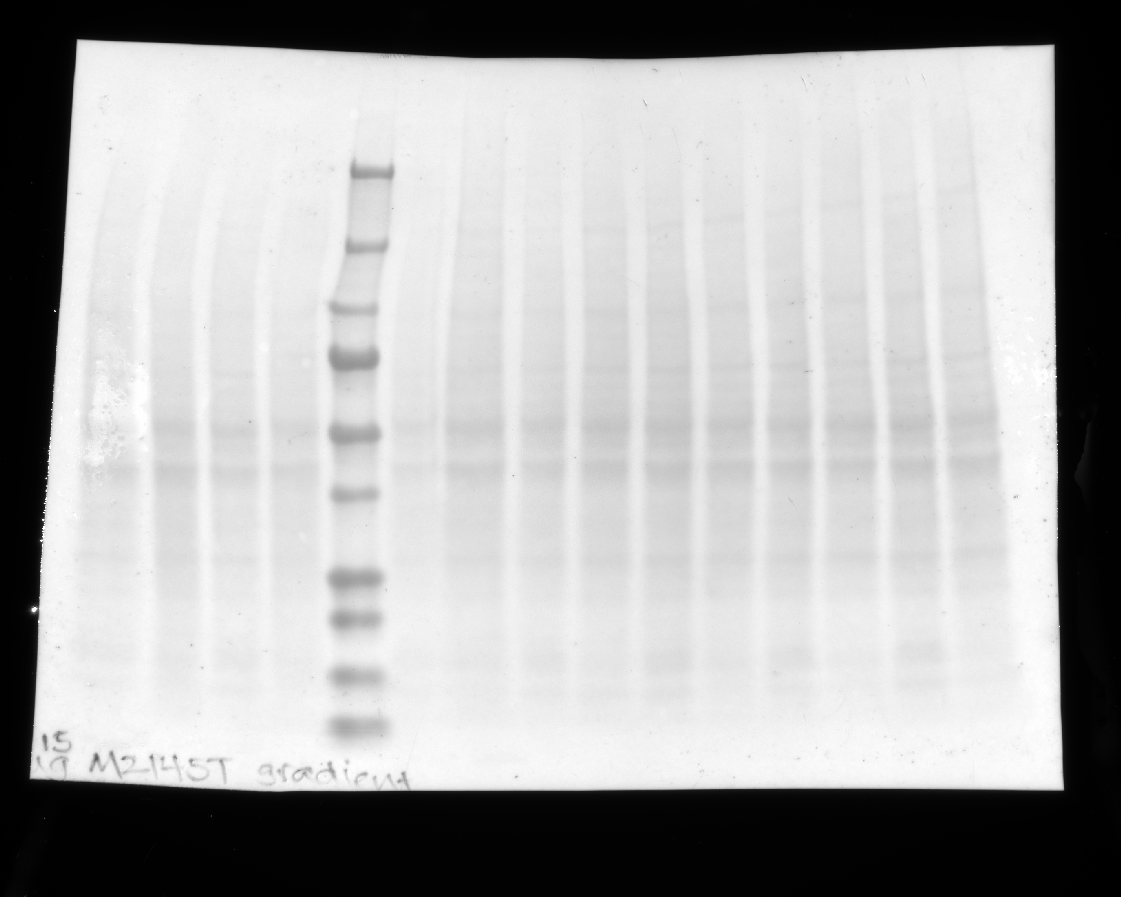

Supplement: Figure 1—figure supplement 1—source data 3. [file elife-103620-fig1-figsupp1-data3.zip › Figure 1-figure supplement 1-source data 3/M2145T CTXsyn Ponceau.tif]

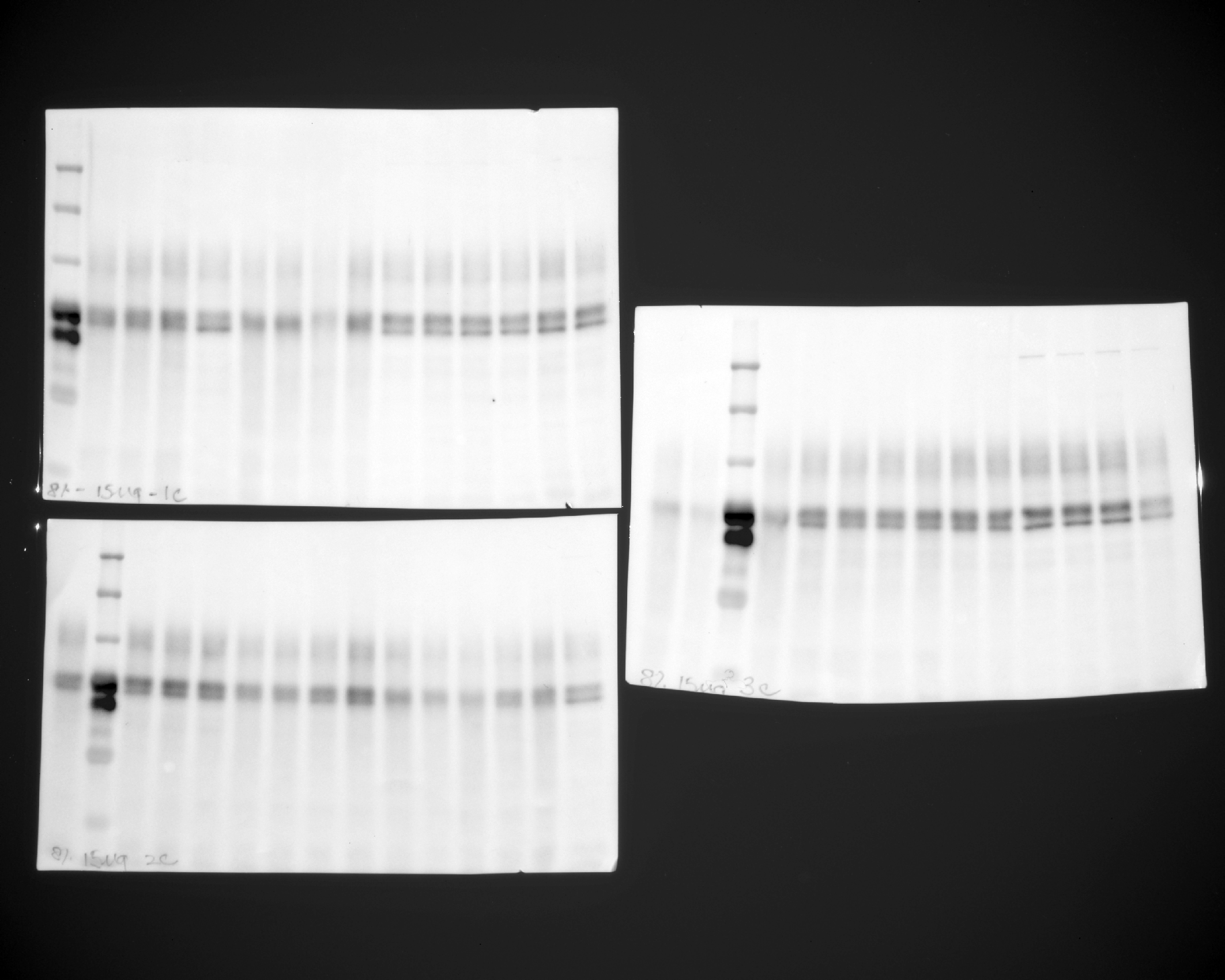

Supplement: Figure 7—source data 3. [file elife-103620-fig7-data3.zip › Figure 7-source data 3/CTXsyn 15ug WB Syt3.tif]

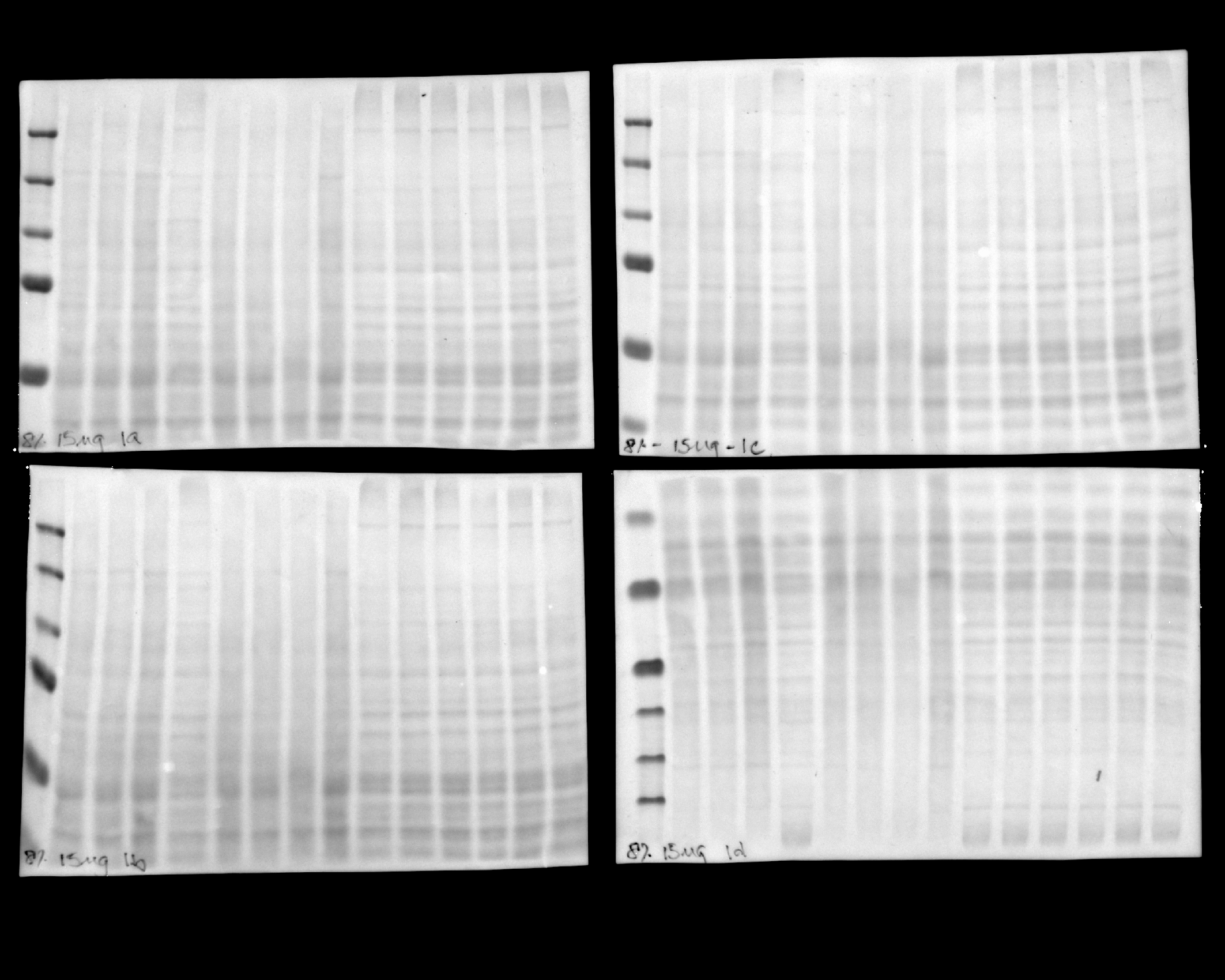

Supplement: Figure 7—source data 3. [file elife-103620-fig7-data3.zip › Figure 7-source data 3/CTXsyn 15ug-1 Ponceau.tif]

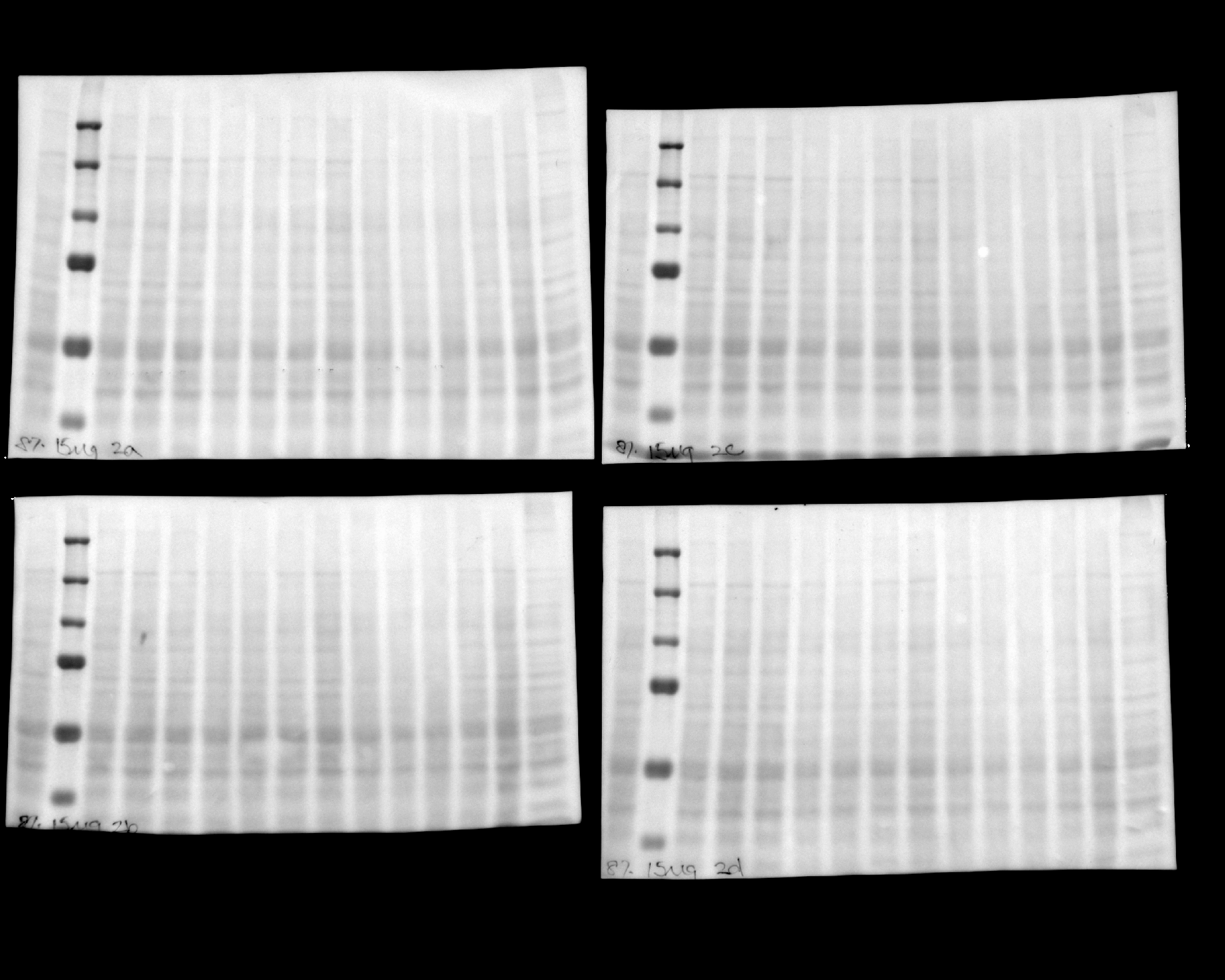

Supplement: Figure 7—source data 3. [file elife-103620-fig7-data3.zip › Figure 7-source data 3/CTXsyn 15ug-2 Ponceau.tif]

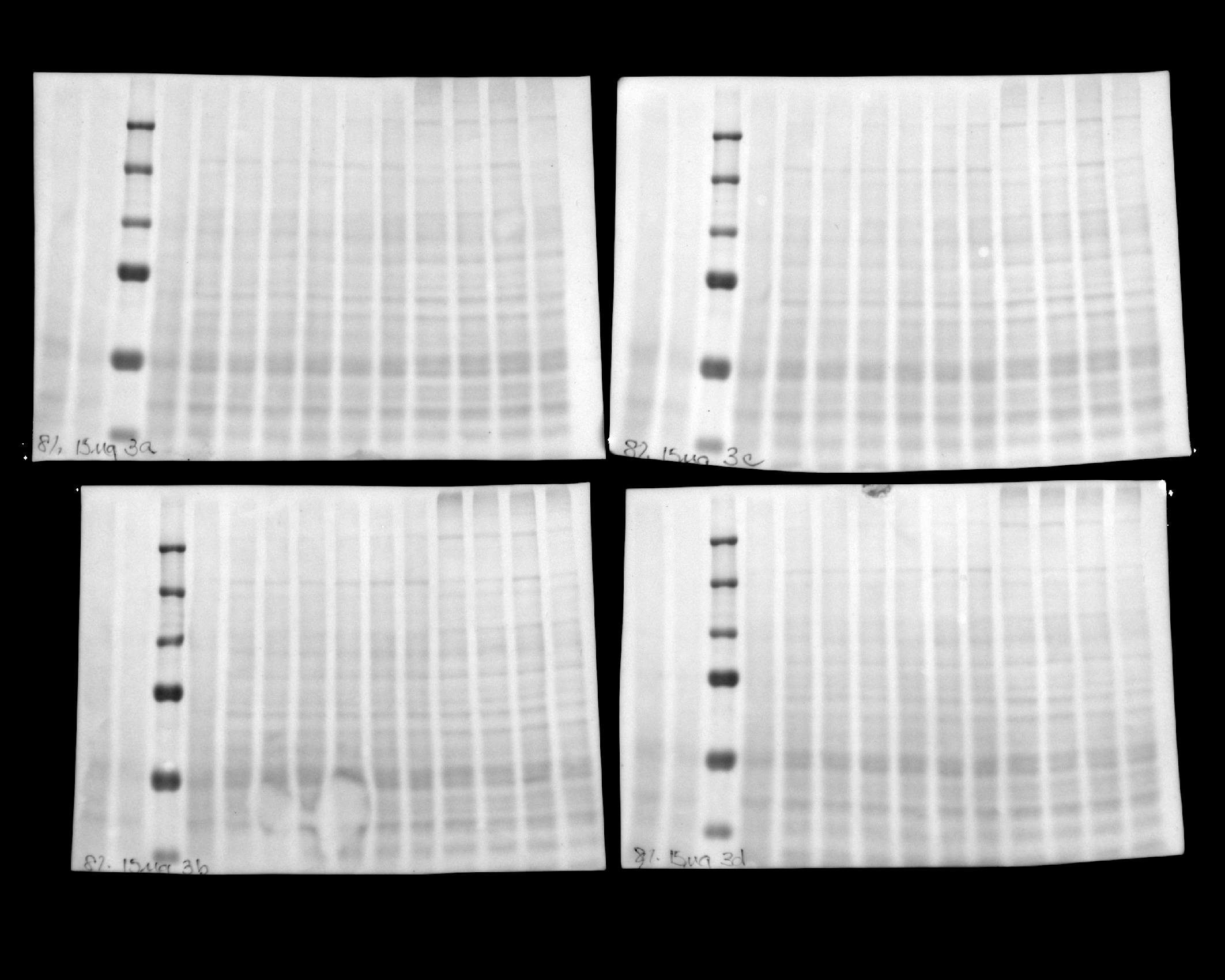

Supplement: Figure 7—source data 3. [file elife-103620-fig7-data3.zip › Figure 7-source data 3/CTXsyn 15ug-3 Ponceau.tif]

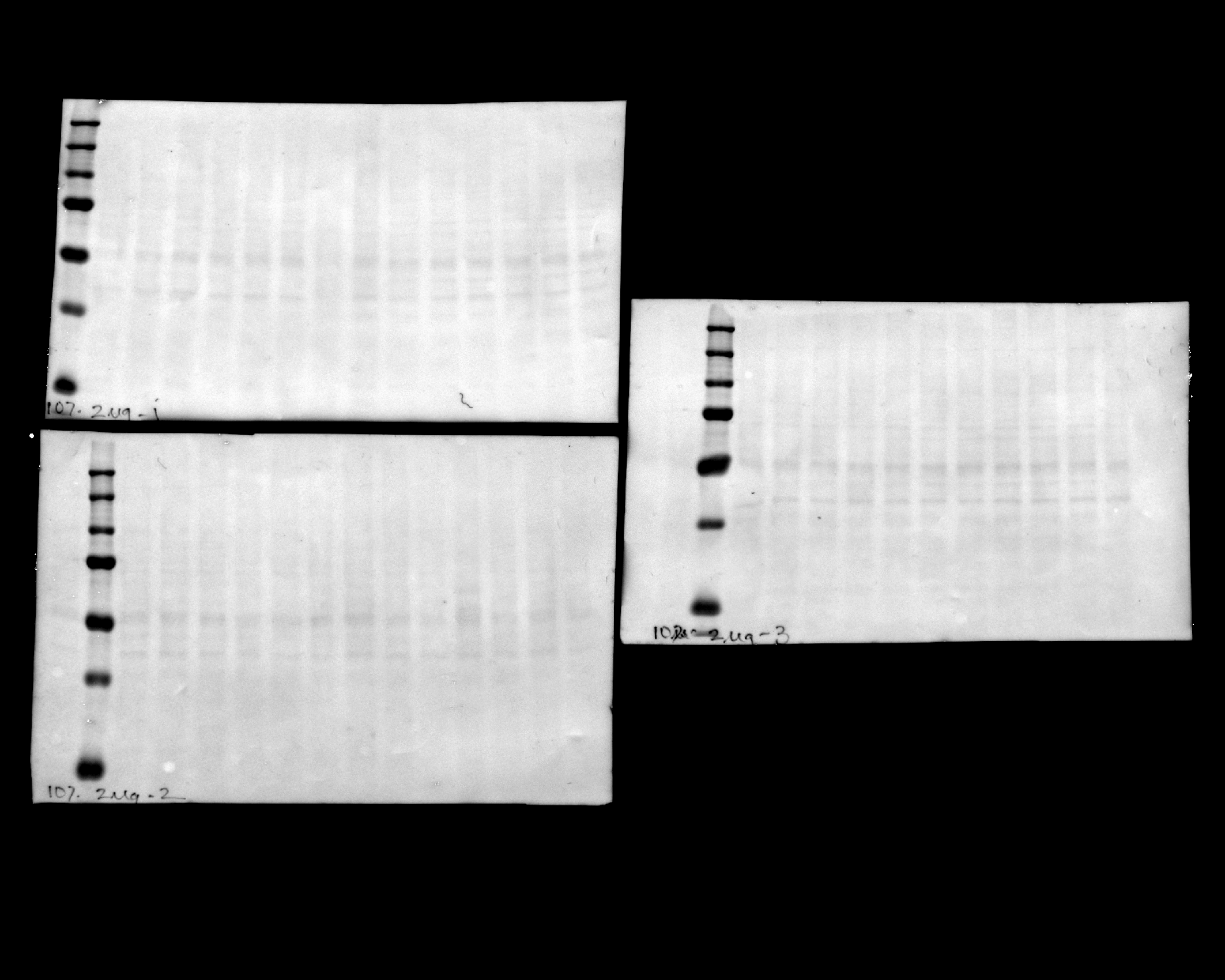

Supplement: Figure 7—source data 3. [file elife-103620-fig7-data3.zip › Figure 7-source data 3/CTXsyn 2ug Ponceau.tif]

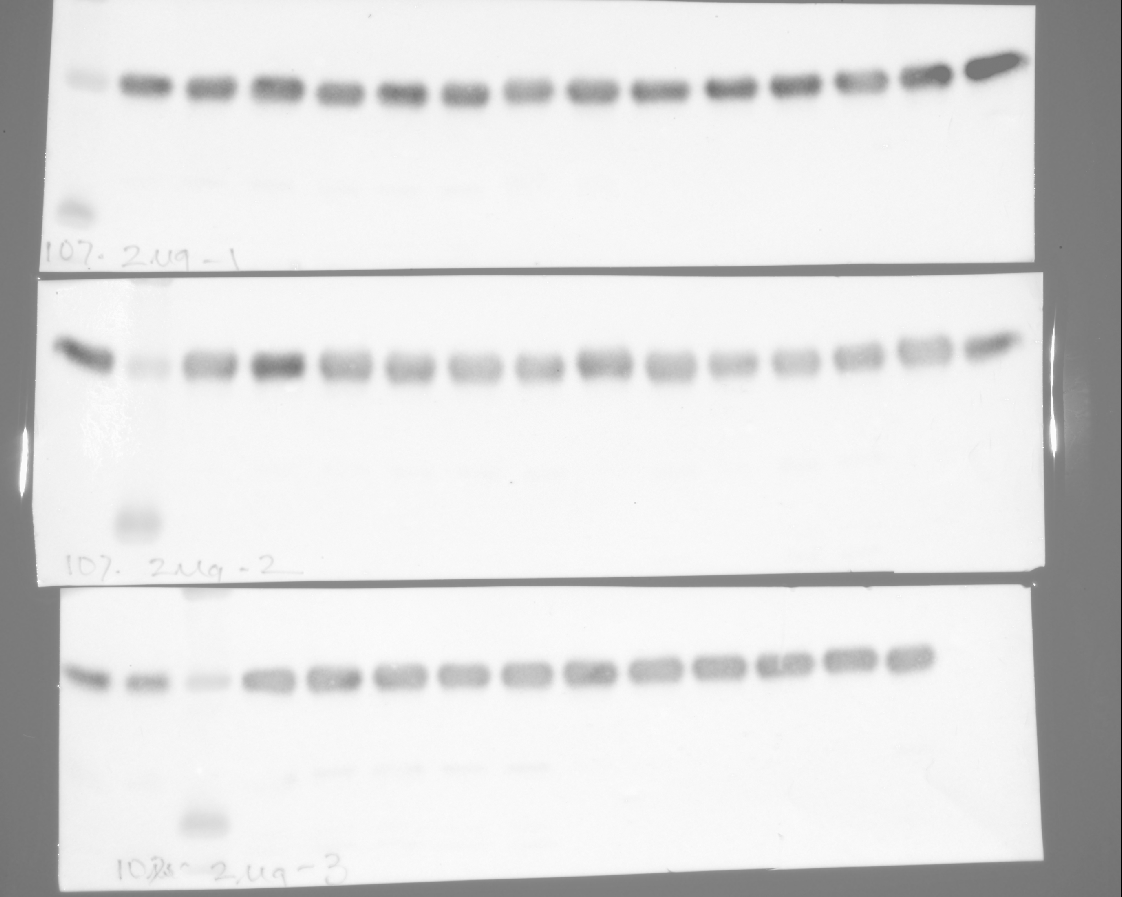

Supplement: Figure 7—source data 3. [file elife-103620-fig7-data3.zip › Figure 7-source data 3/CTXsyn 2ug WB Syp.tif]

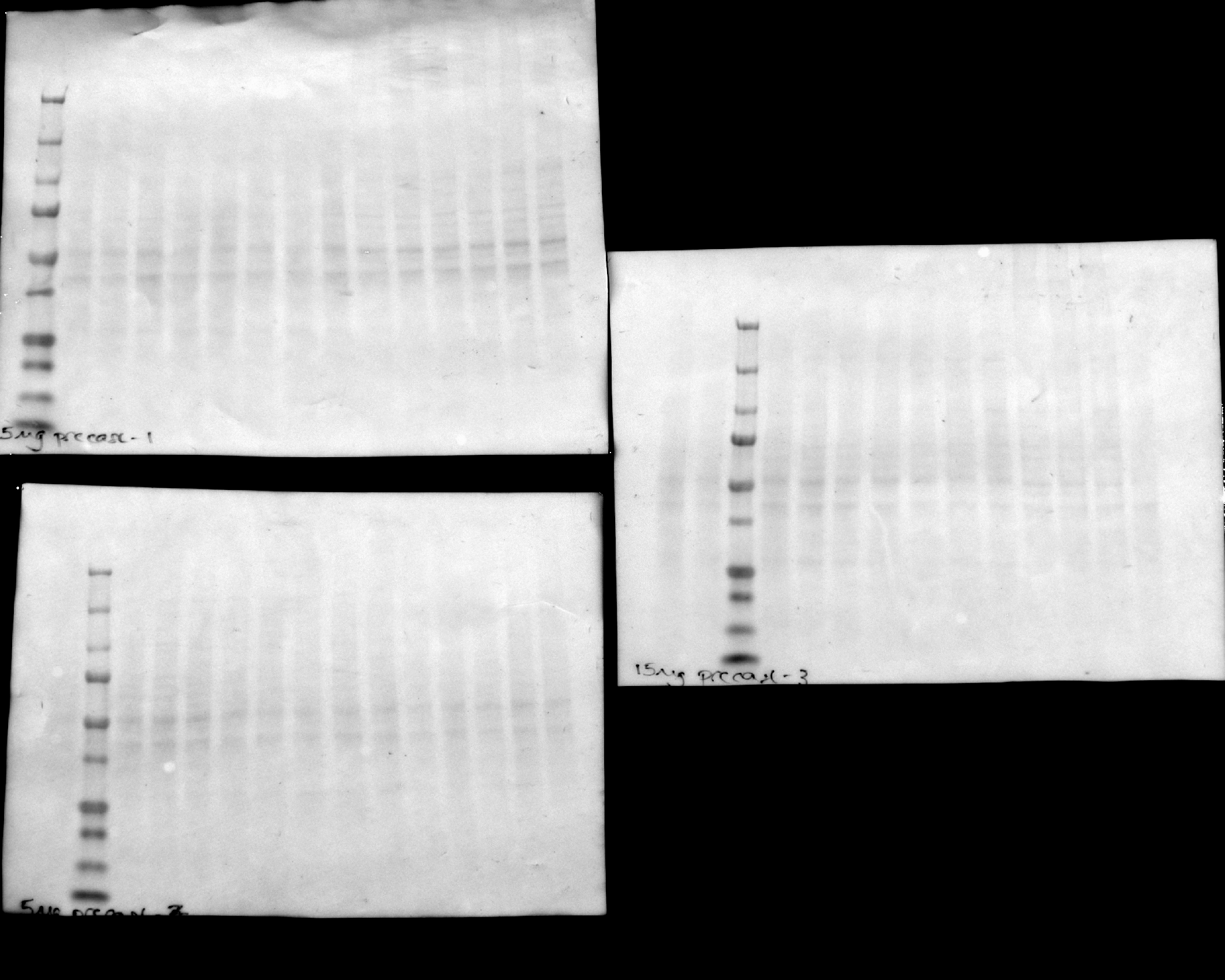

Supplement: Figure 7—source data 3. [file elife-103620-fig7-data3.zip › Figure 7-source data 3/CTXsyn 5ug Ponceau.tif]

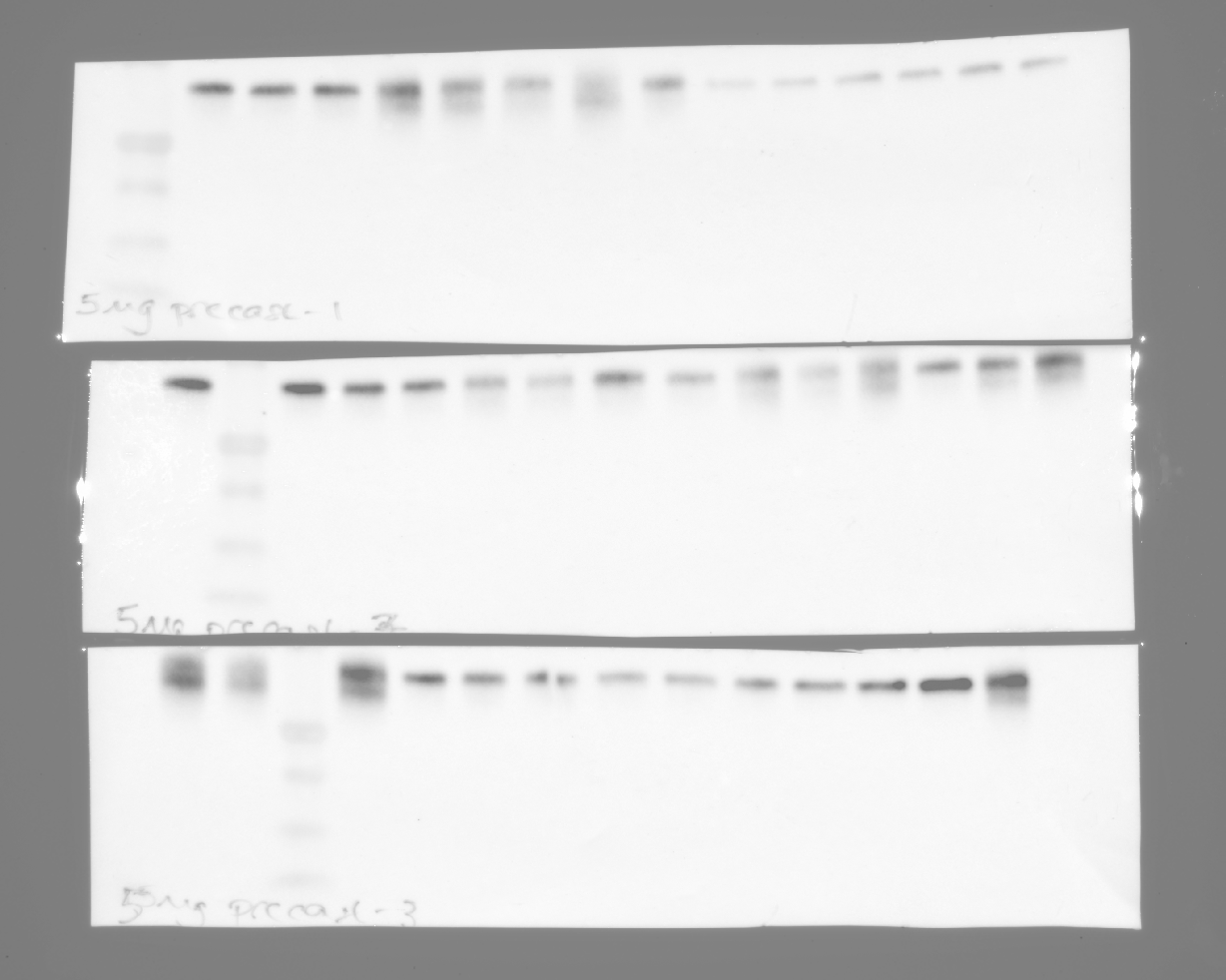

Supplement: Figure 7—source data 3. [file elife-103620-fig7-data3.zip › Figure 7-source data 3/CTXsyn 5ug WB STX1a.tif]

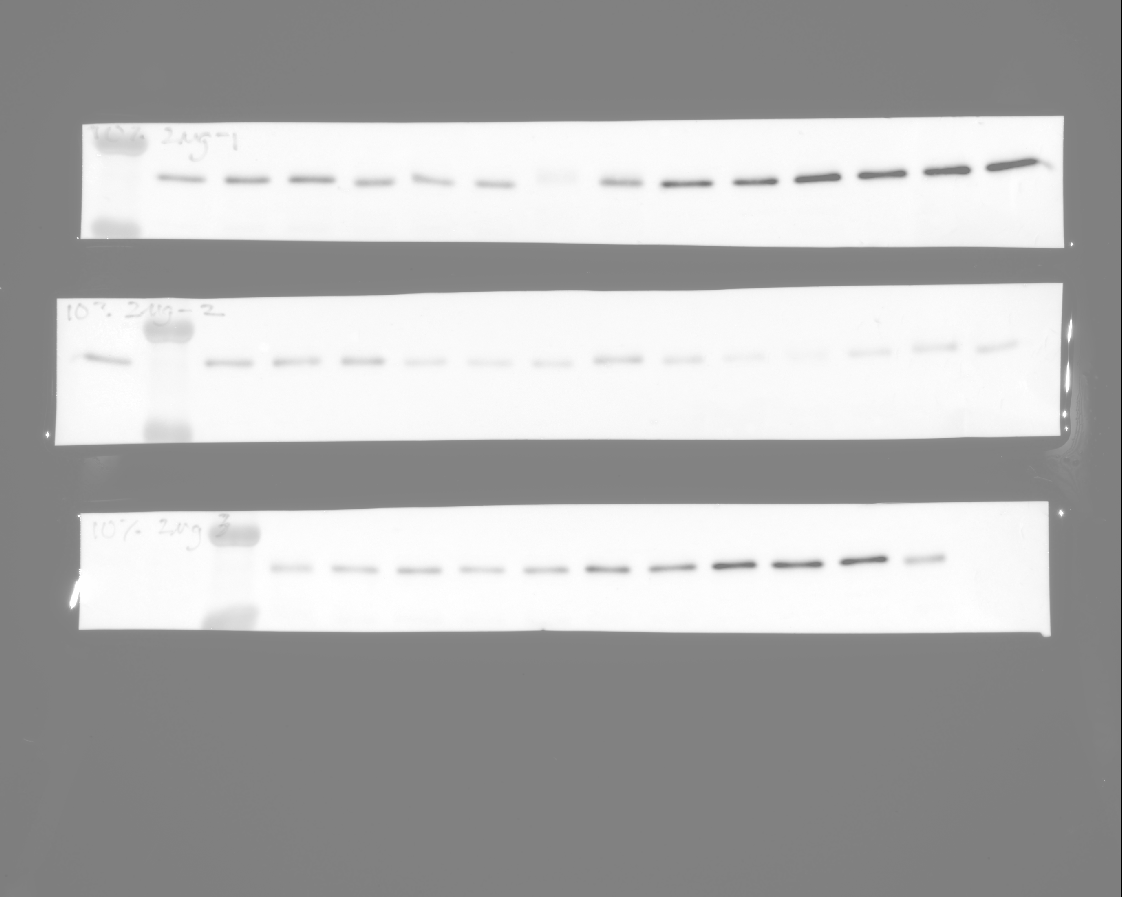

Supplement: Figure 7—source data 3. [file elife-103620-fig7-data3.zip › Figure 7-source data 3/K1431M CTXsyn 2ug WB Munc18.tif]

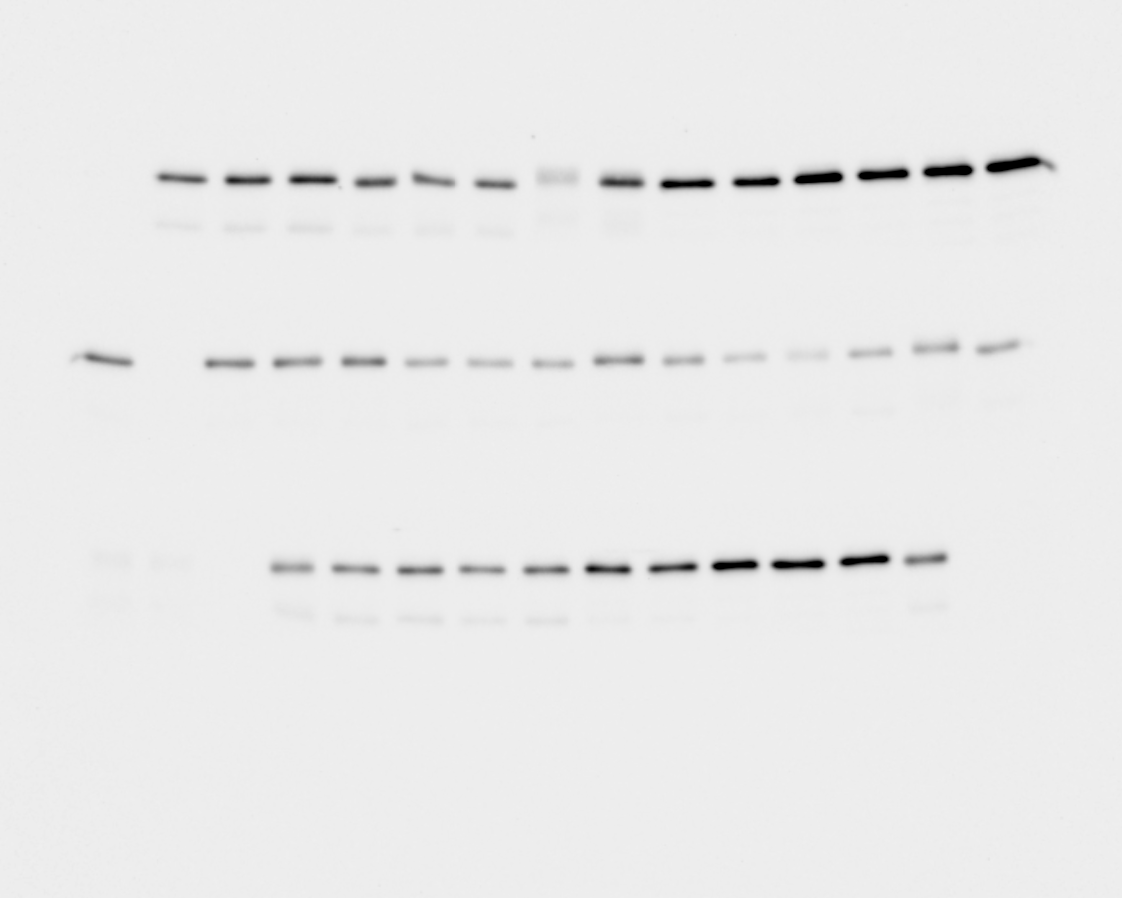

Supplement: Figure 7—source data 3. [file elife-103620-fig7-data3.zip › Figure 7-source data 3/K1918X CTXsyn 2ug WB Munc18.tif]

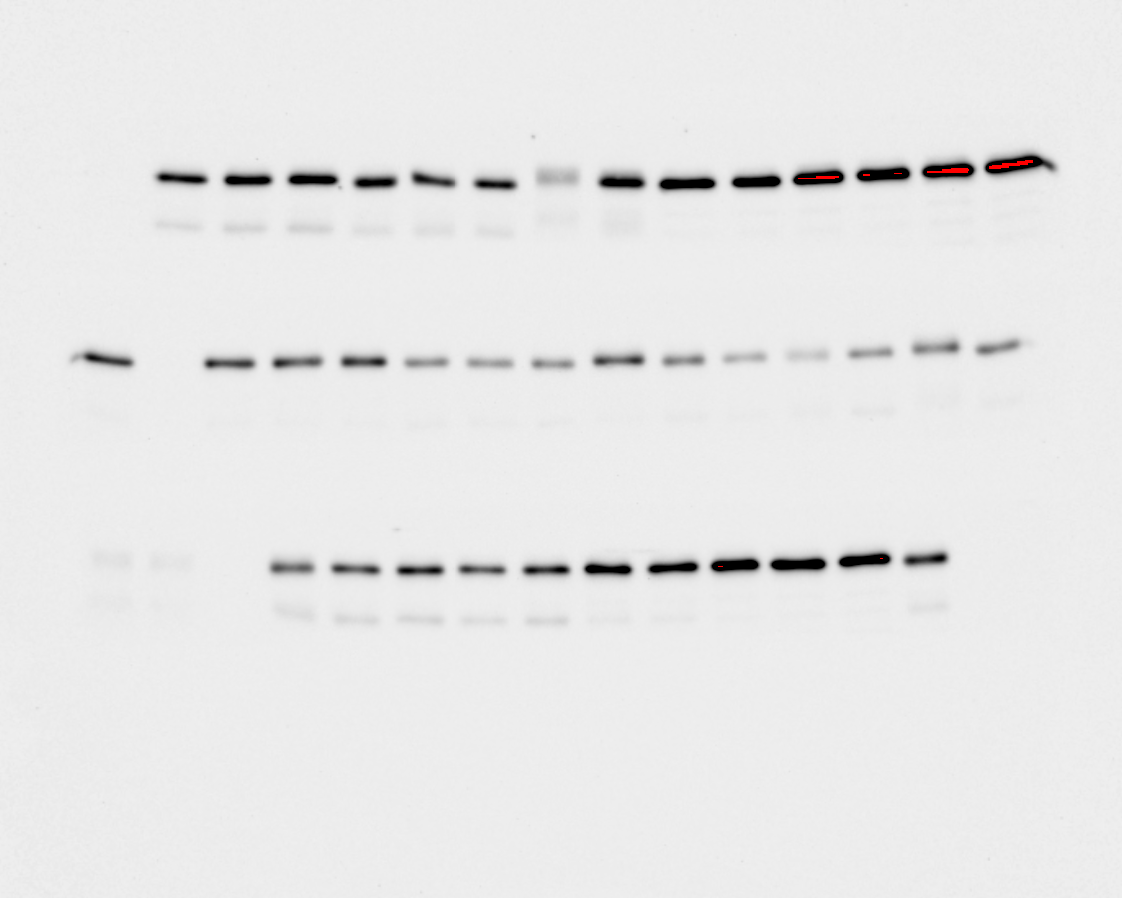

Supplement: Figure 7—source data 3. [file elife-103620-fig7-data3.zip › Figure 7-source data 3/M2145T CTXsyn 2ug WB Munc18.tif]

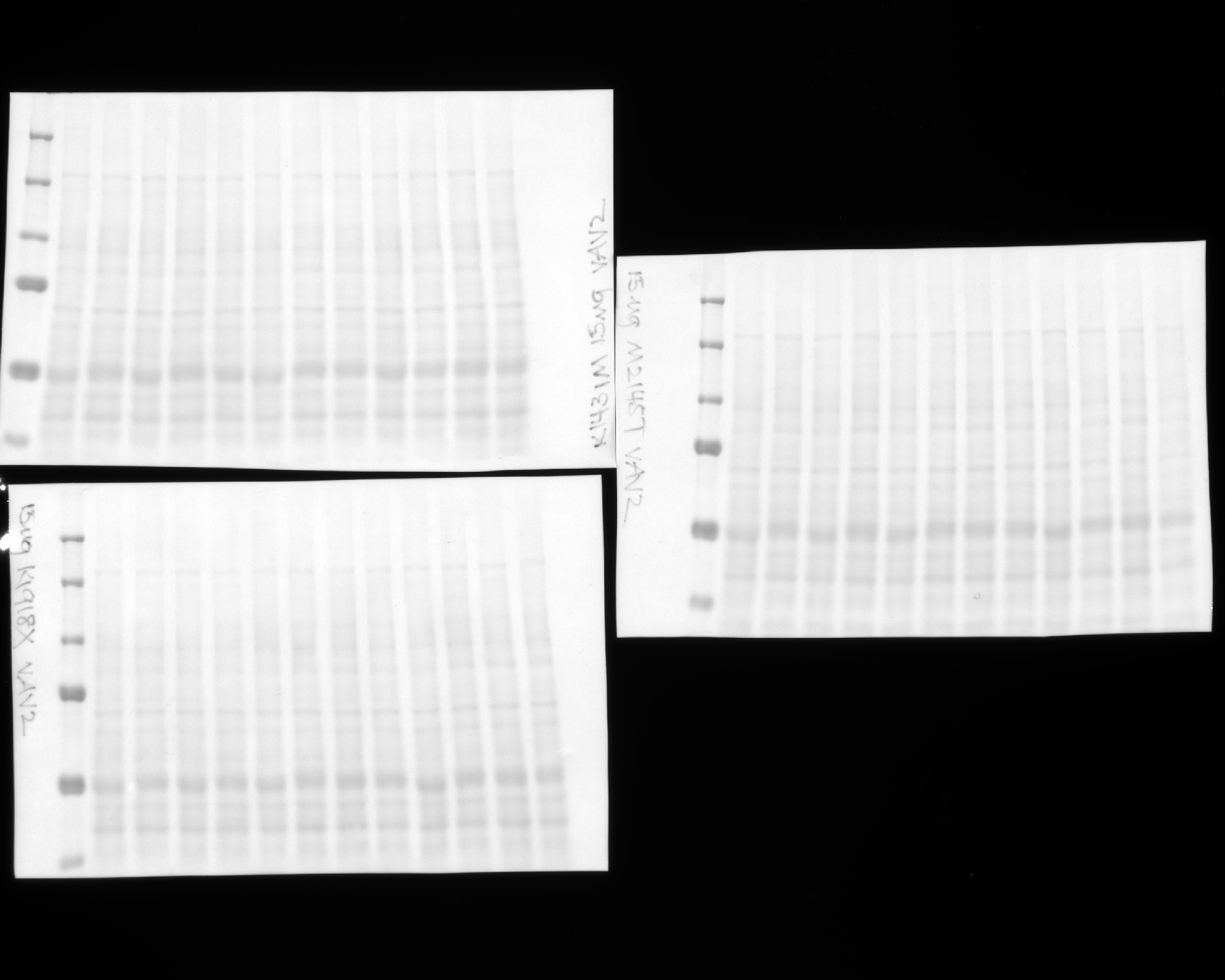

Supplement: Figure 7—source data 5. [file elife-103620-fig7-data5.zip › Folder 7-source data 5/CTX1 Ponceau.tif]

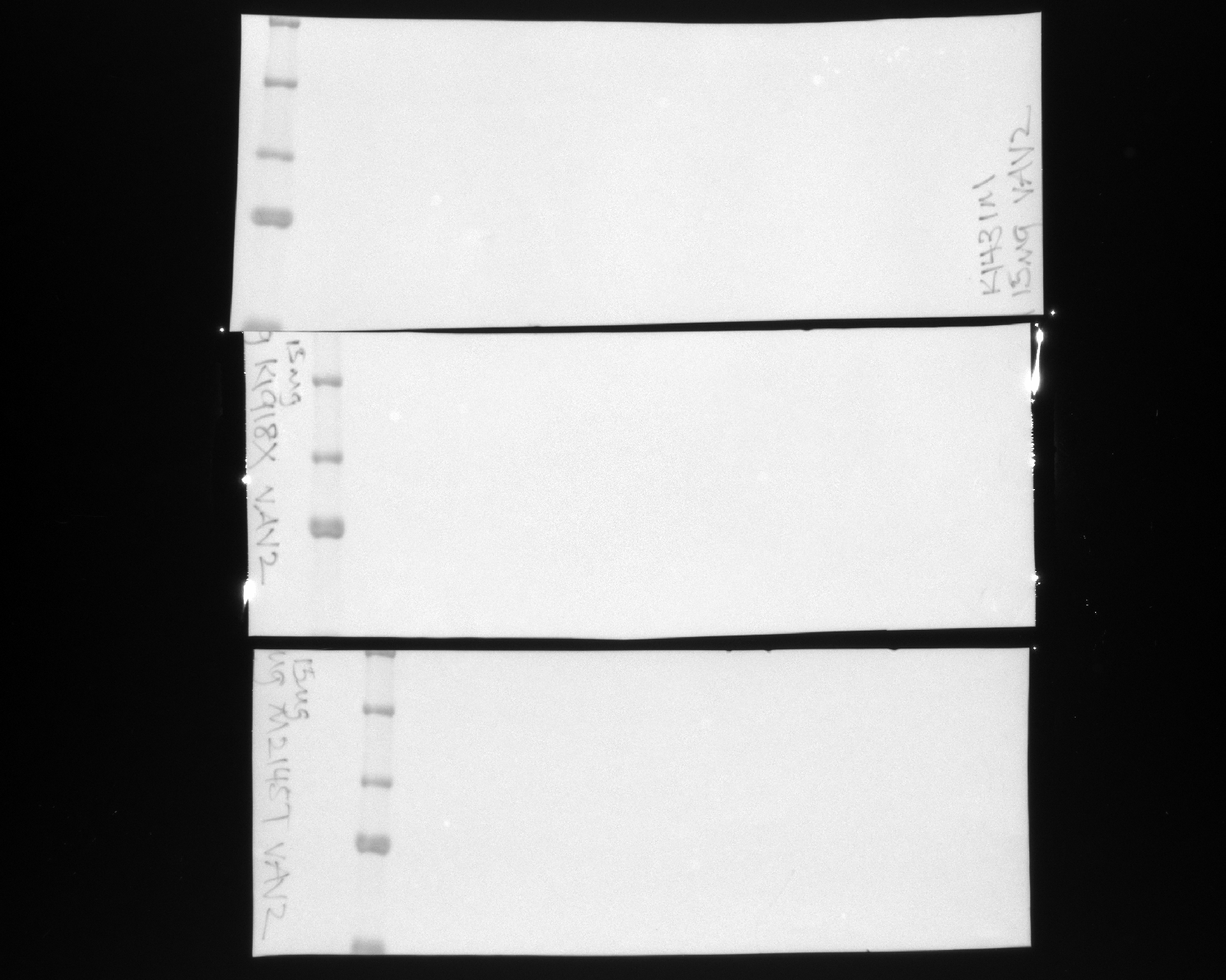

Supplement: Figure 7—source data 5. [file elife-103620-fig7-data5.zip › Folder 7-source data 5/CTX1 WB Vav2(Colorimetric).tif]

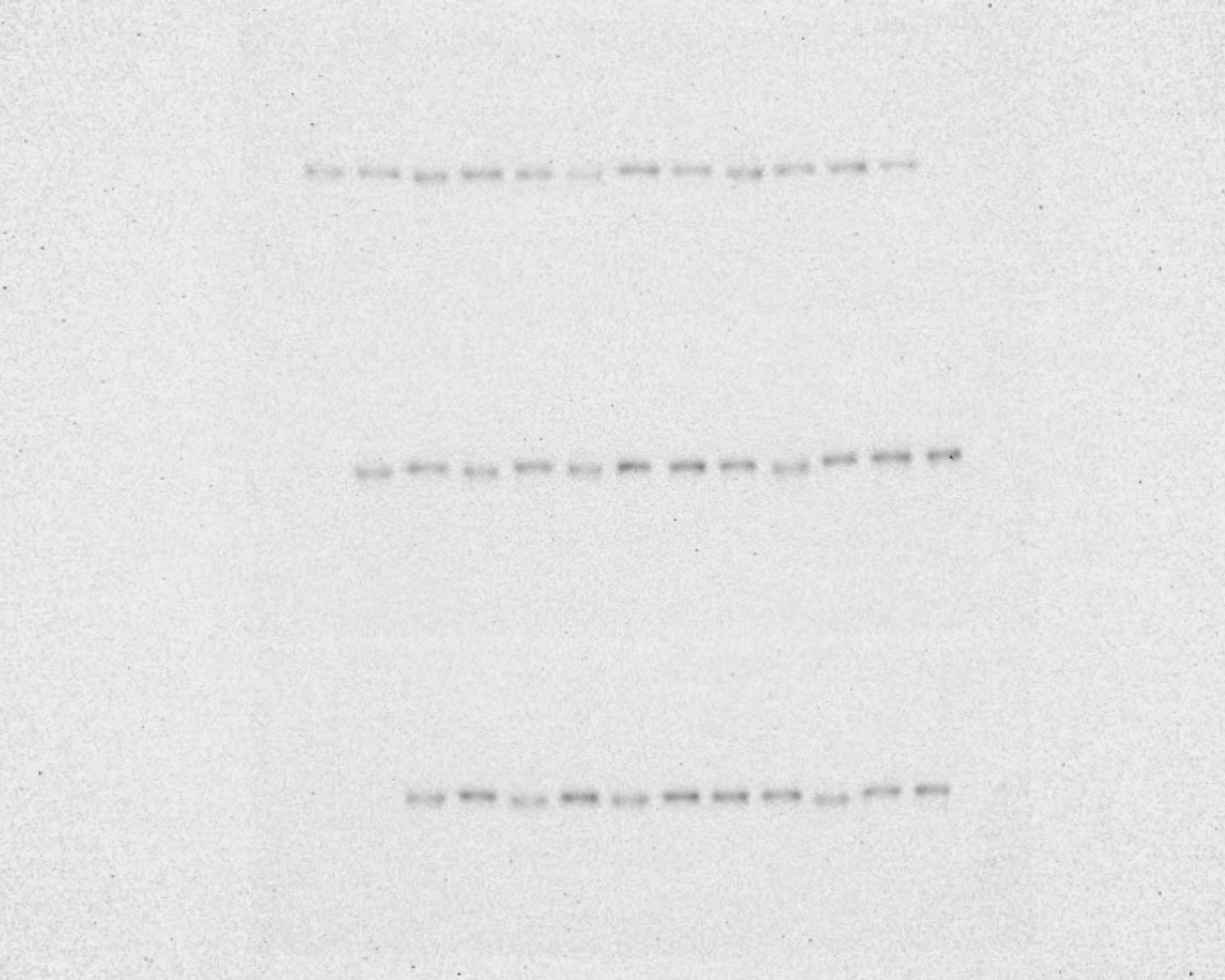

Supplement: Figure 7—source data 5. [file elife-103620-fig7-data5.zip › Folder 7-source data 5/CTX1 WB Vav2.tif]

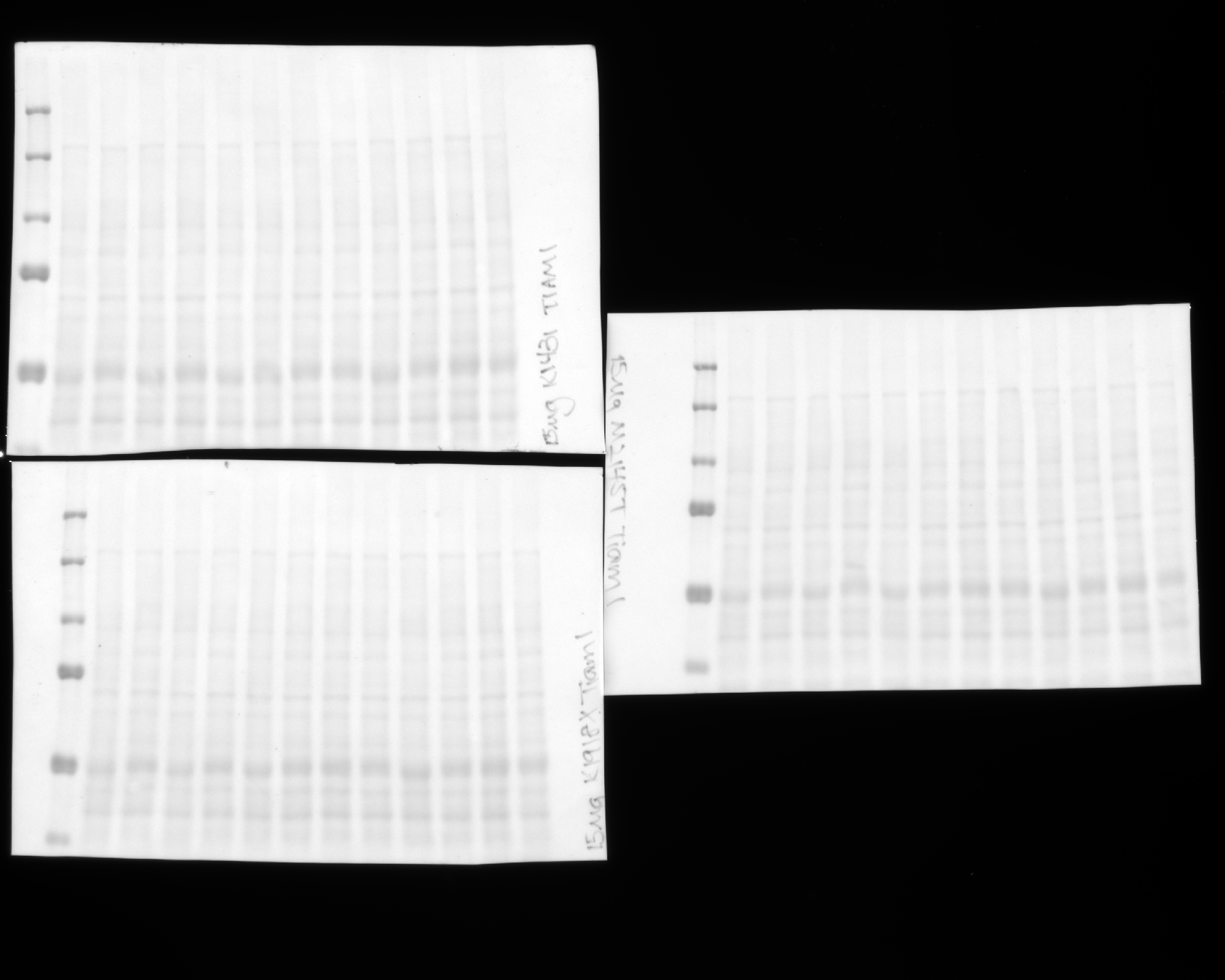

Supplement: Figure 7—source data 5. [file elife-103620-fig7-data5.zip › Folder 7-source data 5/CTX2 Ponceau.tif]

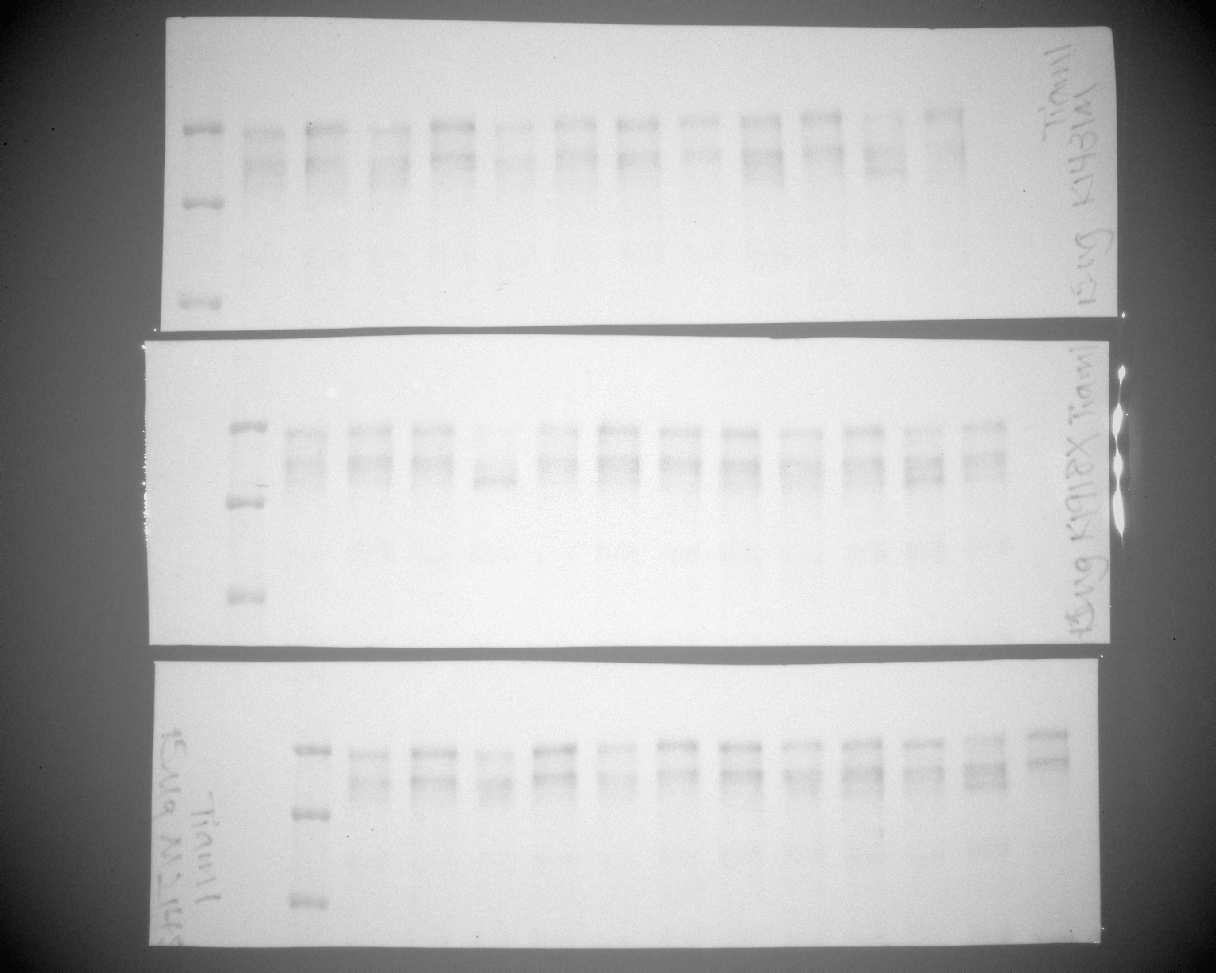

Supplement: Figure 7—source data 5. [file elife-103620-fig7-data5.zip › Folder 7-source data 5/CTX2 WB Tiam1.tif]

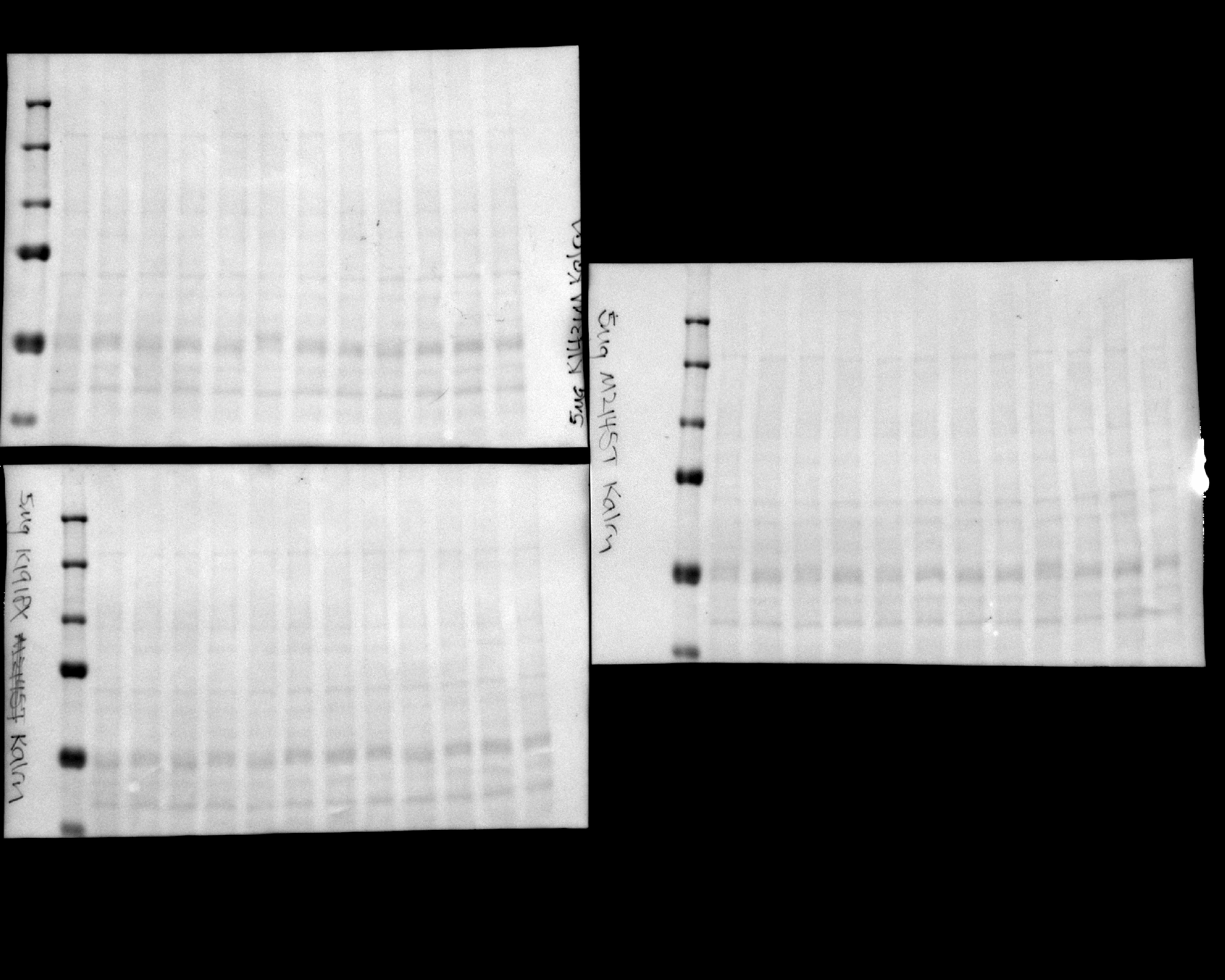

Supplement: Figure 7—source data 5. [file elife-103620-fig7-data5.zip › Folder 7-source data 5/CTX3 Ponceau.tif]

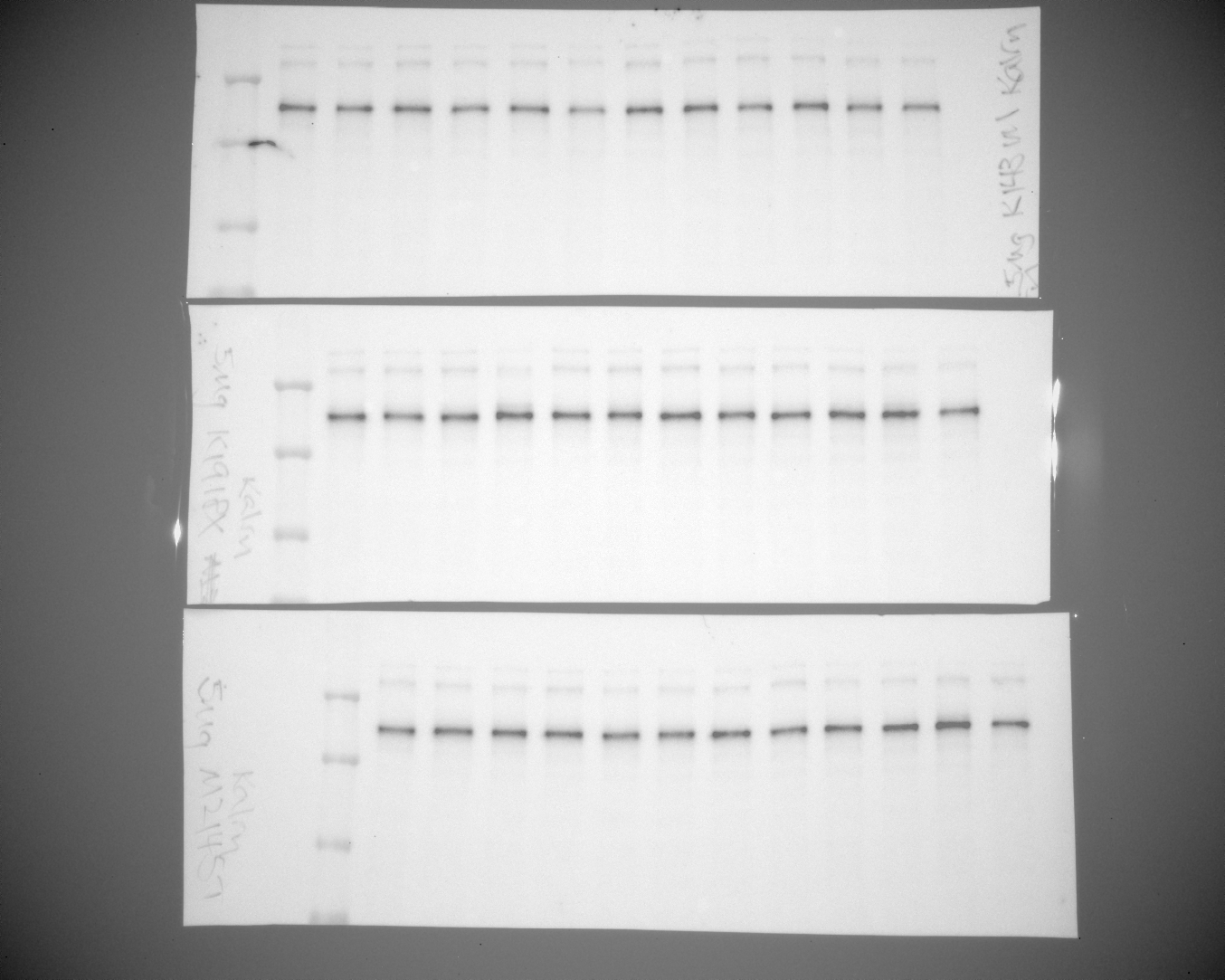

Supplement: Figure 7—source data 5. [file elife-103620-fig7-data5.zip › Folder 7-source data 5/CTX3 WB Kalrn.tif]
