# Supplementary material for: Heterozygosity for neurodevelopmental disorder-associated TRIO variants yields distinct deficits in behavior, neuronal development, and synaptic transmission in mice
Source: eLife. 2025 Jun 9;13:RP103620. doi: 10.7554/eLife.103620 (PMC12148328; doi:10.7554/eLife.103620)
Supplement: Figure 1—source data 2. [file elife-103620-fig1-data2.zip › Figure 1-source data 2/Figure 1-source data 2.pdf]

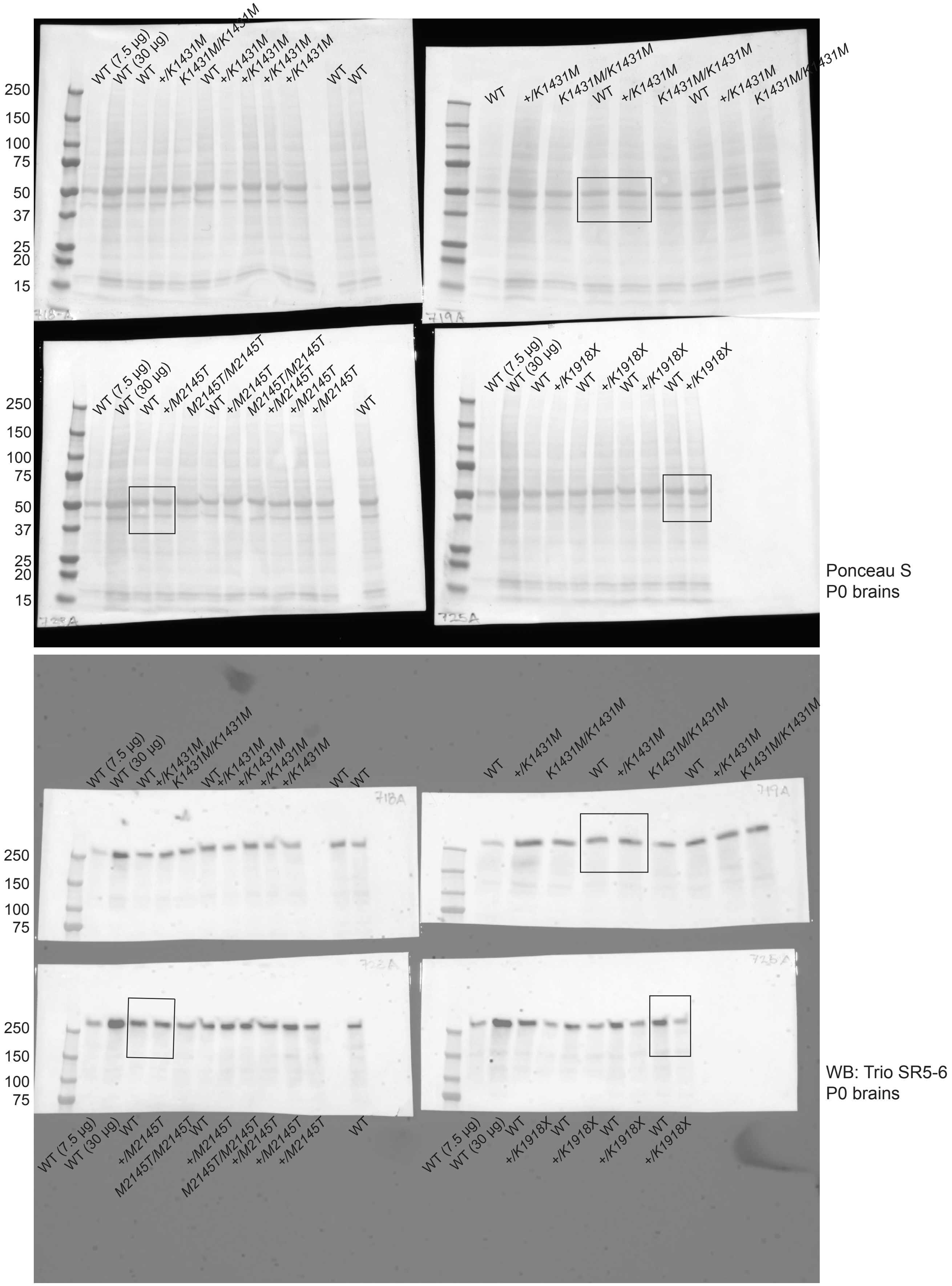

Figure 1-source data 2. Original membranes corresponding to Figure 1, panel C.
15 µg brain lysate from P0 littermate pups were separated by gel electrophoresis and stained by Ponceau S (top), and blotted for Trio with an anti-Trio SR5-6 antibody (bottom). 7.5 µg and 30 µg WT brain lysate were included as internal controls. Boxes indicates cropped images used in final figure.
