# Supplementary material for: Heterozygosity for neurodevelopmental disorder-associated TRIO variants yields distinct deficits in behavior, neuronal development, and synaptic transmission in mice
Source: eLife. 2025 Jun 9;13:RP103620. doi: 10.7554/eLife.103620 (PMC12148328; doi:10.7554/eLife.103620)
Supplement: Figure 1—figure supplement 1—source data 2. [file elife-103620-fig1-figsupp1-data2.zip › Figure 1-figure supplement 1-source data 2/Figure 1-figure supplement 1-source data 2.pdf]

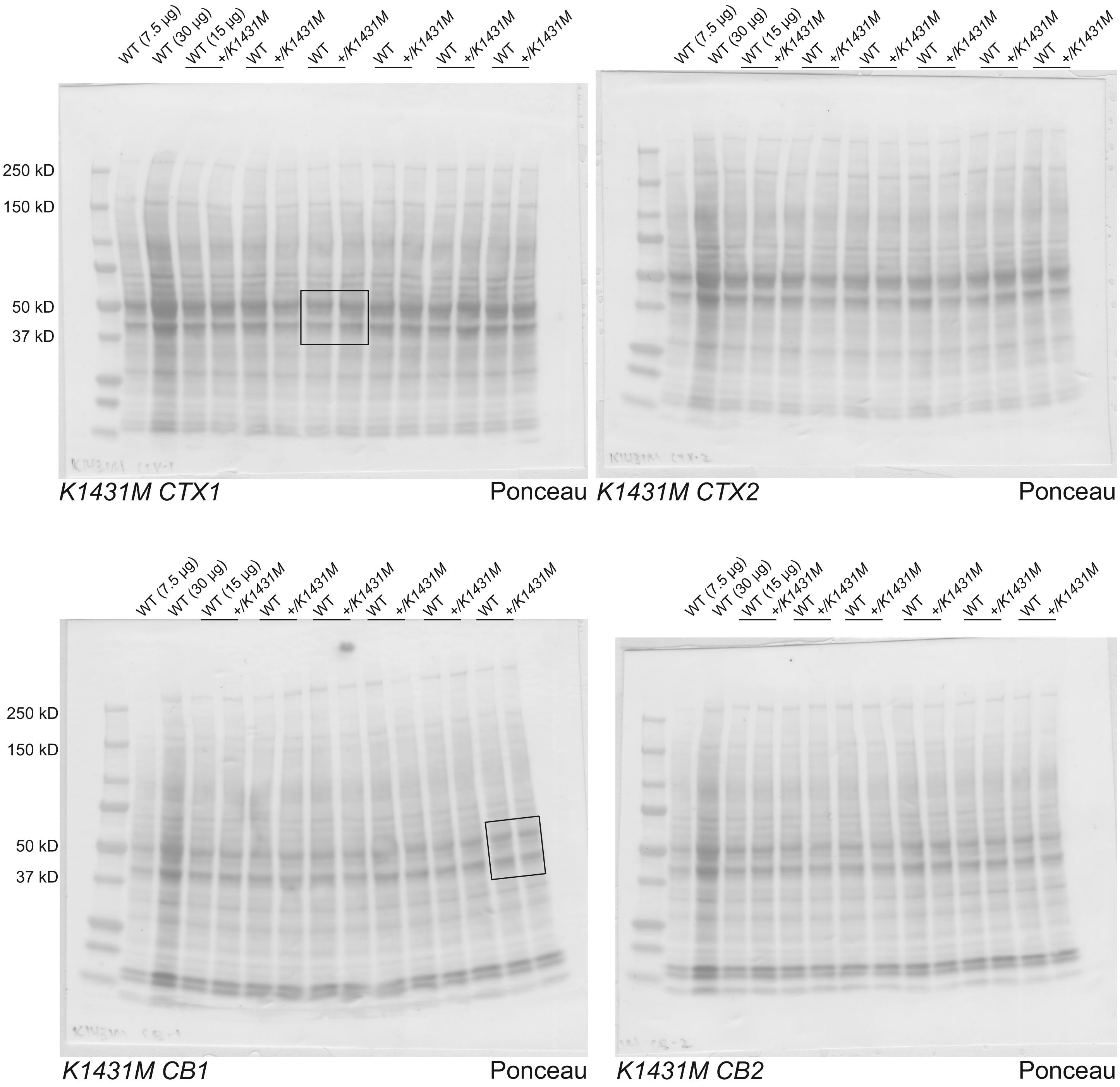

Figure 1-figure supplement 1-source data 2. Original membranes corresponding to Figure 1-figure supplement 1, panel D and panel H.

15 µg cortical (CTX) or cerebellar (CB) brain lysates from P42 paired littermate pups were separated by gel electrophoresis and stained by Ponceau S prior to blotting. 7.5 µg and 30 µg WT brain lysate were included as internal loading controls. Lines denote WT - *+/K1431M* littermate pairs. Boxes indicate cropped images used in final figure.

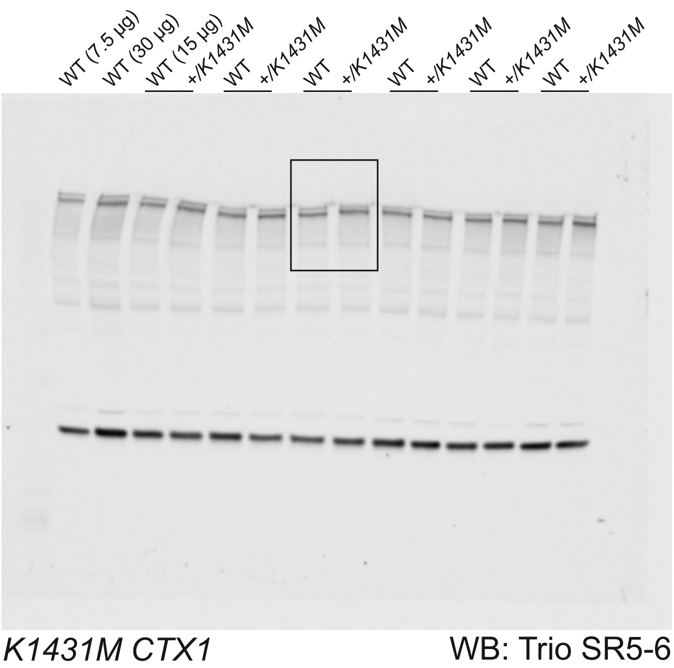

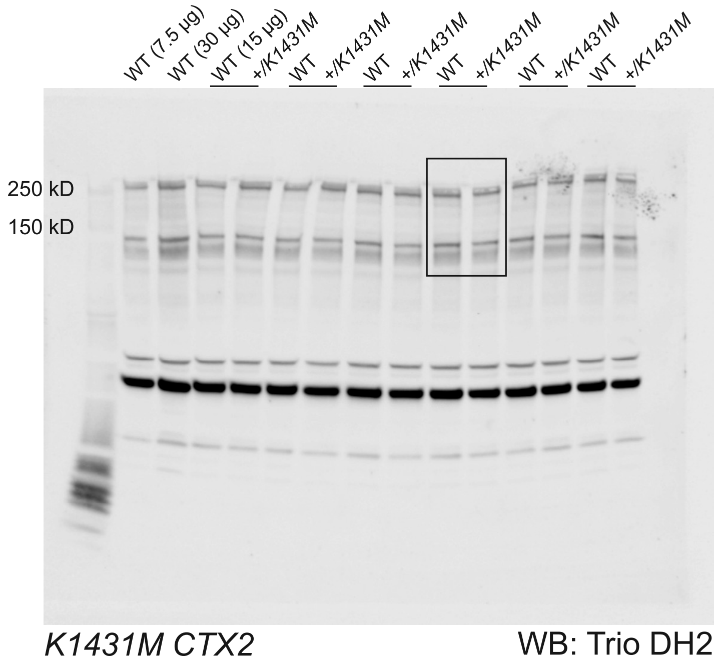

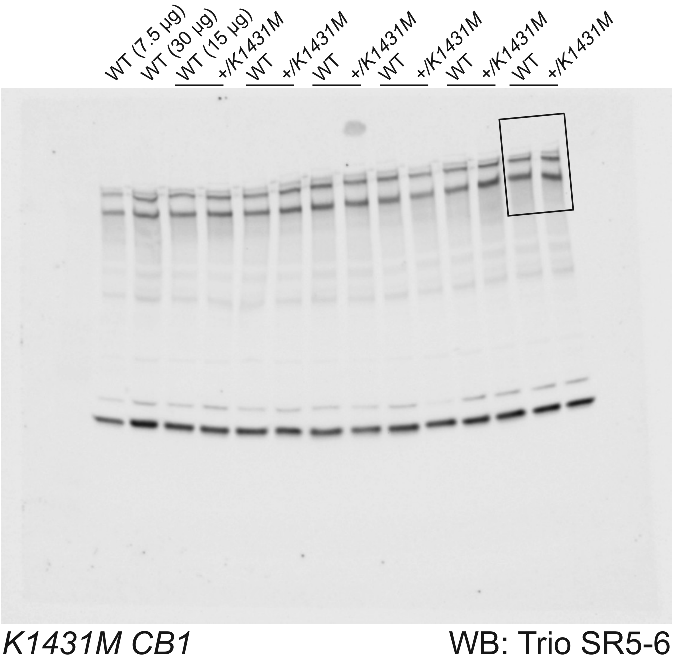

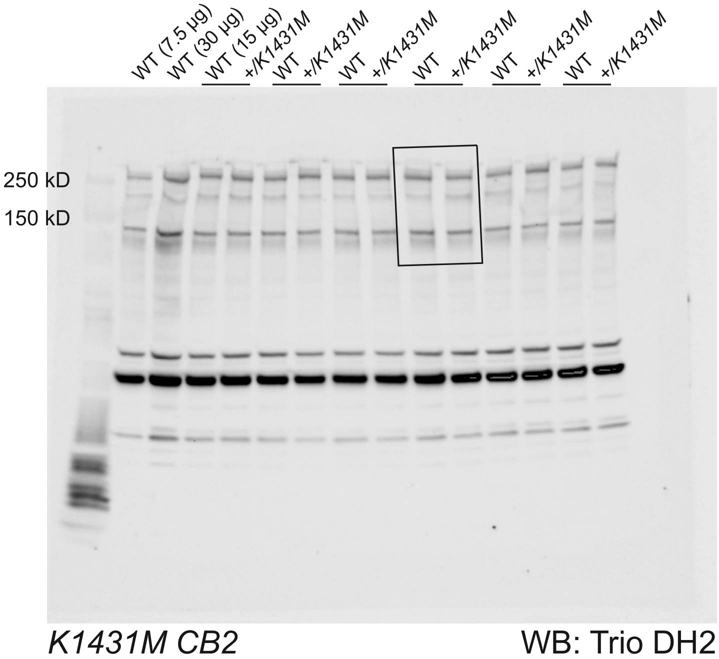

Membranes were blotted for Trio isoforms (Trio9S (263 kDa), Trio9L (277 kDa), Trio8 (217 kDa), Duet (145 kDa)) with an anti-Trio SR5-6 antibody (left) or anti-Trio DH2 antibody (right). Lines denote WT - *+/K1431M* littermate pairs. Boxes indicates cropped images used in final figure.

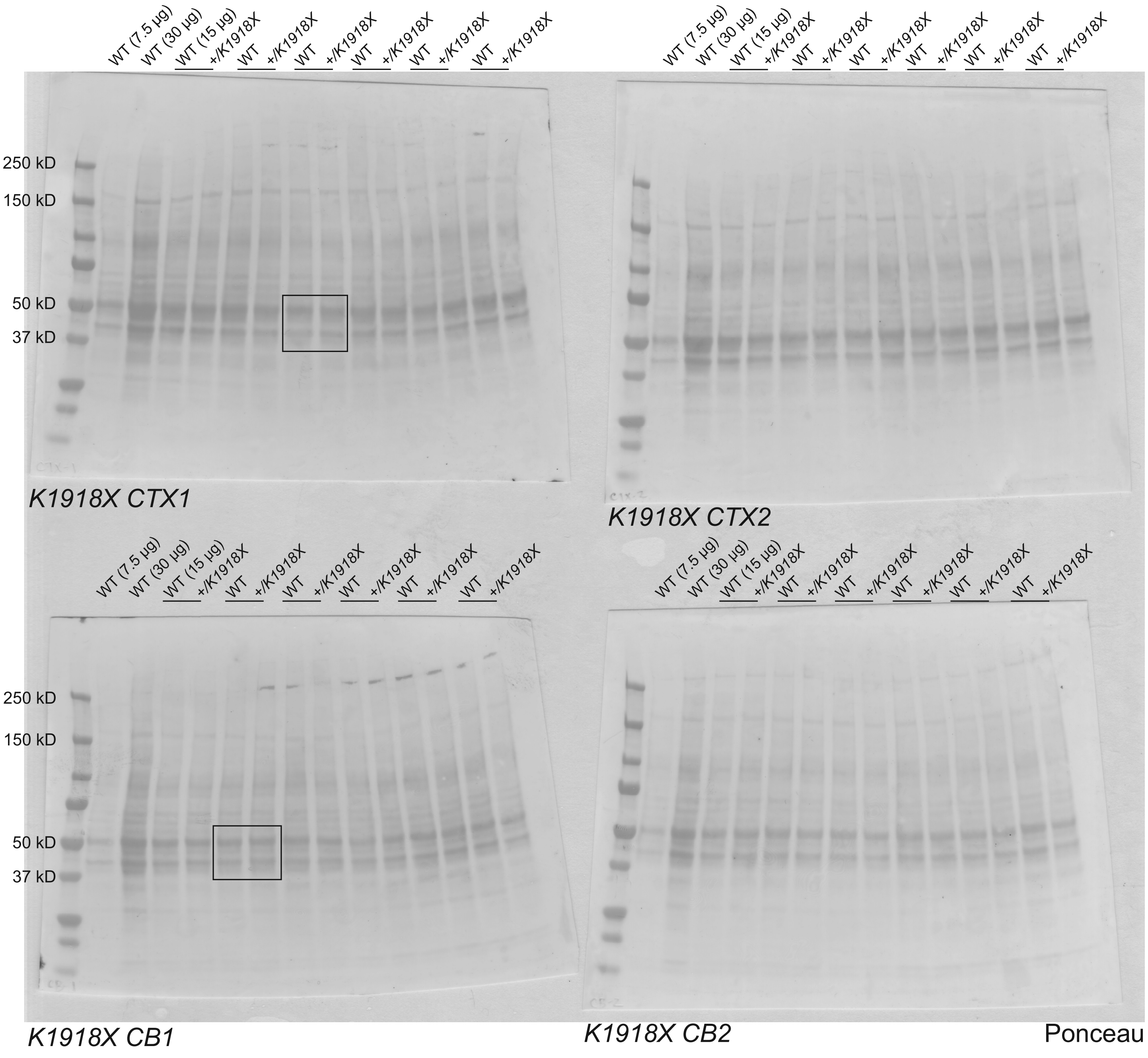

15 µg cortical (CTX) or cerebellar (CB) brain lysates from P42 paired littermate pups were separated by gel electrophoresis and stained by Ponceau S prior to blotting. 7.5 µg and 30 µg WT brain lysate were included as internal loading controls. Lines denote WT - *+/K1918X* littermate pairs.

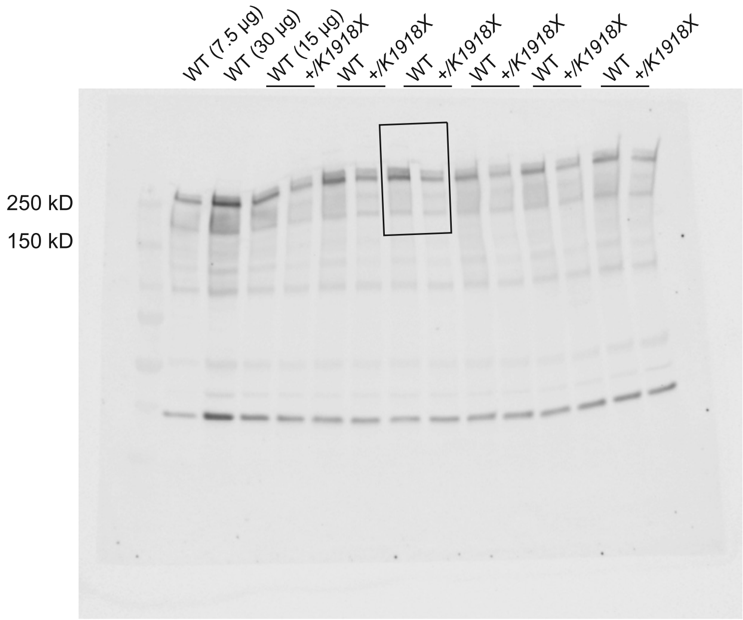

K1918X CTX1 WB: Trio SR5-6

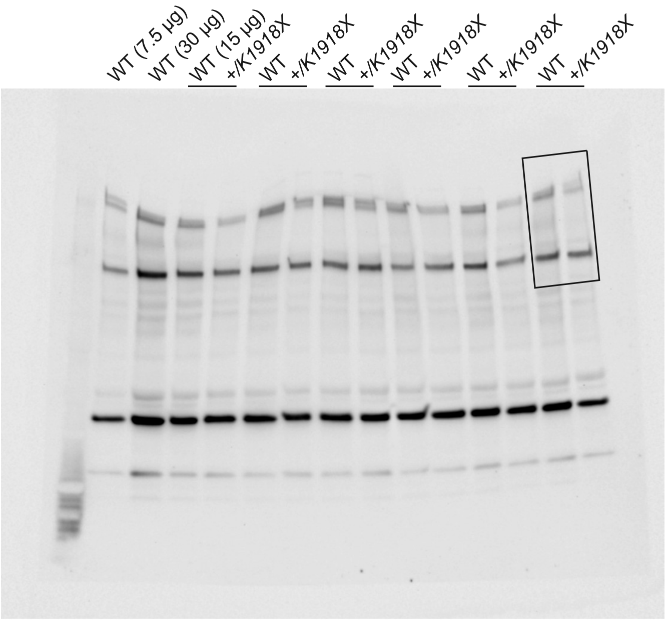

K1918X CTX2 WB: Trio DH2

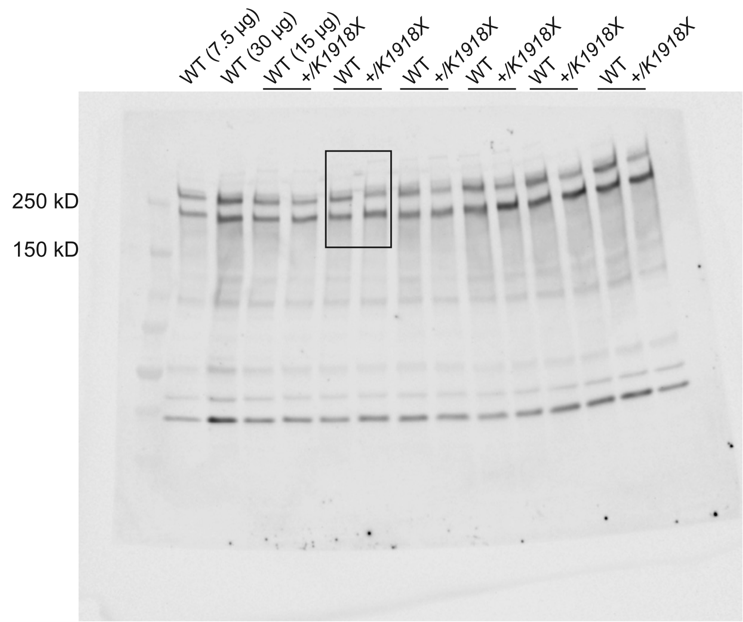

K1918X CB1 WB: Trio SR5-6

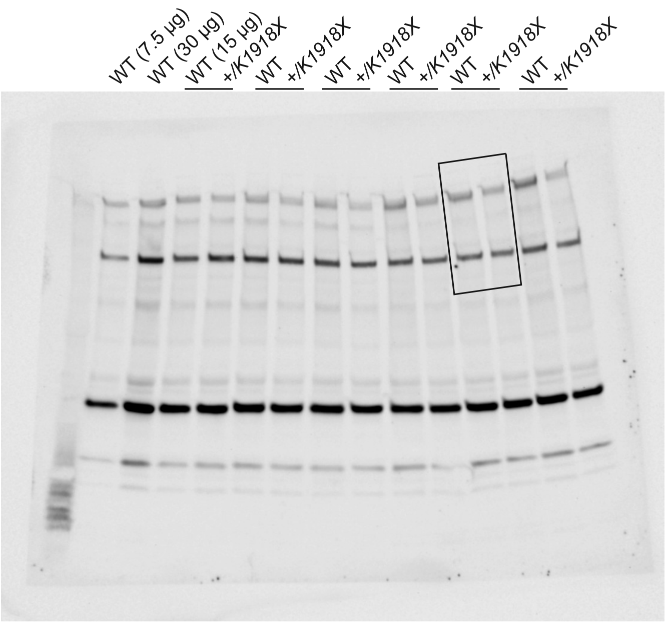

K1918X CB2 WB: Trio DH2

Membranes were blotted for Trio isoforms (Trio9S (263 kDa), Trio9L (277 kDa), Trio8 (217 kDa), Duet (145 kDa)) with an anti-Trio SR5-6 antibody (left) or anti-Trio DH2 antibody (right). Lines denote WT - *+/K1431M* littermate pairs.

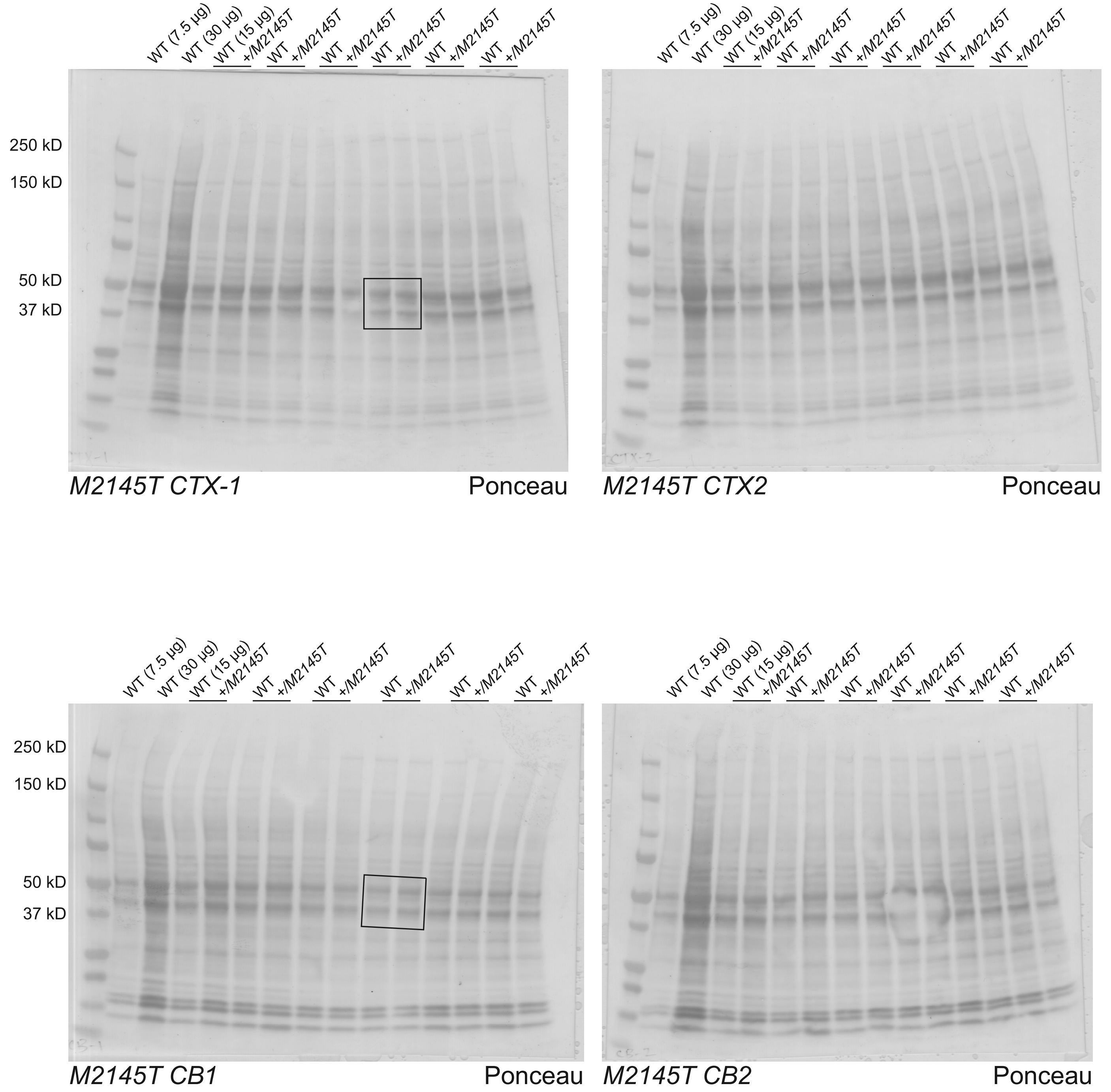

15 µg cortical (CTX) or cerebellar (CB) brain lysates from P42 paired littermate pups were separated by gel electrophoresis and stained by Ponceau S prior to blotting. 7.5 µg and 30 µg WT brain lysate were included as internal loading controls. Lines denote WT - *+/M2145T* littermate pairs.

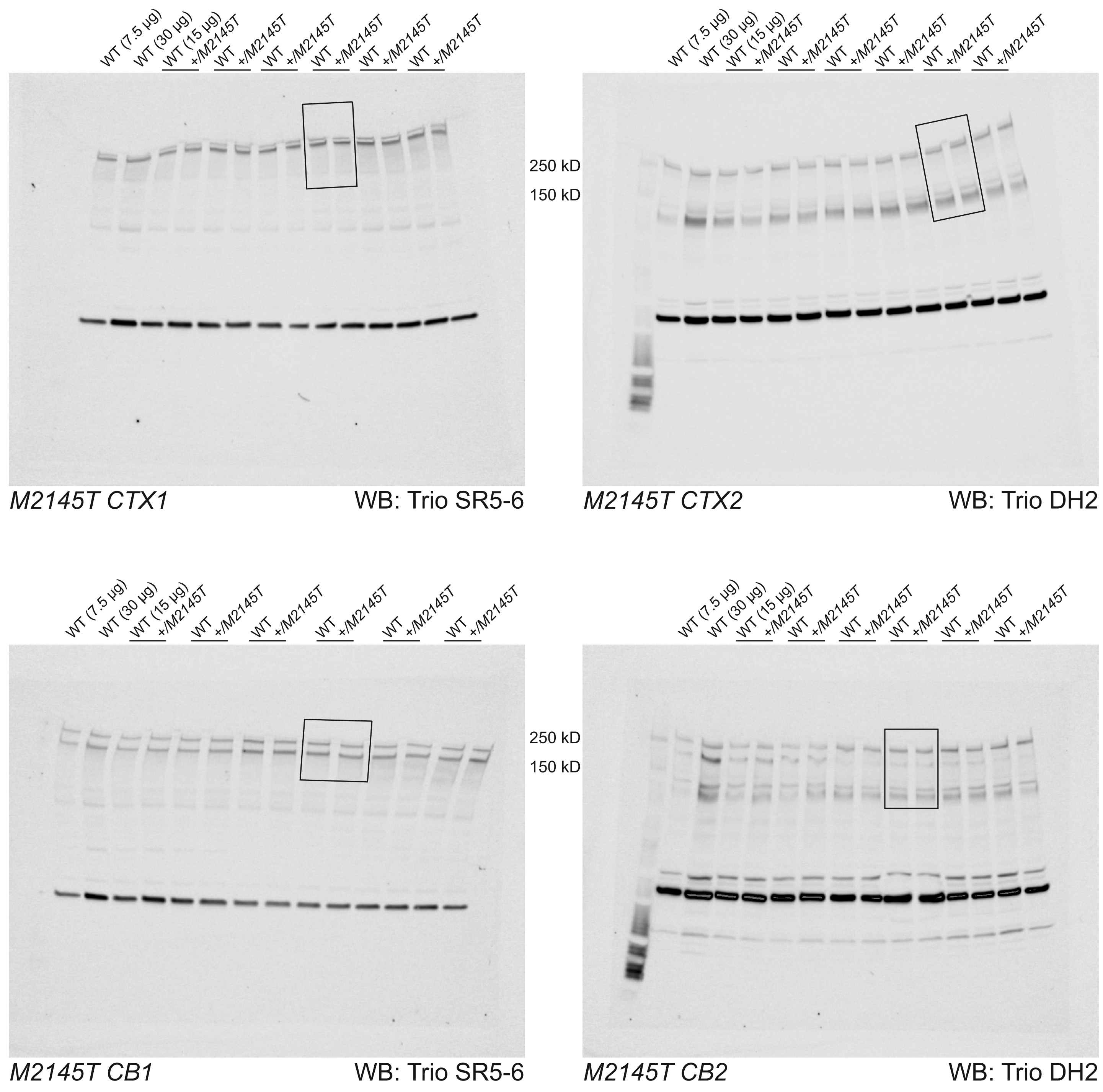

Membranes were blotted for Trio isoforms (Trio9S (263 kDa), Trio9L (277 kDa), Trio8 (217 kDa), Duet (145 kDa)) with an anti-Trio SR5-6 antibody (left) or anti-Trio DH2 antibody (right). Lines denote WT - *+/M2145T* littermate pairs.
