# Supplementary material for: Heterozygosity for neurodevelopmental disorder-associated TRIO variants yields distinct deficits in behavior, neuronal development, and synaptic transmission in mice
Source: eLife. 2025 Jun 9;13:RP103620. doi: 10.7554/eLife.103620 (PMC12148328; doi:10.7554/eLife.103620)
Supplement: Figure 1—figure supplement 1—source data 4. [file elife-103620-fig1-figsupp1-data4.zip › Figure 1-figure supplement 1-source data 4/Figure 1-figure supplement 1-source data 4.pdf]

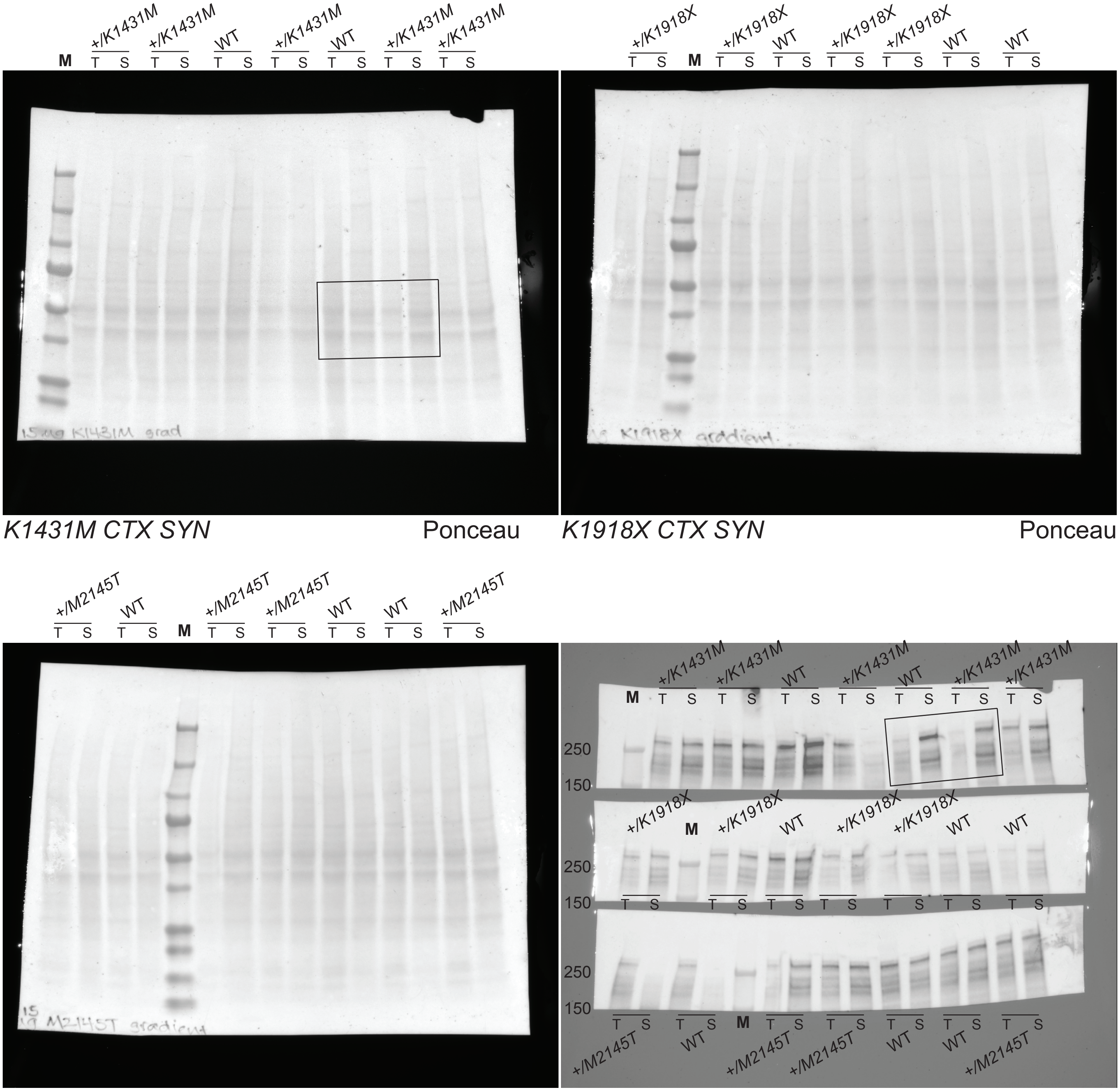

M2145T CTX SYN Ponceau

WB: Trio SR5-6

Figure 1-figure supplement 1-source data 4. Original membranes corresponding to Figure 1-figure supplement 1, Panel M.

15 µg cortical brain homogenate (T) and corresponding crude synaptosome preps (S) from P39-P42 mice were separated by gel electrophoresis and stained by Ponceau S. Lines denote total homogenate-synaptosome pairs. Stained membranes were cut prior to blotting with an anti-Trio SR5-6 antibody (bottom right). M: Bio-Rad Protein Plus All Blue Protein Standard. Boxes indicates cropped images used in final figure.

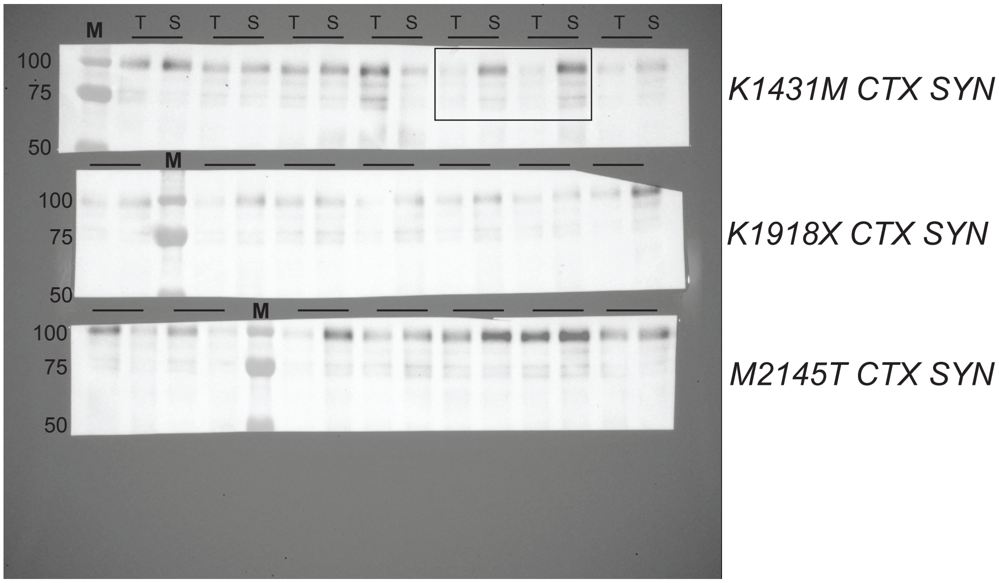

WB: PSD95

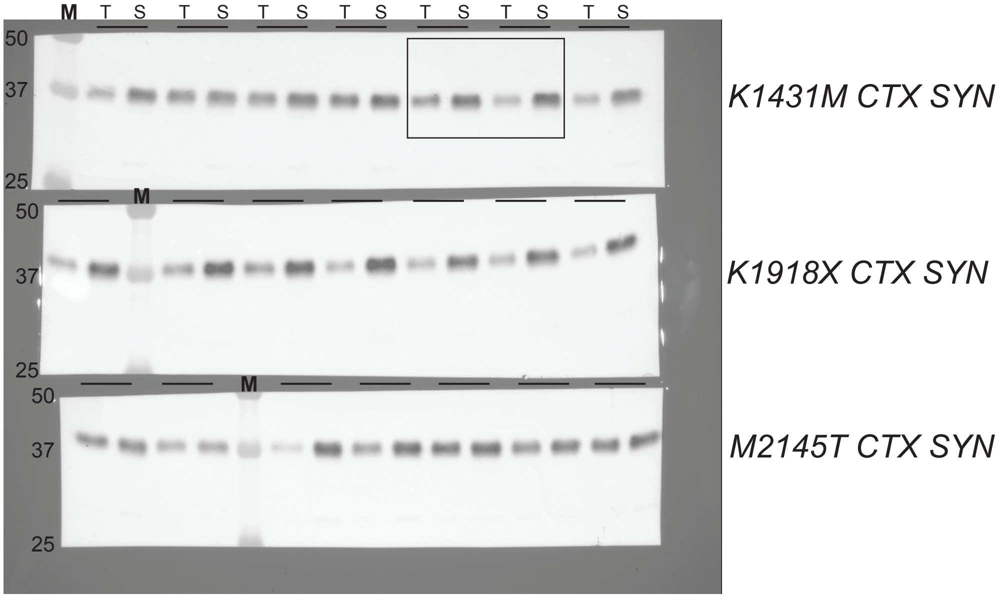

WB: Syp

1.5 µg cortical brain homogenate (T) and corresponding crude synaptosome preps (S) from P39-P42 mice were separated by gel electrophoresis; lines denote total homogenate-synaptosome pairs. Samples were loaded in the same order as in Trio blots on prior page. Ponceau-stained membranes were cut prior to immunoblotting for PSD95 (top) and synaptophysin (bottom). Boxes indicates cropped images used in final figure.
