# Supplementary material for: Heterozygosity for neurodevelopmental disorder-associated TRIO variants yields distinct deficits in behavior, neuronal development, and synaptic transmission in mice
Source: eLife. 2025 Jun 9;13:RP103620. doi: 10.7554/eLife.103620 (PMC12148328; doi:10.7554/eLife.103620)
Supplement: Figure 7—source data 4. [file elife-103620-fig7-data4.zip › Figure 7-source data 4/Figure 7-source data 4.pdf]

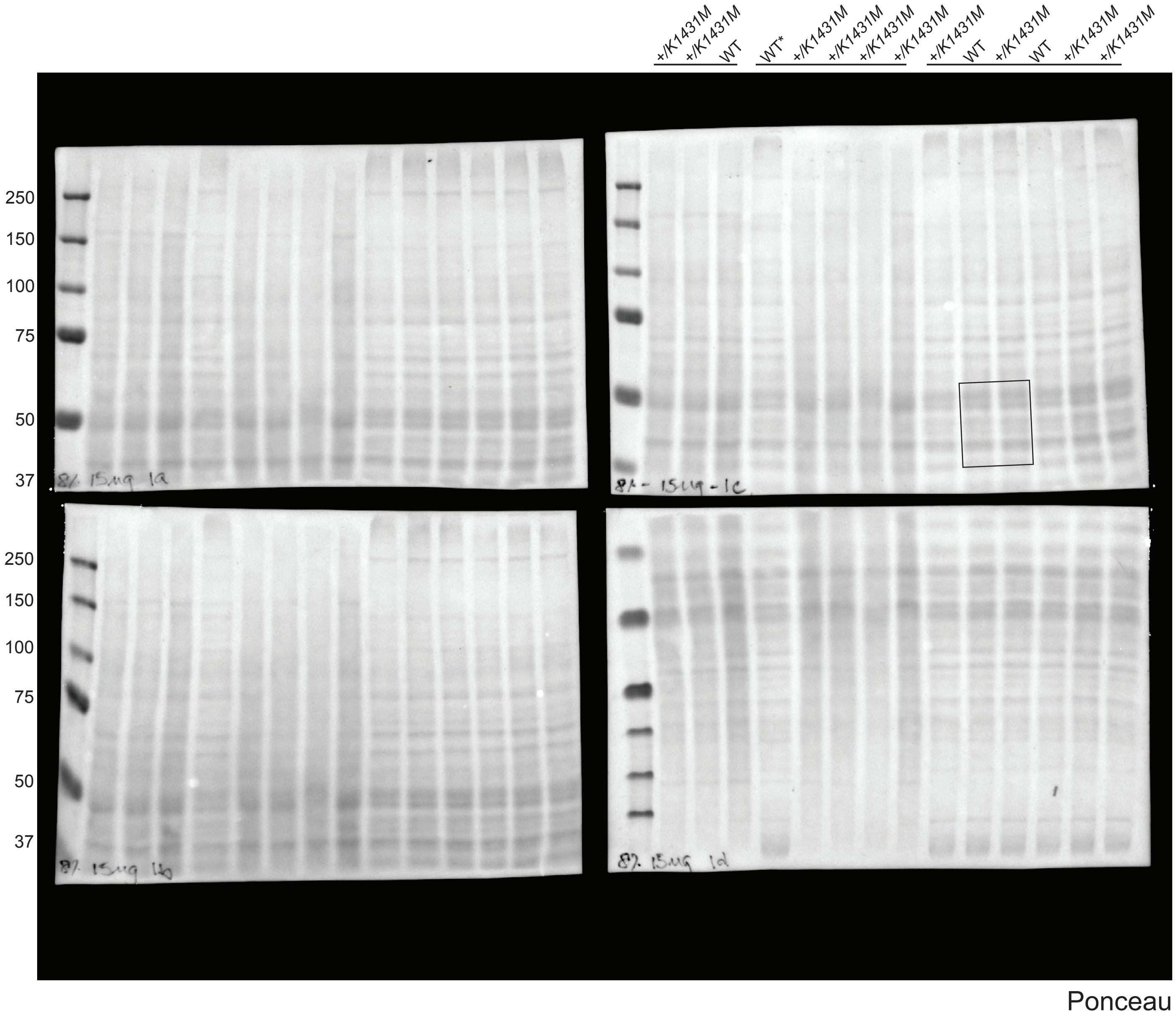

Figure 7-source data4. Original membranes corresponding to Figure 7, panel D.

15 µg cortical crude synaptosome preps from P39-P42 mice were separated by gel electrophoresis and stained by Ponceau S prior to immunoblotting. The same samples were run on four separate gels (1a, 1b, 1c, 1d) in the same loading order. WT* indicates internal loading control. Lines denote littermates. Box indicates cropped image used in final figure.

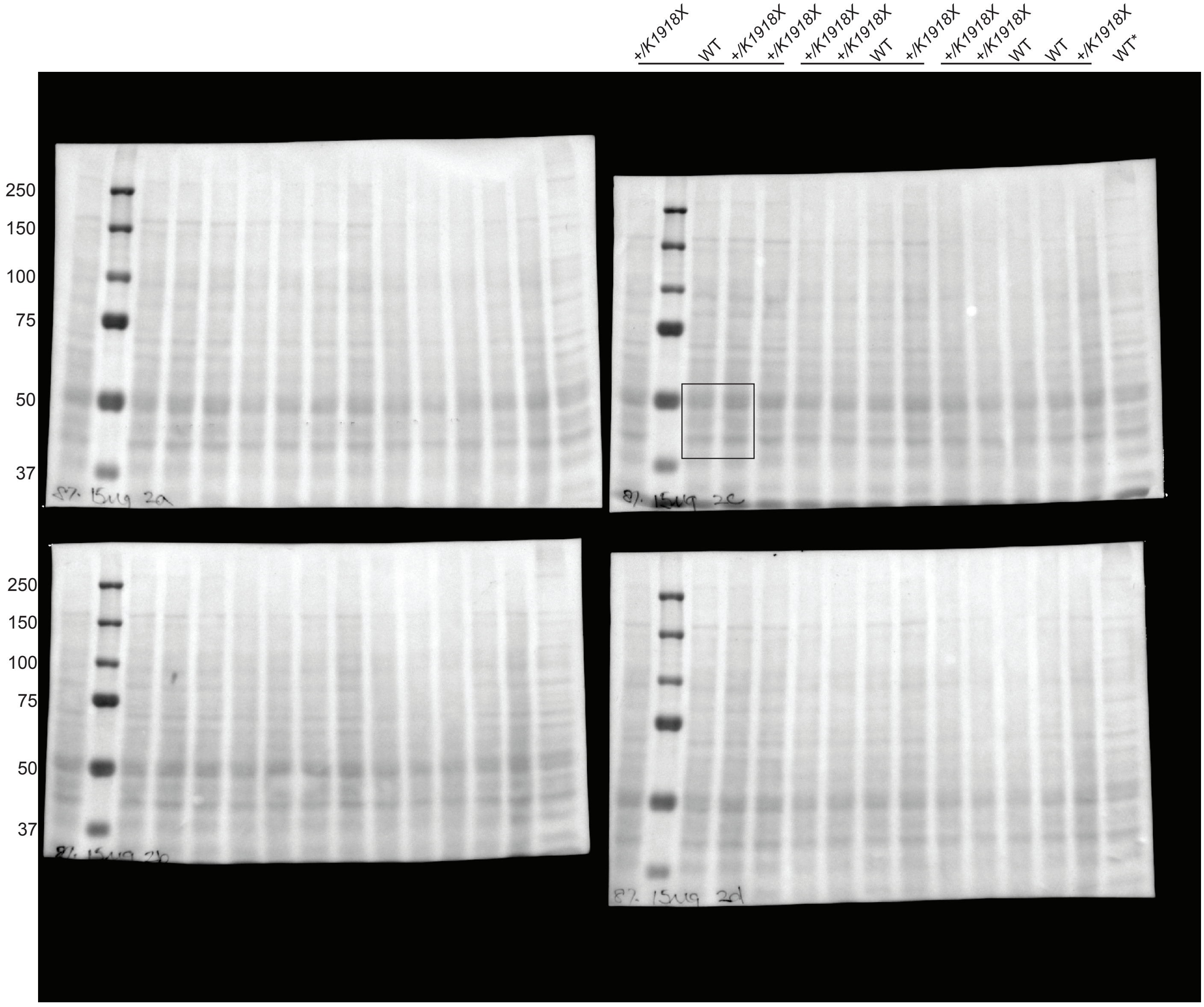

Ponceau

15 µg cortical crude synaptosome preps from P39-P42 mice were separated by gel electrophoresis and stained by Ponceau S prior to immunoblotting. The same samples were run on four separate gels (2a, 2b, 2c, 2d) in the same loading order. WT* indicates internal loading control. Lines denote littermates. Box indicates cropped image used in final figure.

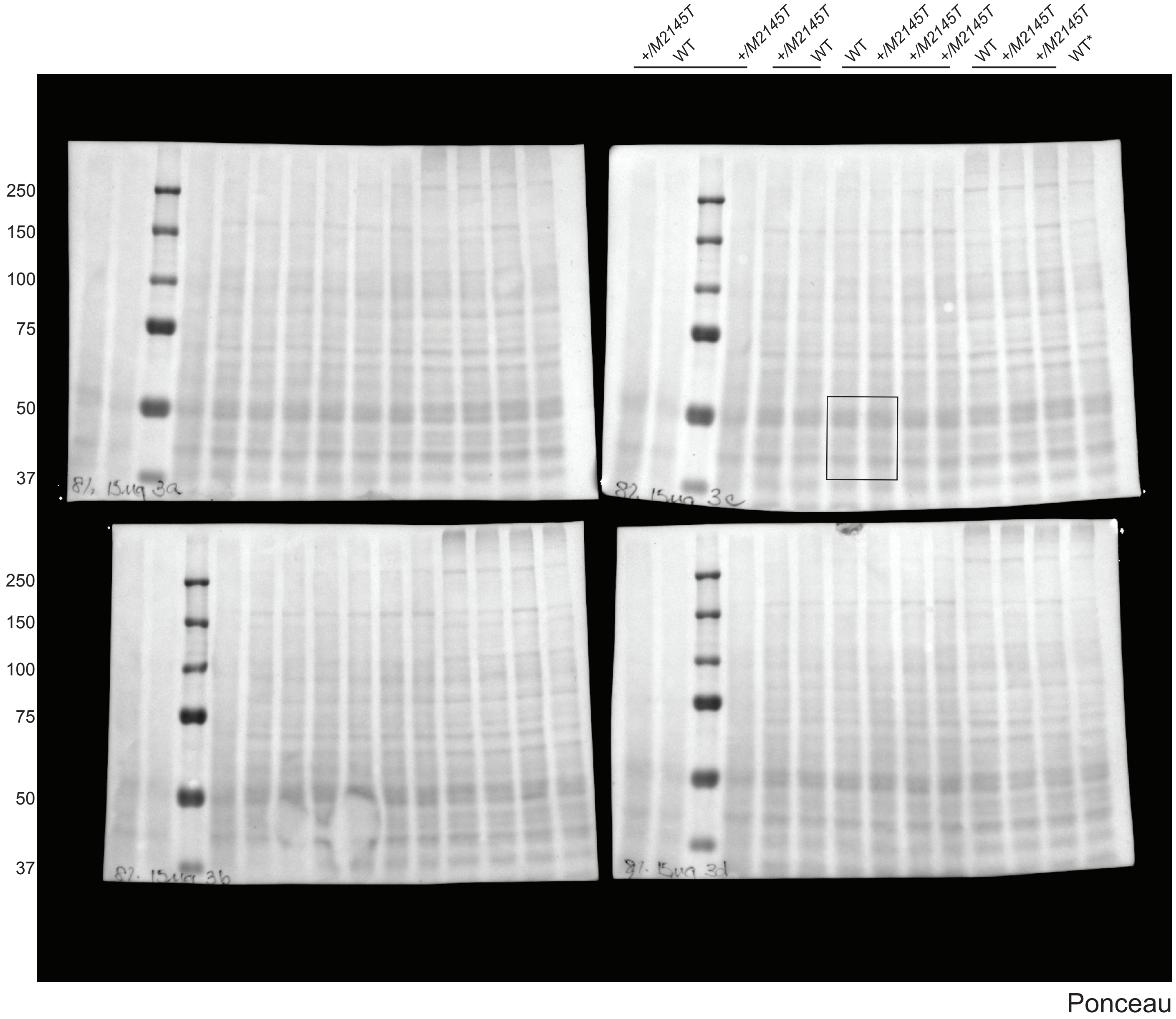

15 µg cortical crude synaptosome preps from P39-P42 mice were separated by gel electrophoresis and stained by Ponceau S prior to immunoblotting. The same samples were run on four separate gels (3a, 3b, 3c, 3d) in the same loading order. WT* indicates internal loading control. Lines denote littermates. Box indicates cropped image used in final figure.

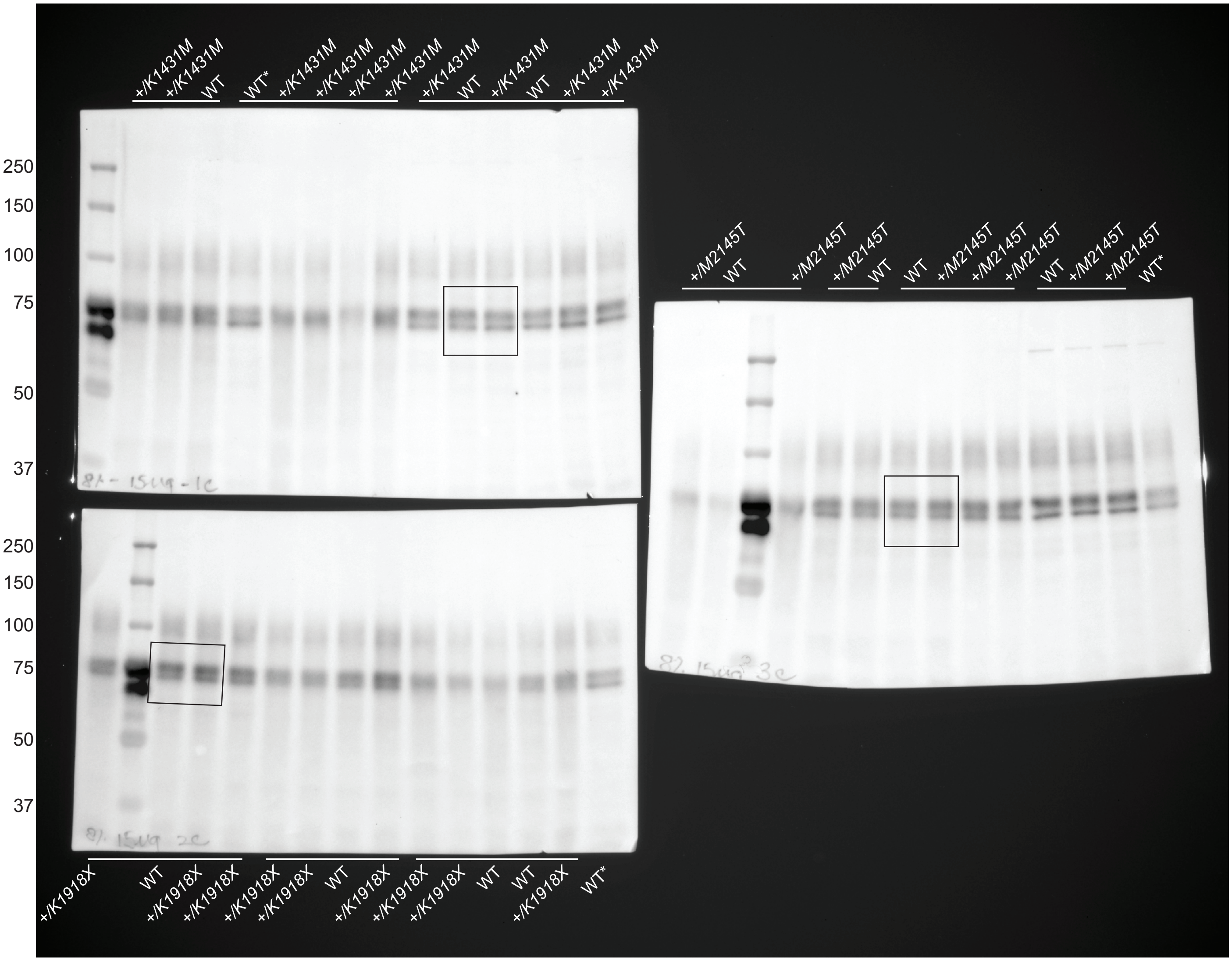

WB: Syt3

15 µg cortical crude synaptosome preps from P39-P42 mice were separated by gel electrophoresis and immunoblotted for synaptotagmin3 (Syt3). WT* indicates internal loading control. Lines denote littermates. Boxes indicate cropped images used in final figure.

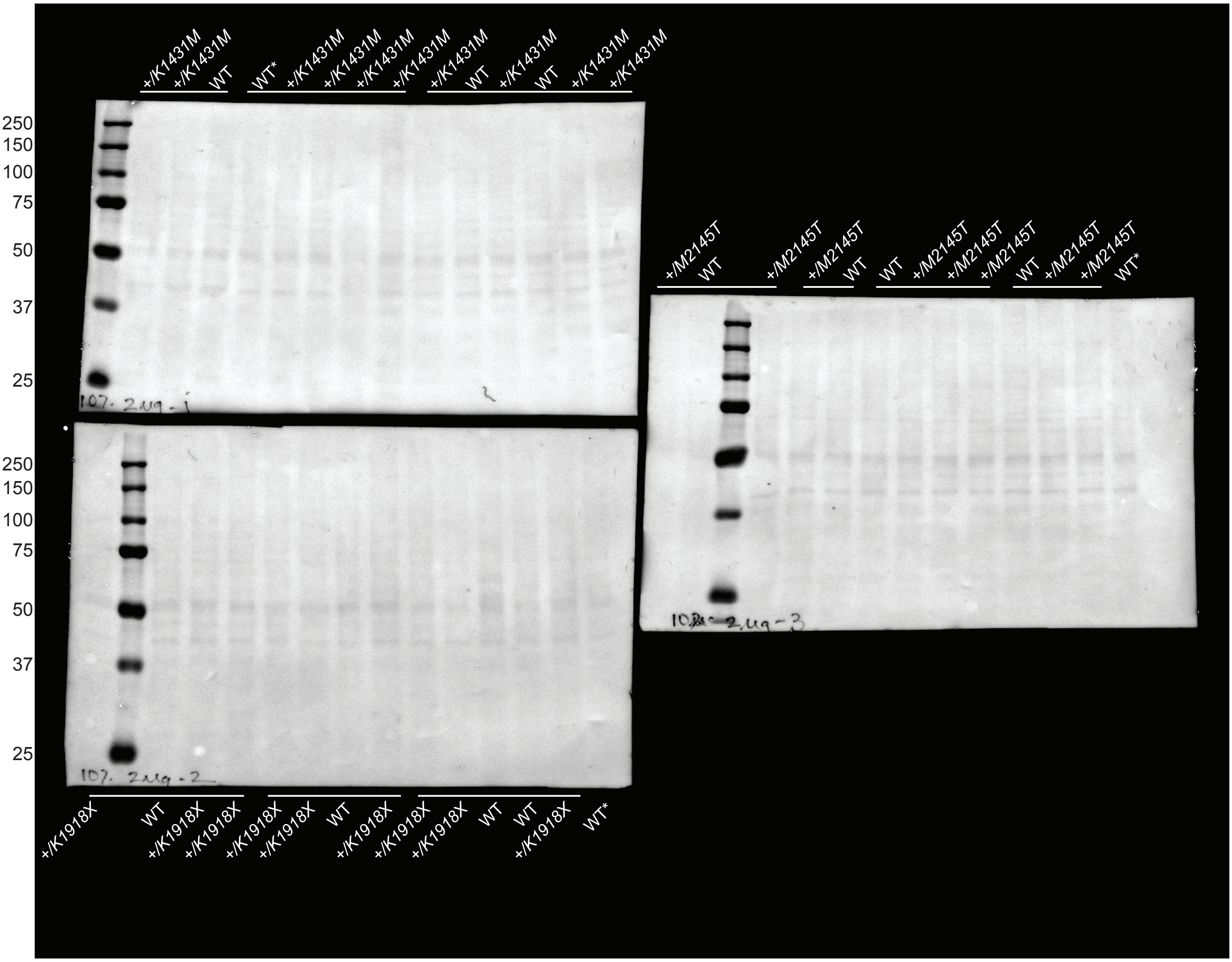

Ponceau

2 μg cortical crude synaptosome preps from P39-P42 mice were separated by gel electrophoresis and stained by Ponceau S prior to immunoblotting. WT* indicates internal loading control. Lines denote littermates. Stained membranes were cut prior to blotting. Note: these membranes were used for normalization of Western blots (Munc18, Syp) but were not shown in final figures.

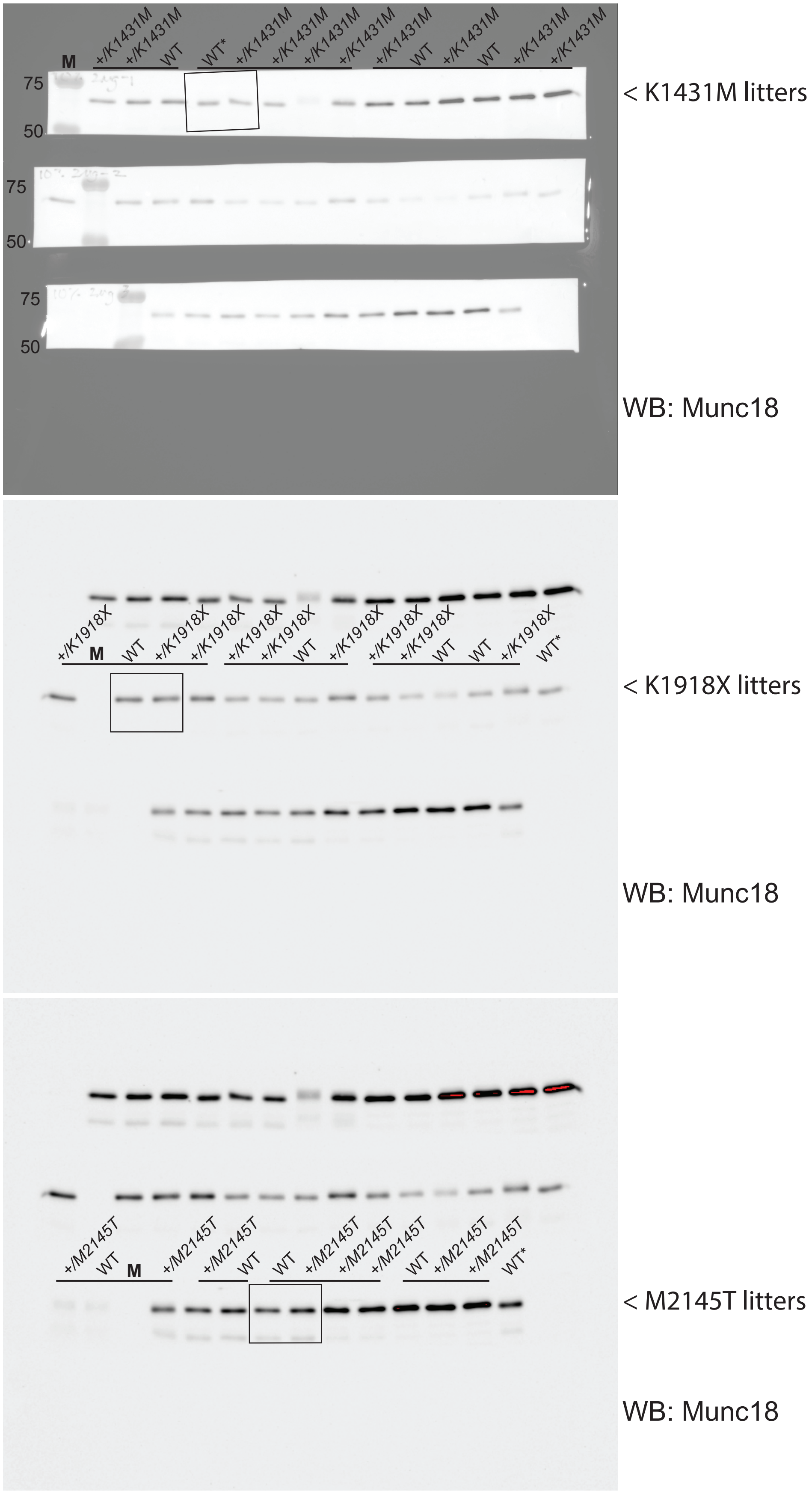

2 µg cortical crude synaptosome preps from P39-P42 mice were separated by gel electrophoresis and immunoblotted for syntaxin binding protein 1 (Stxbp1, also known as Munc18-1). Lines denote littermates. The same blot at different exposures (30-60 sec) are shown. Boxes indicate cropped images used in final figure. M: Bio-Rad Protein Plus All Blue Protein Standard.

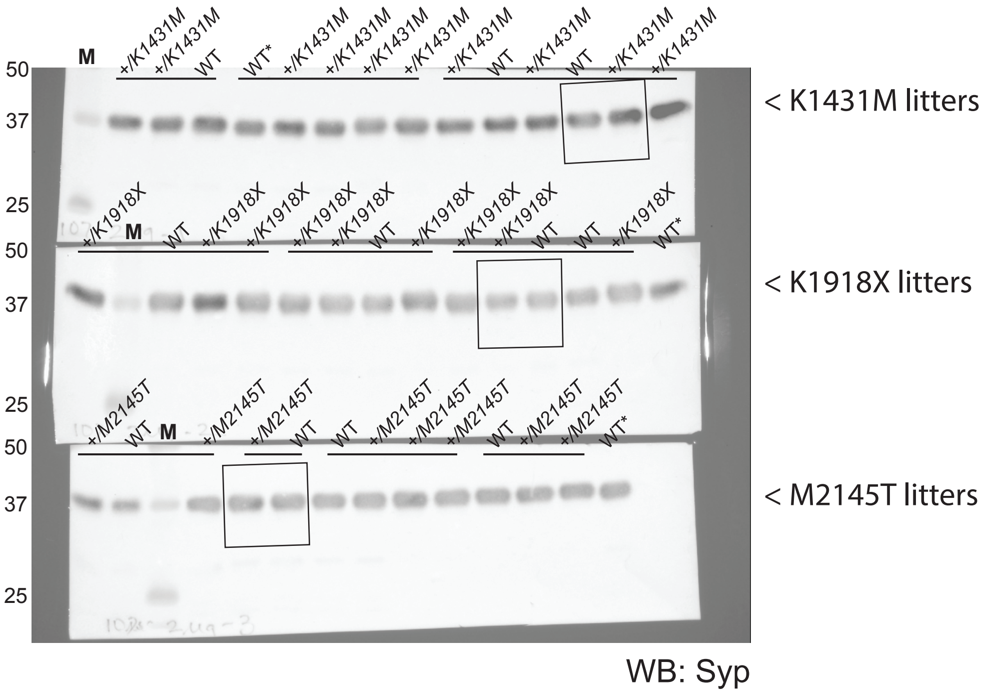

2 µg cortical crude synaptosome preps from P39-P42 mice were separated by gel electrophoresis and immunoblotted for synaptophysin (Syp). WT* indicates internal loading control. Lines denote littermates. Boxes indicate cropped images used in final figure. M: Bio-Rad Protein Plus All Blue Protein Standard.

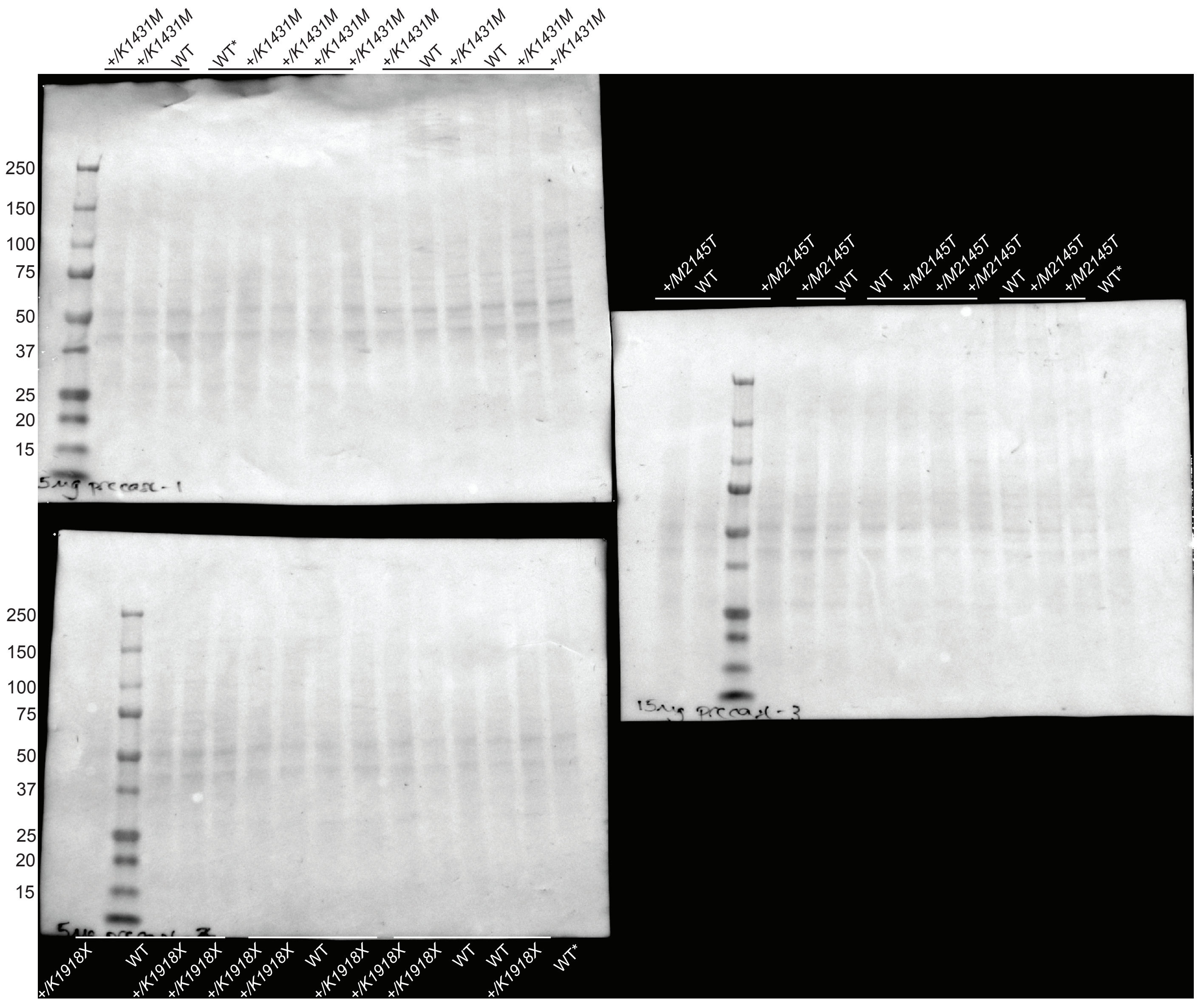

Ponceau

5 µg cortical crude synaptosome preps from P39-P42 mice were separated by gel electrophoresis and stained by Ponceau S prior to immunoblotting. WT* indicates internal loading control. Lines denote littermates. Stained membranes were cut prior to blotting. Note: these membranes were used for normalization of Western blots (Stx1a) but were not shown in final figures.

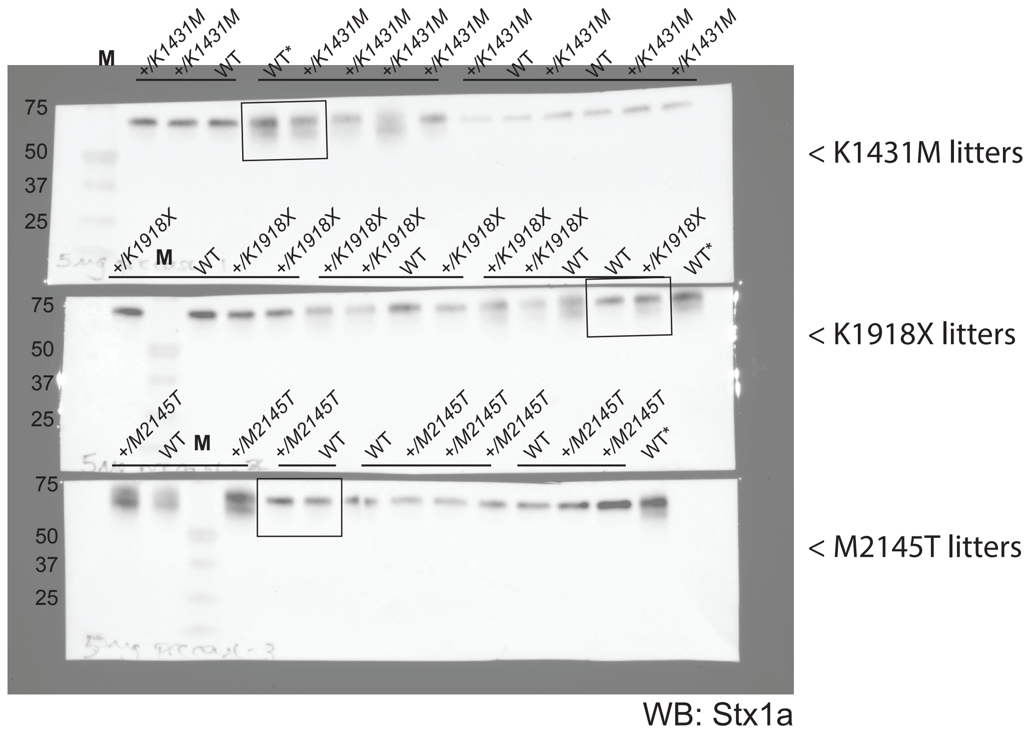

5 µg cortical crude synaptosome preps from P39-P42 mice were separated by gel electrophoresis and immunoblotted for syntaxin1a (Stx1a). WT* indicates internal loading control. Lines denote littermates. Boxes indicate cropped images used in final figure. M: Bio-Rad Protein Plus All Blue Protein Standard.
