# Supplementary material for: Heterozygosity for neurodevelopmental disorder-associated TRIO variants yields distinct deficits in behavior, neuronal development, and synaptic transmission in mice
Source: eLife. 2025 Jun 9;13:RP103620. doi: 10.7554/eLife.103620 (PMC12148328; doi:10.7554/eLife.103620)
Supplement: Figure 7—source data 6. [file elife-103620-fig7-data6.zip › Folder 7-source data 6/Figure 7-source data 6.pdf]

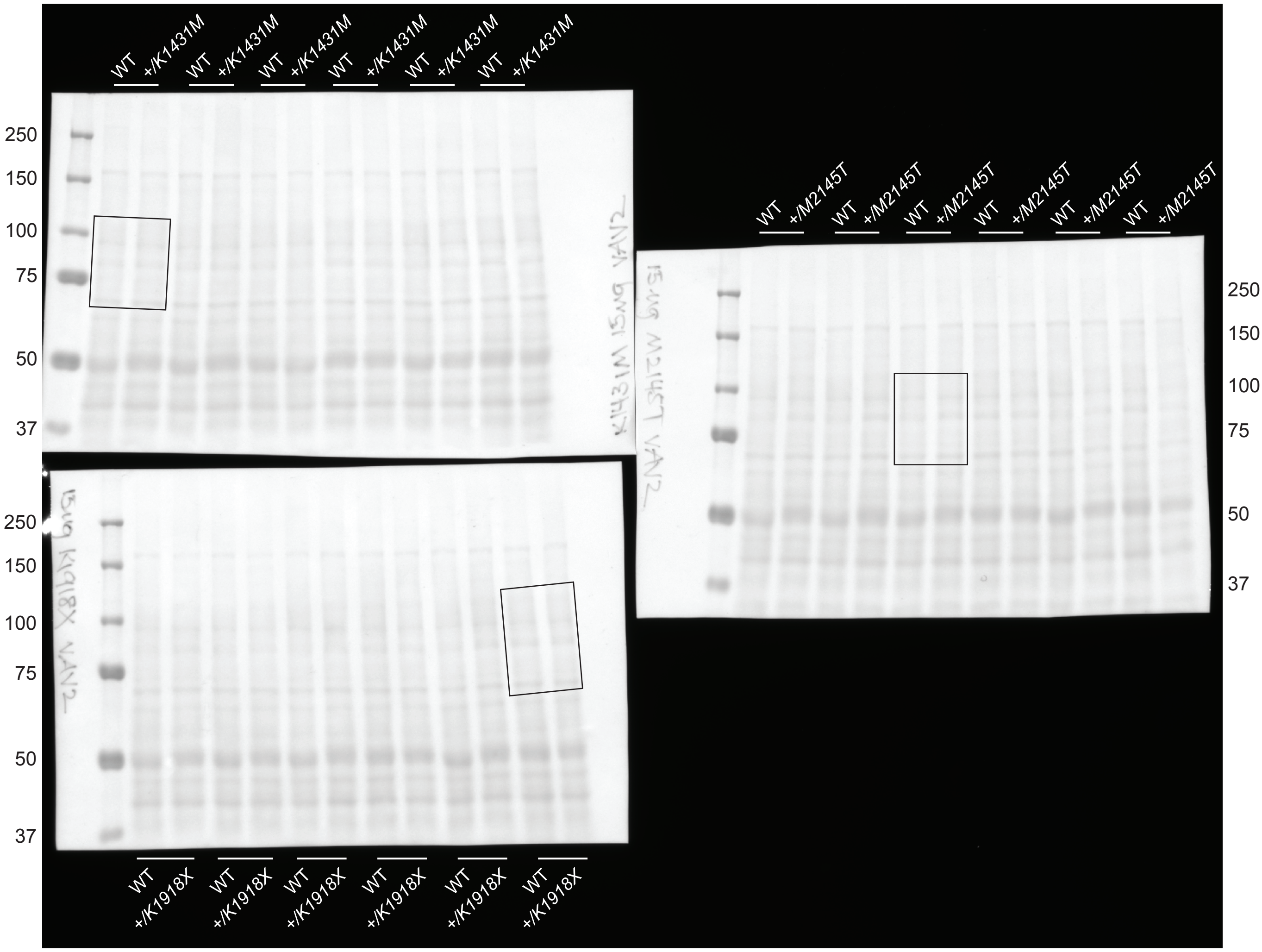

Figure 7-source data6. Original membranes corresponding to Figure 7, panel I.

15 µg cortical brain lysates from P42 paired littermate pups were separated by gel electrophoresis and stained by Ponceau S prior to blotting for Vav3. Lines denote littermate pairs. Boxes indicates cropped

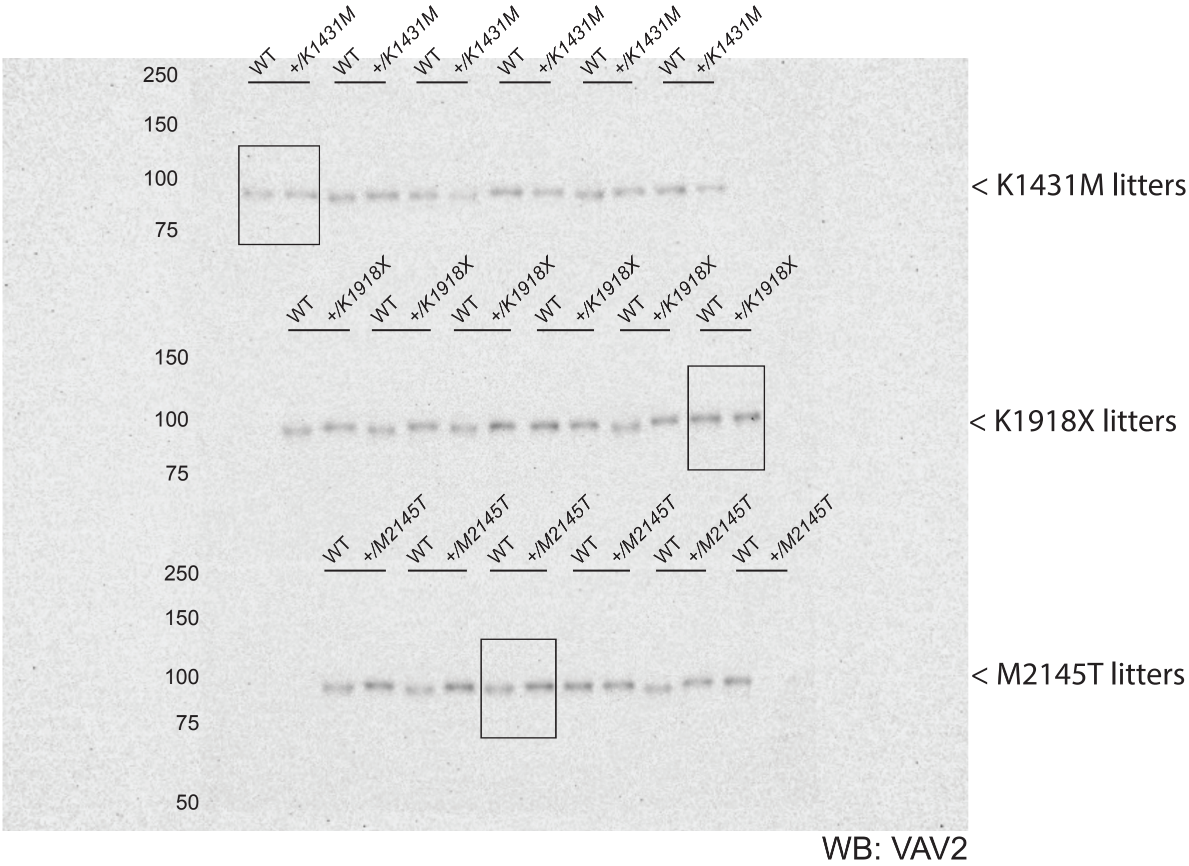

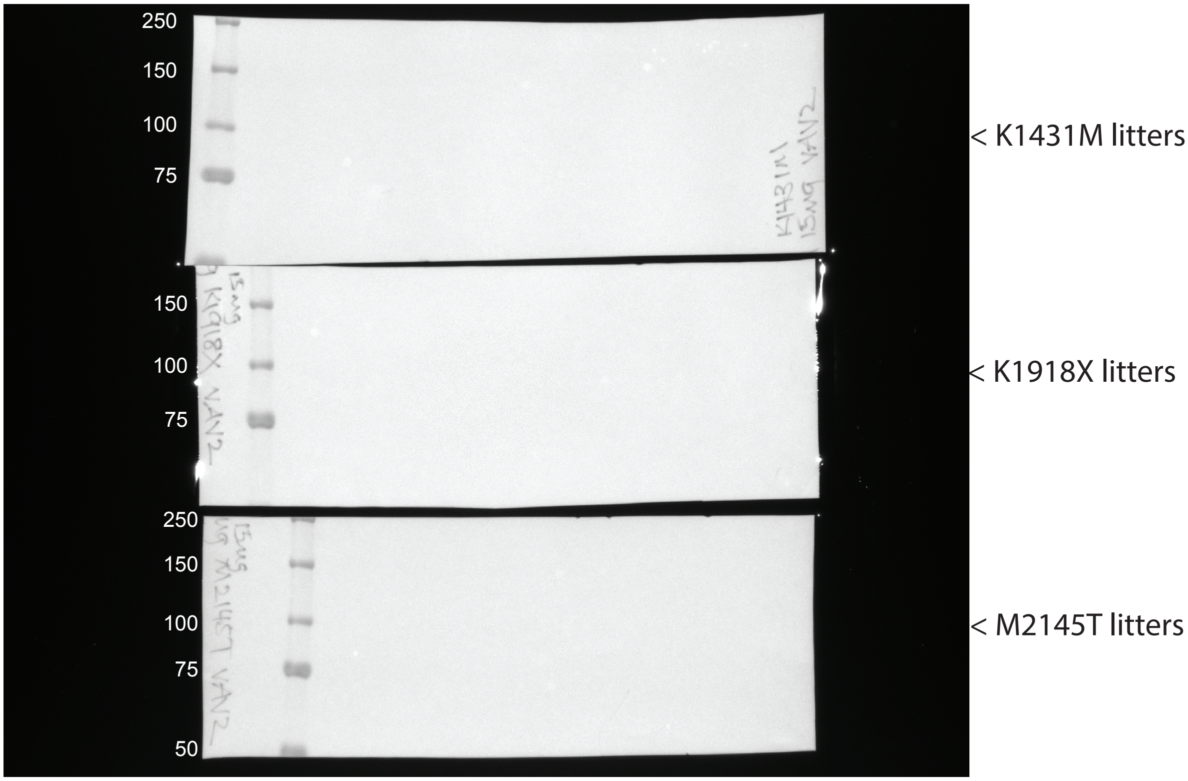

15 µg cortical brain lysates from P42 paired littermate pups were separated by gel electrophoresis and immunoblotted for Vav3 (top). Lines denote littermate pairs. Boxes indicates cropped images used in final figure. Note: colorimetric image from corresponding blot is shown here (bottom) to illustrate band size but is not included in final figure.

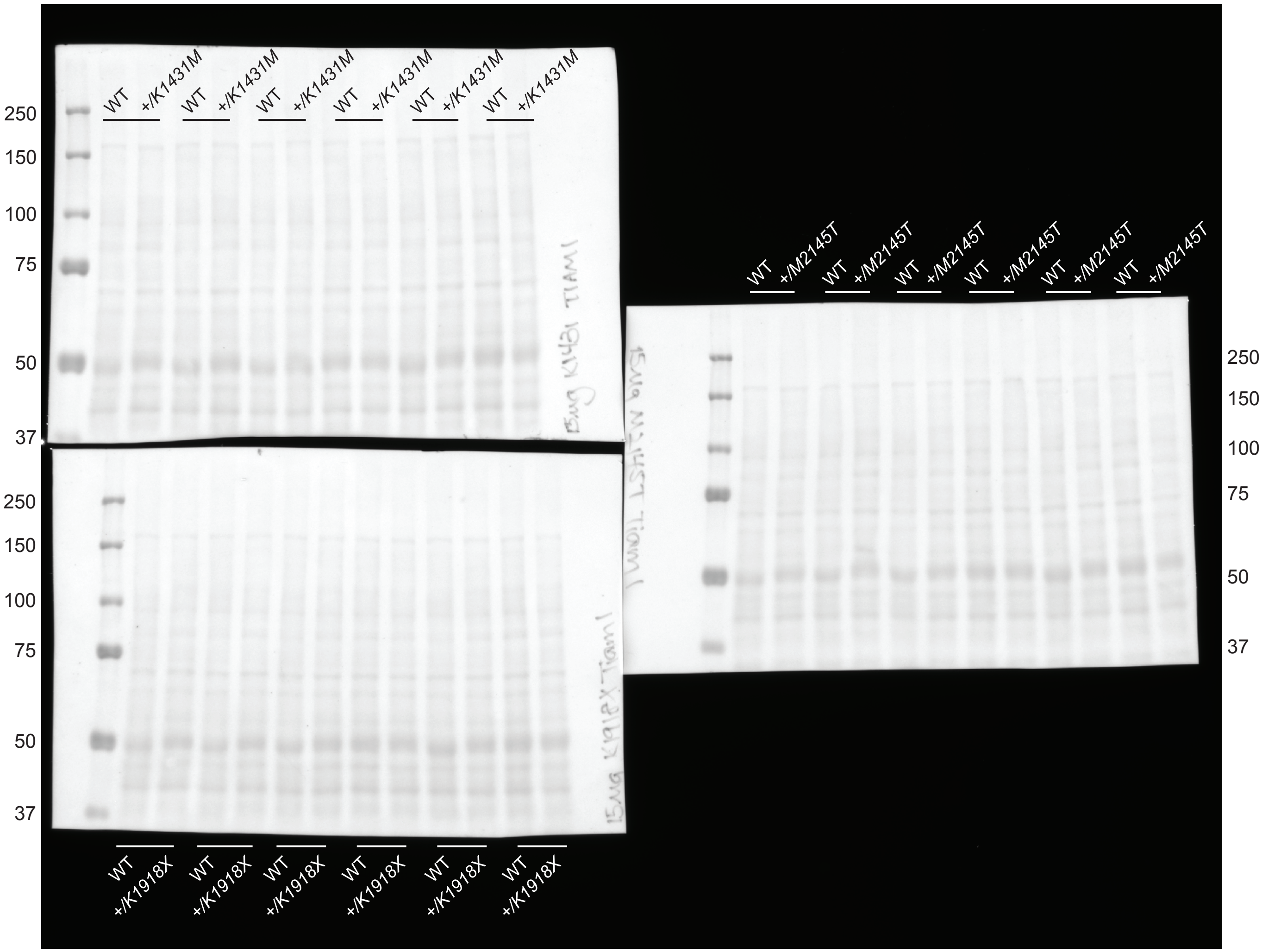

Ponceau

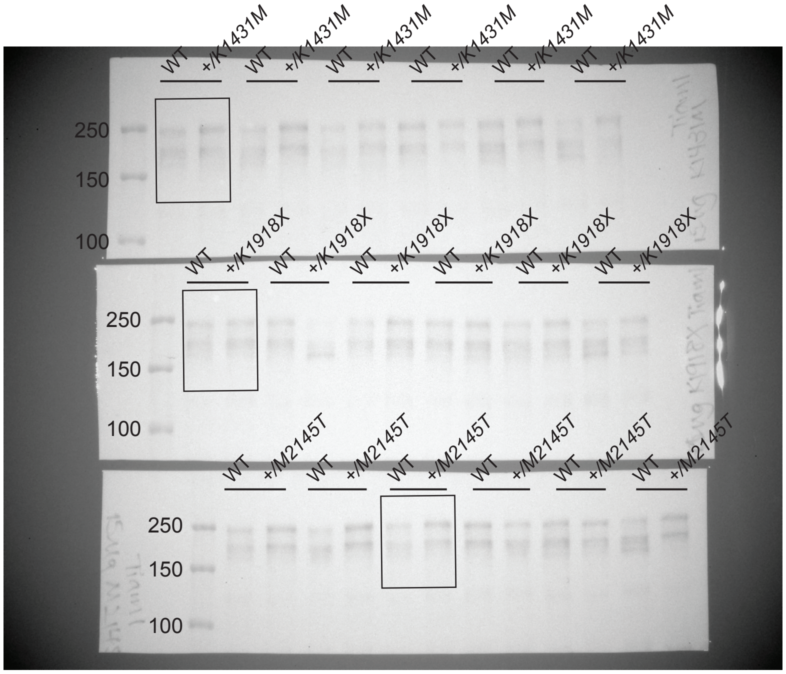

WB: Tiam1

15 µg cortical brain lysates from P42 paired littermate pups were separated by gel electrophoresis and stained by Ponceau S (top), then immunoblotted for Tiam1 (bottom). Lines denote littermate pairs. Boxes indicates cropped images used in final figure. Note: Ponceau membranes were used for normalization of Western blots but were not shown in final figures.

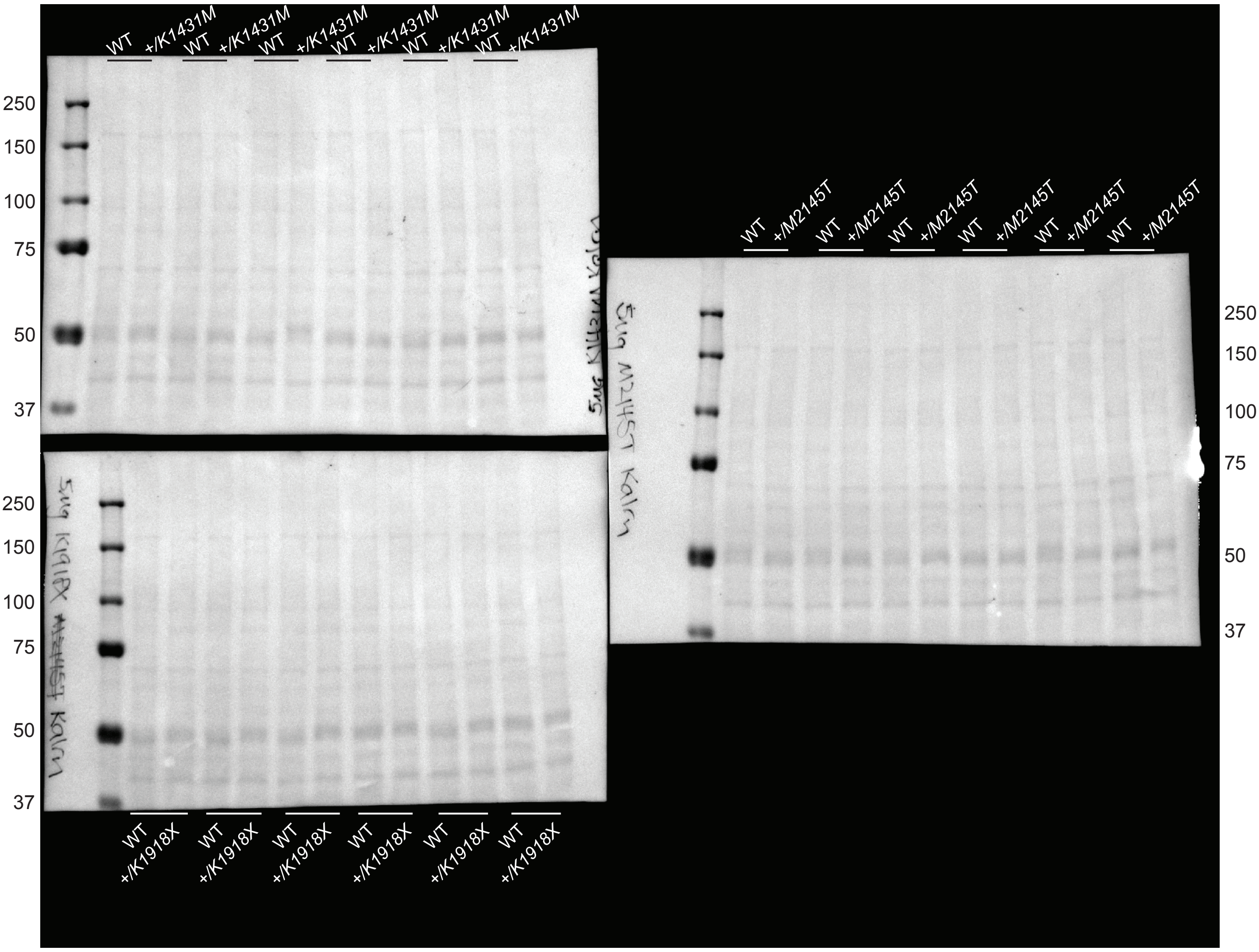

Ponceau

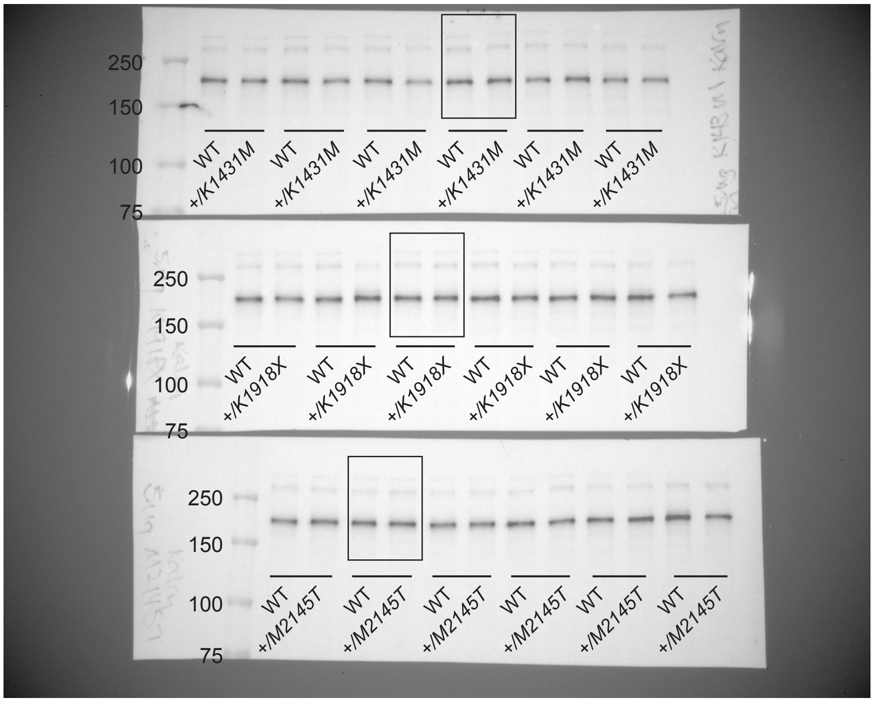

WB: Kalrn

5 µg cortical brain lysates from P42 paired littermate pups were separated by gel electrophoresis and stained by Ponceau S (top), then immunoblotted for Kalirin (bottom). Lines denote littermate pairs. Boxes indicates cropped images used in final figure. Note: Ponceau membranes were used for normalization of Western blots but were not shown in final figures.
